# Data-driven identification of heart failure disease states and progression pathways using electronic health records

**DOI:** 10.1038/s41598-022-22398-4

**Published:** 2022-10-25

**Authors:** Tasha Nagamine, Brian Gillette, John Kahoun, Rolf Burghaus, Jörg Lippert, Mayur Saxena

**Affiliations:** 1 Droice Research, New York, NY USA; 2 Department of Surgery, NYU Langone Long Island, Mineola, NY USA; 3 Department of Foundations of Medicine, NYU Long Island School of Medicine, Mineola, NY USA; 4 Clinical Informatics, CityMD, New York, NY USA; 5 Bayer AG, Wuppertal, Germany

**Keywords:** Heart failure, Heart failure, Computational models

## Abstract

Heart failure (HF) is a leading cause of morbidity, healthcare costs, and mortality. Guideline based segmentation of HF into distinct subtypes is coarse and unlikely to reflect the heterogeneity of etiologies and disease trajectories of patients. While analyses of electronic health records show promise in expanding our understanding of complex syndromes like HF in an evidence-driven way, limitations in data quality have presented challenges for large-scale EHR-based insight generation and decision-making. We present a hypothesis-free approach to generating real-world characteristics and progression patterns of HF. Patient disease state snapshots are extracted from the complaints mentioned in unstructured clinical notes. Typical disease states are generated by clustering and characterized in terms of their distinguishing features, temporal relationships, and risk of important clinical events. Our analysis generates a comprehensive “disease phenome” of real-world patients computed from large, noisy, secondary-use EHR datasets created in a routine clinical setting.

## Introduction

Heart failure (HF) is a major contributor to global disease burden, having a worldwide estimated prevalence of 64 million patients and staggering associated health and financial costs of 10 million years lost due to disability (YLDs) and $350 billion US in expenditure^1^. Despite recent innovations in pharmaceutical interventions and clinical management strategies, HF continues to be difficult to treat, as HF patients are often medically complex and present with diverse phenotypes characterized by a variety of pathophysiological mechanisms, clinical measurements, biomarkers, lifestyle factors, comorbidities, and treatment responses^2,3^. This complexity poses a challenge for defining precise HF classifications that can serve as the basis for HF research and treatment standards. Current guidelines for the diagnosis and treatment of HF have highlighted the fact that there is no single agreed upon classification system for causes of HF, with significant overlap across diverse etiologies^4^. Moreover, the myriad ways in which HF evolves are poorly understood, with many patients showing only mild or no clinical symptoms as underlying disease worsens^5^, and so it can be particularly challenging to identify which HF patients are at high risk for progression and poor prognosis^6,7^.

Unfortunately, the constellation of current top-down HF classification and risk stratification systems based on biomarkers such as lab values^8,9^ and left ventricular ejection fraction (LVEF)^10^ or functional assessments such as the New York Heart Association (NYHA) classification^11^ oversimplify the complexity of HF and do not adequately represent the diversity of disease states and how they progress over time^10,11^. These limitations have left the pharmaceutical community struggling to develop effective risk-enrichment strategies for clinical trials and to determine the appropriate timing for therapeutic interventions without limiting future drug labels to small sub-populations with limited relevance^12^. Currently it is standard practice to retrospectively analyze failed or successful phase III trials in order to identify patient groups that would benefit from specific treatments. The investigation of the atrial fibrillation (AF) patient subcohort in the failed TOPCAT trial for HF with preserved ejection fraction (HFpEF) is just one example. Patients with a history of AF or AF at enrollment showed increased risk of cardiovascular morbidity and mortality, but no modified response to treatment^13^. Even if successful, such retrospective analyses can only generate hypotheses for secondary pivotal trials following primary phase III trials, thus requiring massive additional investment in time, money, and patient lives. The gaps in understanding of HF subtypes have also made it difficult for healthcare providers to find optimal treatments for HF patients with comorbid conditions that complicate management. For example, chronic kidney disease (CKD) affects 40–60% of HF patients and is a significant predictor of poor outcomes^14,15^ yet is underrepresented in general HF studies, leading to insufficient evidence regarding benefits and harms of HF drugs for patients with advanced CKD^16^. An improved understanding of clinically meaningful HF phenotypic differences and the patterns of HF disease state progression could lead to more targeted and subpopulation-specific therapeutic strategies, as well as earlier identification of patients at high risk for worsening HF, enabling the initiation of preemptive treatments to avoid or slow progression to irreversible end-stage disease HF^17–19^.

Data-driven approaches using data generated outside of a randomized controlled trials (RCTs), known as real-world data (RWD), have the potential to transform practice-based observations into evidence that can aid in bridging the gap between clinical science and practice^20^. Importantly, electronic health records (EHRs) are becoming increasingly popular as the source for data-driven and machine learning approaches to address many problems in healthcare^21^, including models for predicting risk in HF^22–26^. At the same time, there is a growing acknowledgement that patient-centric, data-driven methods to uncover latent patterns within patient populations offer advantages over disease-centric, bottom-up, or simple predictive frameworks for disease understanding and care personalization at a higher level, particularly within complex and multimorbid patient populations^27,28^ such as those found in HF. With this in mind, a growing body of work has utilized hypothesis-free, data-driven approaches identify groups of similar patients and disease trajectories using targeted clinical markers or data elements from curated registry or EHR data in a variety of disease areas^29–33^. However, most of these studies have relied on structured data elements; advancements in natural language processing have made it possible to increasingly utilize EHR clinical notes, which contain detailed information that reflects the clinician’s view of a patient’s disease state and severity beyond structured data and lead to additional insights or increased performance in a variety of use cases^21,34–38^.

We have previously demonstrated that unsupervised clustering of HF patients’ disease and symptom-related concepts derived from natural language processing (NLP) of unstructured notes from the entirety of their longitudinal EHR can reveal the etiology, defining characteristics, and hierarchical relationships of HF phenotypes^39^. Based on these results, we hypothesized that clustering of clinical concepts from discrete “snapshots” that represent the patients’ disease states over time would reveal a comprehensive picture of the variety of HF manifestations and patterns of evolution dynamics across the population, leading to a data-driven picture of HF that captures the nuance of symptom expression and disease severity. In this study, we developed a hypothesis-free approach to elucidating the diversity of HF manifestation and progression patterns in a cohort of over 25 thousand HF patients treated at a single health center. We used unsupervised clustering to group 30-day patient snapshots into similar HF *disease states* and identified mathematically stable HF disease state clusters that revealed the dominant patterns of HF clinical manifestations, etiologies, and healthcare consumption at discrete points in the HF disease timeline. To reveal the dynamics of disease progression, healthcare utilization, and clinically important events within the HF population, we statistically analyzed disease state transitions within patient timelines to reveal common temporal networks of HF progression. Significantly, we found that the present and future probability of important clinical events of interest (e.g., ischemic stroke, acute decompensation, and in-hospital mortality) was enriched within certain disease states corresponding to atrial fibrillation, dilated cardiomyopathy, and advanced heart failure, automatically identifying subpopulations in high-risk disease trajectories and providing a roadmap of disease state signatures as potential intervention points to mitigate progression.

In contrast to top-down HF classifications based on limited observations such as functional scores and lab values, such data-driven approaches have the potential to more accurately reflect the disease manifestation and progression patterns of HF patients in the real world. Together, these results demonstrate an approach to generate a hypothesis-free, “at a glance” understanding of large HF populations that captures the diversity of clinical presentations and progression and can be computed on large datasets at scale, which represents a step toward building a more comprehensive *heart failure phenome* for understanding the disease manifestation of real-world HF patients. This approach utilizes routinely collected EHR data that reflect the population and practices of a specific healthcare center. In particular, the utilization of NLP-based classification of disease and symptom-related concepts in unstructured clinical notes captures the 360-degree physician’s view of patient problems, providing a more complete and nuanced picture of disease severity and manifestation without the need for expensive, time-consuming data abstraction. Such an approach has the potential to deliver real-world insights tuned to the particular needs of clinicians and their patients at the point of care. Additionally, breaking down the HF disease landscape into clinically similar subgroups in time allows for the quantification of risk for various clinical events, which can be used to power alternative risk-enrichment strategies for RCTs that better reflect disease manifestation in the real-world.

## Results

### Clustering longitudinal EHRs into data-driven HF disease states

To build a patient-centric, data-driven understanding of HF, we utilized a clustering approach to infer HF *disease states* based on complaints mentioned in a de-identified, longitudinal EHR dataset. Clinical notes were chosen as the data source for discovering HF disease states, since these unstructured narratives contain detailed information about a patient’s *complaints* (diagnoses, comorbidities, disease severity, symptoms, findings, etc.) that are frequently missing from structured data elements that are used for administrative and financial purposes^35,36^. By grouping discrete time windows across many HF patient timelines based on the similarity of complaints, we can discover the dominant patterns of disease manifestation over time in a large, real-world HF population.

**Figure 1:**
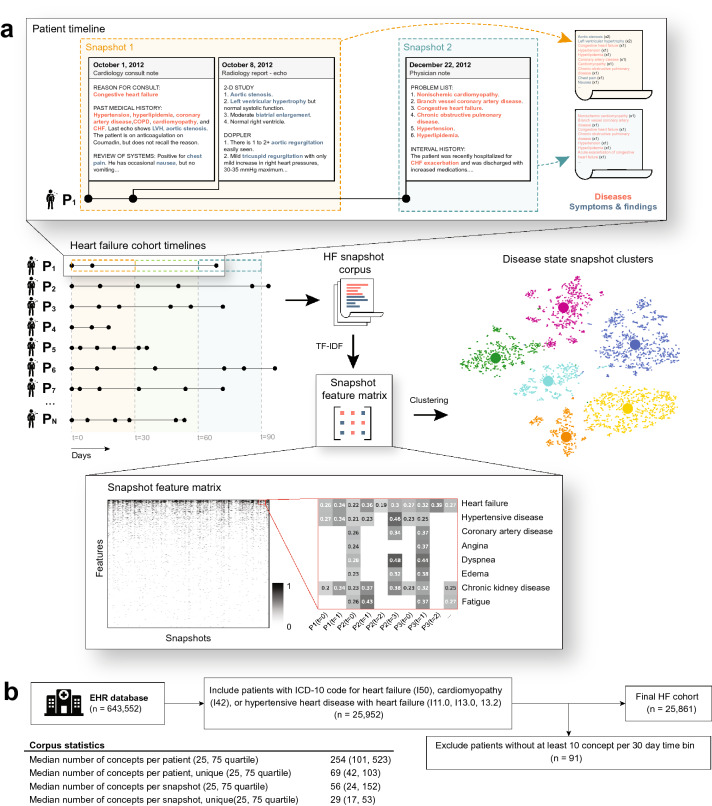
Workflow diagram for converting HF patient EHRs to disease state snapshot clusters. (**a**) Natural language processing was used to extract complaints from all the unstructured notes in each patient’s EHR. Complaints were aggregated within 30 day time bins (clinical state “snapshots”). The snapshots of the entire HF cohort were used as input to a clustering algorithm, which finds groups of similar patient snapshots and produces disease state clusters. (**b**) Inclusion/exclusion criteria and corpus statistics for the final HF cohort.

Analysis was carried out on an EHR dataset from Western Russia^39^. First, a cohort of HF patients was identified (Fig. 1b) and the HF patient trajectories (EHRs) were segmented into 30-day time windows representing the sequence of clinical periods in the longitudinal disease history across the HF cohort. We chose 30 days to represent a clinically relevant time resolution, since a HF patient’s disease state can change significantly month-to-month. The data snapshot for each clinical period consisted of free-text patient *complaints*, which were extracted using a Russian-language clinical named entity recognition (NER) system^36,39^ from the totality of the unstructured notes in patient EHRs. Complaints detected by the NLP system with negative polarity were removed from analysis (e.g., “No evidence of heart failure”). NLP-derived complaint mentions were aggregated within 30-day time bins to create monthlong *snapshots* of the problems experienced by each HF patient (Fig. 1a). The HF cohort included patients with an ICD-10 code for heart failure (I50, I11.0, I13.0, I13.2) or cardiomyopathy (I42). Patients that had less than 10 complaint mentions in any of their snapshots were excluded, resulting in a final study population of 25,861 patients (Fig. 1b). Descriptive statistics for the final study cohort is provided in Table 1.

**Table 1:** Baseline characteristics of the heart failure cohort.

**Database characteristics**	
Patients in cohort	25,861 (100%)
Unique patients with Hypertensive heart disease with heart failure ICD-10 (I11.0, I13.0, I13.2)	3,493 (13.51%)
Unique patients with Cardiomyopathy ICD-10 (I42)	6,754 (26.12%)
Unique patients with Heart failure ICD-10 (I50)	20,500 (79.27%)
**Patient co-morbidities**	
Congestive heart failure	25,793 (99.74%)
Hypertension	21,885 (84.63%)
Ischemic heart disease	21,323 (82.45%)
Cardiac valve disease	19,934 (77.08%)
Angina pectoris	15,307 (59.19%)
Cerebral ischemia	14,626 (56.56%)
Hyperlipidemia	14,123 (54.61%)
Myocardial infarction	13,055 (50.48%)
Obesity	9,337 (36.10%)
Cardiomyopathy	8,984 (34.74%)
Atrial fibrillation and flutter	8,861 (34.26%)
Chronic obstructive pulmonary disease	5,853 (22.63%)
Type 2 diabetes	5,740 (22.20%)
Chronic kidney disease	3,249 (12.56%)
**Patient characteristics**	
N females	11,258 (43.53%)
Age of males (years), median (25, 75 quartile)	59 (48, 67)
Age of females (years), median (25, 75 quartile)	63 (48, 72)
Timeline length (months), median (25, 75 quartile)	4 (1, 28)
BMI, median (25, 75 quartile)	27.34 (23.46, 31.23)

The snapshots for each patient in the HF cohort were vectorized using term frequency-inverse document frequency (TF-IDF)^40^. We then defined HF *disease states* by grouping aggregated complaint snapshot vectors into clusters using K-means clustering^41^. The resultant clusters contain monthlong snapshots of HF patient timelines grouped by similar patterns of complaints, symptoms, and comorbidities, which can then be interpreted as *HF disease states*. After clustering, each snapshot data point is labeled as belonging to a single disease state via cluster assignment. Clustering for $K\in [\mathrm{2,3},...,30]$ revealed stable clusters for $K=[\mathrm{2,3},4,\mathrm{5,7},\mathrm{11,13,23}]$ via cluster bootstrapping^42^ (values of *K* marked with green corridors in Supplementary Fig. S1), which can be understood as the hierarchy of reproducible, data-driven HF disease states.

### Characterizing complaints-driven HF disease states

**Figure 2:**
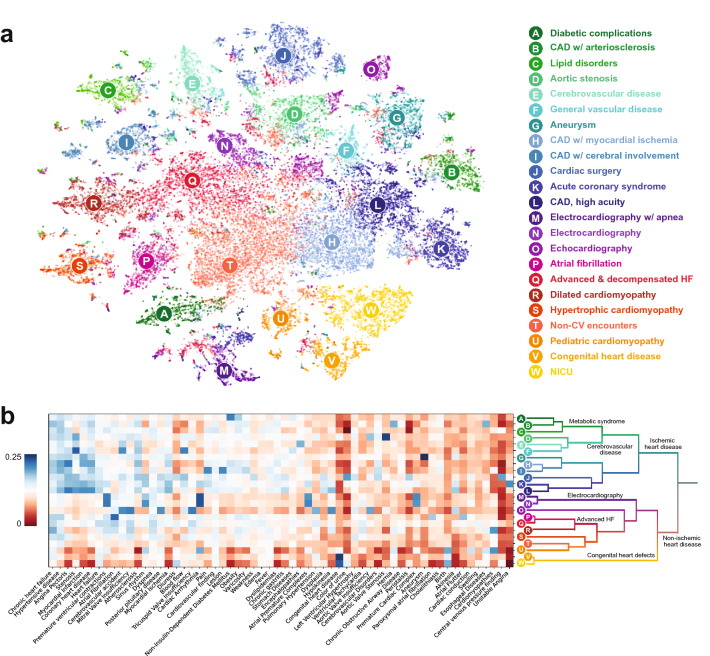
(**a**) Exemplary 2D visualization of the relative distances between all clinical snapshots EHRs in the heart failure cohort using t-SNE. Colors show cluster assignment using K-means clustering (K = 23). (**b**) Visualization of cluster centroids (K = 23) for a subset of complaint features (x-axis). Centroids are sorted by hierarchical clustering and reflect the similarity phenotypes at different values of K. Branches are labeled using a clinical interpretation of the hierarchical structure of the clusters. Each cluster is shown with an interpretable name defining the heart failure phenotype.

In the following analyses, we focus on building a data-driven picture of HF using 23 disease states ($K=23$), the largest value of K investigated that resulted in stable clusters and thus providing the highest level of granularity into HF. To interpret each of the 23 disease states, a right-sided rank sum test was used to find significantly overrepresented input feature complaints (top ten by p-value in Table 2; full list in Supplementary Table S2). We further characterized each cluster by displaying the most common structured diagnosis codes and visit types occurring within the input snapshots (Table 2). Using this information, a clinically interpretable name was assigned to each disease state. We also visualized the HF dataset using t-Distributed Stochastic Neighbor Embedding (t-SNE)^43^ (Fig. 2a). Each point represents a single HF snapshot; the color indicates the cluster assignment for $K=23$. From this, we can visualize the relative distance between individual snapshots across the HF cohort.

**Table 2:** Cluster characteristics for K = 23.

Cluster name	ICD-10 codes	Encounter type	Significant concepts
Diabetic complications	E08–E13: Diabetes mellitus (66.22%) I20: Angina pectoris (27.56%) I25: Chronic ischemic heart disease (23.26%) I50: Heart failure (21.30%) I10–I16: Hypertensive diseases (16.71%)	Ambulatory (69.95%) Inpatient (50.75%) Surgery/diagnostics (32.36%) Other (15.58%)	Non-Insulin-Dependent Diabetes Mellitus (90.7%); Diabetes Mellitus (77.9%); Diabetic Polyneuropathies (66.1%); Diabetic Nephropathy (48.4%); Insulin-Dependent Diabetes Mellitus (45.2%); Retinal Diseases (30.1%); Kidney Diseases (24.4%); Cataract (24.1%); Hyperglycemia (22.7%); Vascular Diseases (20.9%); Complications of Diabetes Mellitus (20.6%); Endocarditis (20.4%); Chronic Kidney Diseases (20.3%); Glucose level (19.9%); Diabetic Retinopathy (17.1%)
CAD w/ arteriosclerosis	I25: Chronic ischemic heart disease (83.28%)	Ambulatory (95.84%) Surgery/diagnostics (11.38%)	Coronary Arteriosclerosis (81.3%); Chronic heart failure (81.2%); Hernia (47.4%); Esophageal Diseases (44.3%); Interventricular dyssynchrony (14.2%); Splenomegaly (15.4%); Hyperuricemia (6.8%); Cardiac dilatation (3.2%); Coronary heart disease (66.1%); Cardiomegaly (11.4%); Atherosclerosis (43.7%); Nephrosclerosis (4.5%); Chronic Kidney Diseases (6.6%); Dyslipidemias (31.5%); Hypertensive disease (75.2%)
Lipid disorders	I25: Chronic ischemic heart disease (27.78%) I10–I16: Hypertensive diseases (27.55%) I20: Angina pectoris (24.93%) I42: Cardiomyopathy (17.76%)	Ambulatory (99.41%)	Lipid Metabolism Disorders (100.0%); Overweight (45.8%); erythrocyte sedimentation rate result (26.9%); Cardiovascular finding (56.9%); Hiatal Hernia (17.1%); Menopause (14.9%); Hypertensive disease (66.7%); Increase in blood pressure (15.2%); Edema (36.2%); Heart Diseases (13.1%); Pain (37.9%); Dizziness (15.7%); Kidney Diseases (8.2%); Obesity (32.0%); Scheuermann's Disease (5.9%)
Aortic stenosis	I35: Nonrheumatic aortic valve disorders (59.81%) I50: Heart failure (31.48%) I20: Angina pectoris (18.84%) Z00–Z13: Persons encountering health services for examinations (15.86%) I60–I69: Cerebrovascular diseases (13.86%) I05–I09: Chronic rheumatic heart diseases (12.77%) I25: Chronic ischemic heart disease (10.49%)	Ambulatory (72.78%) Surgery/diagnostics (45.52%) Inpatient (45.01%)	Aortic Valve Stenosis (94.8%); Stenosis (75.5%); Aortic Valve Insufficiency (61.2%); Calcinosis (57.2%); Heart Diseases (32.2%); Heart valve disease (26.7%); Heart Neoplasm (26.5%); Aortic valve disorder (22.7%); Bicuspid aortic valve (18.6%); Atherosclerosis of aorta (15.1%); Chronic rheumatic heart disease (14.0%); Mitral Valve Stenosis (12.7%); Aortic valve area (6.1%); Aortic valve calcification (5.1%); Fibrous ring (12.1%)
Cerebrovascular disease	I60–I69: Cerebrovascular diseases (81.39%) 10-I16: Hypertensive diseases (20.79%) I20: Angina pectoris (17.49%) I25: Chronic ischemic heart disease (16.12%) M40–M43: Deforming dorsopathies (10.20%)	Ambulatory (66.21%) Inpatient (54.05%) Other (19.35%) Surgery/diagnostics (18.68%)	Dysarthria (93.9%); Gagging (84.7%); Encephalopathies (82.0%); Corneal Reflexes (64.7%); Nystagmus (55.2%); Pupil reaction to light (49.2%); Dysphonia (47.9%); Deglutition Disorders (46.6%); Dizziness (43.7%); Cerebral Atherosclerosis (42.5%); Muscle Tension (41.8%); Ataxia (32.5%); Headache (32.3%); Diadochokinesia (20.6%); Ischemic stroke (19.8%)
General vascular disease	Z00–Z13: Persons encountering health services for examinations (40.97%) I20: Angina pectoris (38.51%) I25: Chronic ischemic heart disease (32.00%) I60–I69: Cerebrovascular diseases (22.22%) I70–I79: Diseases of arteries, arterioles and capillaries (16.61%) I50: Heart failure (15.56%) I10–I16: Hypertensive diseases (11.15%)	Ambulatory (82.95%) Surgery/diagnostics (46.02%) Inpatient (31.31%)	Stenosis (92.2%); Blood flow (79.9%); Cerebrovascular accident (76.4%); Senile Plaques (69.2%); Plaque (lesion) (65.2%); Decompression Sickness (38.2%); Atrophic (24.0%); Lung consolidation (18.1%); Peak systolic (12.2%); Systemic Scleroderma (4.0%); Atherosclerosis (59.7%); Atrophic Vaginitis (4.2%); Stomach Diseases (38.9%); Carotid Stenosis (4.8%); Superficial ulcer (11.8%)
Aneurysm	I25: Chronic ischemic heart disease (45.60%) I20: Angina pectoris (36.34%) I50: Heart failure (34.73%) I70–I79: Diseases of arteries, arterioles and capillaries (27.89%) Z00-Z13: Persons encountering health services for examinations (11.26%)	Ambulatory (66.80%) Inpatient (54.61%) Surgery/diagnostics (42.43%) Other (11.02%)	Aneurysm (99.7%); Myocardial Infarction (71.4%); Thrombus (31.6%); Left ventricular aneurysm (18.2%); Dissection of aorta (13.1%); Aortic Aneurysm (12.7%); Abdominal Aortic Aneurysm (11.1%); Cardiac dyskinesia (16.3%); Akinesia (38.0%); Atherosclerosis of aorta (13.9%); Aneurysm of ascending aorta (2.0%); Old thrombus (2.3%); Myocardial Ischemia (58.8%); Coronary heart disease (75.1%); Atherosclerosis (51.9%)
CAD w/ myocardial ischemia	I20: Angina pectoris (56.59%) I25: Chronic ischemic heart disease (40.08%) I50: Heart failure (19.05%) I10-I16: Hypertensive diseases (16.50%)	Ambulatory (81.25%) Surgery/diagnostics (30.96%) Inpatient (30.09%)	Hypertensive disease (89.3%); Coronary heart disease (84.0%); Chronic heart failure (83.7%); Angina Pectoris (81.5%); Myocardial Infarction (58.6%); Atherosclerosis (56.7%); Myocardial Ischemia (53.0%); Dyslipidemias (32.0%); Cerebrovascular Disorders (30.4%); Non-Insulin-Dependent Diabetes Mellitus (30.0%); Hyperlipidemia (25.1%); Duodenal Ulcer (13.7%); Encephalopathies (24.2%); Obesity (32.7%); Prostatic Hyperplasia (12.0%)
CAD w/ cerebral involvement	I20: Angina pectoris (31.32%) I25: Chronic ischemic heart disease (30.95%) I10–I16: Hypertensive diseases (29.96%) I42: Cardiomyopathy (16.76%) I50: Heart failure (13.89%)	Ambulatory (93.79%) Surgery/diagnostics (21.93%) Inpatient (19.75%)	Pain (98.5%); Lesion (96.4%); Vertebrobasilar Insufficiency (91.6%); Hypertensive disease (88.7%); Hyperlipidemia (44.6%); Cerebrovascular Disorders (40.3%); Varicosity (39.2%); Cerebral Atherosclerosis (24.5%); Pyelonephritis (22.3%); Cholecystitis (17.8%); Cerebral Arteriosclerosis (3.5%); Coronary heart disease (73.3%); Gastritis (25.3%); Coronary Arteriosclerosis (18.6%); Fever (63.8%)
Cardiac surgery	I50: Heart failure (79.59%) I20: Angina pectoris (70.65%) I60–I69: Cerebrovascular diseases (37.60%) I25: Chronic ischemic heart disease (30.59%) I70–I79: Diseases of arteries, arterioles and capillaries (18.47%) K20–K31: Diseases of esophagus, stomach and duodenum (16.42%) Z00–Z13: Persons encountering health services for examinations (15.37%) I35: Nonrheumatic aortic valve disorders (14.95%)	Inpatient (99.94%) Surgery/diagnostics (78.22%) Ambulatory (31.21%) Other (15.54%)	Blood flow (96.4%); Sinus rhythm (94.3%); Diuresis (92.2%); Color of urine (92.0%); Weakness (90.2%); Peristalsis (89.3%); Central venous pressure finding (86.7%); Atherosclerosis (86.0%); Cardiac index (85.4%); Angina Pectoris (83.1%); Stenosis (83.0%); Hemostatic function (80.2%); Left Ventricular Hypertrophy (75.2%); Cardiac activity (73.3%); Pulmonary artery pressure (70.8%)
Acute coronary syndrome	I50: Heart failure (73.30%) I20: Angina pectoris (70.28%) I21: Acute myocardial infarction (34.69%) I25: Chronic ischemic heart disease (29.39%) K20–K31: Diseases of esophagus, stomach and duodenum (19.47%) E08–E13: Diabetes mellitus (11.86%)	Inpatient (97.70%) Surgery/diagnostics (61.95%) Ambulatory (35.55%) Other (22.33%)	Coronary heart disease (96.0%); Myocardial Ischemia (92.7%); Sinus rhythm (87.6%); Stenosis (80.4%); Blood flow (78.5%); Myocardial Infarction (77.6%); Cardiac Arrhythmia (76.6%); Pain (75.7%); Atrial Premature Complexes (73.8%); Unstable angina (62.6%); Systemic arterial pressure (61.9%); Acute Coronary Syndrome (59.2%); Wakefulness (59.1%); Acute myocardial infarction (56.2%); Akinesia (50.0%)
CAD, high acuity	I20: Angina pectoris (76.61%) I50: Heart failure (53.38%) I25: Chronic ischemic heart disease (47.44%) K20–K31: Diseases of esophagus, stomach and duodenum (24.12%) Z00–Z13: Persons encountering health services for examinations (13.39%) I70-I79: Diseases of arteries, arterioles and capillaries (11.63%)	Surgery/diagnostics (69.74%) Inpatient (69.03%) Ambulatory (58.10%)	Coronary heart disease (95.8%); Hypertensive disease (90.8%); Angina Pectoris (88.4%); Myocardial Infarction (77.4%); Myocardial Ischemia (76.3%); Stenosis (73.8%); Heart failure (67.2%); Hypokinesia (58.8%); Atherosclerosis (54.7%); Chronic gastritis (50.6%); Akinesia (49.5%); Systemic arterial pressure (48.1%); Exercise-induced angina (34.3%); Chronic myocardial ischemia (33.0%); Dysplasia (32.8%)
Electrocardiography w/ apnea	I25: Chronic ischemic heart disease (19.21%) I42: Cardiomyopathy (19.12%) I20: Angina pectoris (16.57%) I50: Heart failure (14.47%) I10–I16: Hypertensive diseases (13.87%) Z00–Z13: Persons encountering health services for examinations (13.83%)	Ambulatory (85.49%) Inpatient (22.59%) Surgery/diagnostics (13.78%)	Slow shallow breathing (100.0%); Apnea (100.0%); Cardiac Arrhythmia (98.6%); Premature ventricular contractions (84.1%); Sinus rhythm (76.8%); Atrial Premature Complexes (76.4%); Premature Cardiac Complex (75.1%); Supraventricular arrhythmia (49.0%); Ventricular arrhythmia (34.4%); Bradycardia (32.4%); Atrial tachycardia (24.0%); Sinus Arrhythmia (16.7%); Respiratory Insufficiency (17.2%); Tachycardia (28.8%); Decreased systolic arterial pressure (4.1%)
Electrocardiography	I50: Heart failure (21.81%) I42: Cardiomyopathy (17.33%) I10–I16: Hypertensive diseases (15.13%) I20: Angina pectoris (14.80%) I47: Paroxysmal tachycardia (14.60%) I25: Chronic ischemic heart disease (13.39%) I49: Other cardiac arrhythmias (11.62%)	Ambulatory (60.71%) Inpatient (50.59%) Surgery/diagnostics (23.98%) Other (10.95%)	Cardiac Arrhythmia (94.5%); Sinus rhythm (87.3%); Premature ventricular contractions (87.0%); Atrial Premature Complexes (82.5%); Premature Cardiac Complex (69.6%); Wakefulness (60.2%); Ventricular arrhythmia (46.3%); Supraventricular arrhythmia (43.8%); Bradycardia (36.7%); Tachycardia (35.5%); Respiratory Insufficiency (23.4%); Atrial tachycardia (21.8%); Sinus Arrhythmia (19.7%); ST segment (16.1%); Parasystole (14.5%)
Echocardiography	I25: Chronic ischemic heart disease (19.95%) Z00-Z13: Persons encountering health services for examinations (17.94%) I42: Cardiomyopathy (15.79%) I20: Angina pectoris (10.46%) I10–I16: Hypertensive diseases (10.22%)	Ambulatory (95.09%)	Mitral Valve Insufficiency (95.9%); Tricuspid Valve Insufficiency (95.8%); Cerebrovascular accident (95.4%); Pulmonary Valve Insufficiency (78.7%); Diastolic dysfunction (73.8%); Aortic Valve Insufficiency (53.2%); Hypokinesia (53.2%); Pulmonary Hypertension (50.9%); Calcinosis (32.3%); Akinesia (27.8%); Eccentric hypertrophy (24.8%); Muscle Rigidity (24.7%); Concentric hypertrophy (23.8%); Fibrosis (14.4%); Idiopathic pulmonary arterial hypertension (13.7%)
Atrial fibrillation	I48: Atrial fibrillation and flutter (89.80%) I50: Heart failure (14.05%) I10–I16: Hypertensive diseases (10.80%)	Ambulatory (75.78%) Inpatient (45.68%) Surgery/diagnostics (36.71%) Other (11.54%)	Atrial fibrillation and flutter (89.1%); Atrial Fibrillation (87.4%); Paroxysmal atrial fibrillation (43.1%); Atrial Flutter (39.8%); Persistent atrial fibrillation (20.7%); Under local anesthesia (14.7%); Irregular heart beat (13.5%); Ablation frequency (8.6%); Heart beat (18.3%); Palpitations (19.5%); Post-op diagnosis (6.0%); Fibrillation (7.1%); Cardiac rhythm type (10.3%); Chronic heart failure (80.9%); Cardiac conduction (25.9%)
Advanced & decompensated HF	I50: Heart failure (60.17%) I25: Chronic ischemic heart disease (18.14%) I42: Cardiomyopathy (15.30%) I47: Paroxysmal tachycardia (13.04%) I20: Angina pectoris (11.67%) Z00–Z13: Persons encountering health services for examinations (10.65%) I48: Atrial fibrillation and flutter (10.19%)	Inpatient (80.24%) Ambulatory (50.83%) Surgery/diagnostics (29.59%) Other (21.84%)	Dyspnea (88.6%); Mitral Valve Insufficiency (84.5%); Heart failure (84.1%); Tricuspid Valve Insufficiency (81.6%); Edema (73.4%); Atrial Fibrillation (62.3%); Weakness (61.8%); Diuresis (59.4%); Pulmonary Hypertension (55.0%); Peripheral edema (49.4%); Swelling (46.2%); Decompensation (43.8%); Ventricular Tachycardia (39.5%); Effusion (35.3%); Cardiac asthma (33.4%)
Dilated cardiomyopathy	I42: Cardiomyopathy (87.20%) I50: Heart failure (13.08%) I47: Paroxysmal tachycardia (11.27%)	Ambulatory (82.01%) Inpatient (29.23%) Surgery/diagnostics (18.46%) Other (11.08%)	Dilated cardiomyopathy (96.4%); Chronic heart failure (92.9%); Cardiomyopathies (63.0%); Mitral Valve Insufficiency (62.7%); Ventricular Tachycardia (29.0%); Cardiomegaly (21.6%); Myocarditis (20.2%); Paroxysmal ventricular tachycardia (18.0%); Esophageal Diseases (28.3%); Hernia (30.3%); Dyspnea (61.9%); Interventricular dyssynchrony (8.5%); Hyperuricemia (6.3%); Pulmonary Thromboembolisms (9.3%); Tricuspid Valve Insufficiency (46.5%)
Hypertrophic cardiomyopathy	I42: Cardiomyopathy (88.74%)	Ambulatory (75.90%) Inpatient (31.55%) Surgery/diagnostics (23.54%)	Hypertrophic Cardiomyopathy (100.0%); Hypertrophy (31.6%); Hypertrophic cardiomyopathy without obstruction (17.1%); Dynamic obstruction (7.4%); Asymmetric hypertrophy (6.4%); Primary Cardiomyopathies (6.3%); Left Ventricular Hypertrophy (47.2%); QRS complex feature (2.8%); Subaortic stenosis (3.9%); Heart murmur (18.8%); Syncope (10.2%); Sudden Cardiac Death (4.8%); Systolic Murmurs (16.8%); Diastolic dysfunction (29.2%); Blood flow (42.0%)
Non-CV encounters	I10–I16: Hypertensive diseases (23.11%) I42: Cardiomyopathy (17.16%) I25: Chronic ischemic heart disease (10.72%) I50: Heart failure (10.18%)	Ambulatory (78.54%) Inpatient (26.98%) Surgery/diagnostics (15.15%)	Pregnancy (18.1%); Gynecological history (14.1%); Splenomegaly (11.4%); Exanthema (9.4%); Arthralgia (7.7%); Bleeding tendency (6.6%); Joint swelling (3.5%); Morning stiffness—joint (2.9%); Osteoporosis (5.1%); erythrocyte sedimentation rate result (17.4%); Toxic diffuse goiter (2.8%); Unspecified Abortion (10.0%); Menopause (14.0%); Mean Corpuscular Volume (3.4%); Autoimmune thyroiditis (12.3%)
Pediatric cardiomyopathy	I50: Heart failure (55.06%) I42: Cardiomyopathy (53.81%) I47: Paroxysmal tachycardia (19.33%) I51: Complications and ill-defined descriptions of heart disease (18.39%) I49: Other cardiac arrhythmias (16.19%) Q20–Q28: Congenital malformations of the circulatory system (16.14%) I44: Atrioventricular and left bundle-branch block (14.45%)	Inpatient (81.07%) Surgery/diagnostics (58.69%) Ambulatory (14.25%) Other (12.11%)	Cardiac Arrhythmia (85.8%); Cardiomyopathies (82.5%); Sinus rhythm (76.4%); Myocarditis (72.8%); Birth (70.3%); Pregnancy (66.8%); Cardiac conduction (63.1%); Microalbuminuria (50.6%); Pericarditis (50.4%); Childbirth (49.8%); Wakefulness (48.3%); Viral respiratory infection (47.9%); Myocardial dysfunction (45.1%); Respiration Disorders (44.8%); Systolic Murmurs (44.1%)
Congenital heart disease	Q20–Q28: Congenital malformations of the circulatory system (83.54%) I50: Heart failure (46.99%) Z00–Z13: Persons encountering health services for examinations (10.75%)	Ambulatory (54.08%) Inpatient (53.72%) Surgery/diagnostics (49.75%)	Atrial Septal Defects (100.0%); Congenital heart disease (90.8%); Congenital Abnormality (51.7%); Congenital Heart Defects (43.1%); Air Embolism (17.3%); Respiration Disorders (14.1%); Fluid overload (10.0%); Birth (32.5%); Right Ventricular Hypertrophy (10.0%); Pregnancy (34.9%); Cardiac activity (19.2%); Under local anesthesia (9.8%); Systolic Murmurs (23.8%); Childbirth (18.2%); Hereditary Diseases (8.9%)
NICU	Q20–Q28: Congenital malformations of the circulatory system (94.94%) I50: Heart failure (75.42%) G96: Other disorders of central nervous system (21.33%) Z00–Z13: Persons encountering health services for examinations (17.90%) G93: Other disorders of brain (15.47%) P91: Other disturbances of cerebral status of newborn (10.46%)	Inpatient (86.01%) Surgery/diagnostics (55.26%) Ambulatory (16.20%) Newborn/obstetrics (10.65%)	Congenital heart disease (95.0%); Congenital Abnormality (77.3%); Birth (72.1%); Diuresis (67.0%); Ventricular Septal Defects (60.5%); Pregnancy (59.1%); Congenital Heart Defects (58.3%); Systolic Murmurs (57.0%); Childbirth (47.4%); Wheezing (46.0%); Atrial Septal Defects (42.5%); Color of urine (37.7%); Surgical wound (32.1%); Urination (31.0%); Cyanosis (30.7%)

Top ten most significant concepts for each phenotype, ranked by p-value (smallest to largest). Significance was determined using a one-sided (greater) sum of ranks test with Bonferroni correction testing the null hypothesis that the distribution of values of TF-IDF features for a medical entity in cluster i are drawn from the same distribution as the same entity in all other clusters. The “ICD-10” column shows the six most frequent ICD-10 codes and/or groups of codes with more than 5% incidence within the cluster. The “Encounter type” column shows the types of encounters that comprise the snapshots within each cluster.

Examining Table 2 and Fig. 2a, it is apparent that the snapshots of HF patients are grouped into clusters with shared clinical characteristics. Qualitatively, we observe that some clusters appear more similar to each other than others. For example, clusters for *Ischemic heart disease*, *Acute coronary syndrome*, and *CAD & HTN, male* are grouped closely together. To quantify and visualize the natural hierarchy of heart failure disease states within the cohort, we constructed a dendrogram for $K=23$ using hierarchical clustering (Fig. 2b, right). The stable clusters used in constructing the dendrogram were $K=[\mathrm{2,3},\mathrm{4,5},\mathrm{7,11,13,23}]$ (marked with green corridors in Supplementary Fig. S1). All snapshots are aggregated at the right side of the dendrogram; each successive branch point shows the value of *K* at which a cluster splits into two smaller clusters. Branch points further to the left on the dendrogram represent clusters that are more similar to each other as quantified by their Jaccard index (see Supplementary Methods). The feature values of fifty common complaint features are shown for $K=23$, sorted by the order in the dendrogram (Fig. 2b, left).

**Figure 3:**
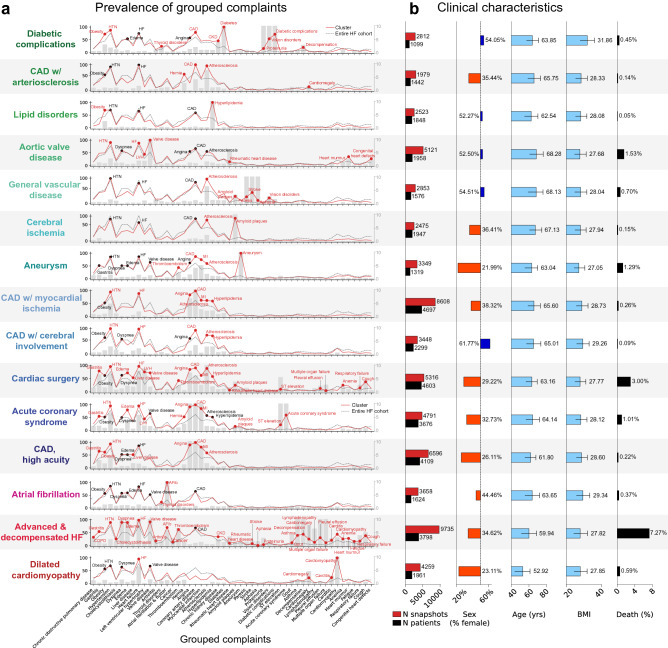
(**a**) The prevalence of various grouped complaints per cluster disease state. Traces in red show the prevalence (%) of the grouped complaints within the cluster, while the dotted gray line shows the prevalence in the entire cohort. Gray bars indicate the odds ratio of the grouped complaint occurring within the cluster. Complaint groups with an odds ratio > 2 within the disease state cluster are marked with a red circle and the text of the complaint. Complaint groups with an odds ratio < 2 but a prevalence > 50% within the disease state cluster are shown with a black circle and the text of the complaint. (**b**) Clinical characteristics of disease state clusters.

Finally, to further characterize HF disease states, we aggregated input feature complaints into *grouped complaint phenotypes*. For example, the phenotype *Myocardial infarction* aggregates the more specific complaints “Myocardial infarction,” “Acute myocardial infarction”, “Subendocardial myocardial infarction”, “History of myocardial infarction”, “Post-myocardial infarction syndrome”, and “Recent myocardial infarction” (Supplementary Table S3). Doing so allows us to visualize the prevalence and specificity of meaningful clinical phenotypes for each disease state (selected clusters shown in Fig. 3a). We also characterized these disease states in terms of number of patients/snapshots falling within the cluster, sex distribution, age, body mass index (BMI), and in-hospital mortality (Fig. 3b).

We show that a hypothesis-free, high dimensional approach using unstructured data can successfully segment temporal snapshots of HF into meaningful groups without the need for any time-consuming, top-down data structuring approaches. In the next section, a detailed clinical interpretation of HF disease states is presented based on these results.

### Clinical interpretation of HF disease states

From Fig. 2b, we observe that HF disease states can be interpreted hierarchically. We use this hierarchy and disease state characteristics as a framework to provide a clinical description of each HF disease state (Table 3). The first major split in HF occurs between ischemic and non-ischemic heart disease. Ischemic heart disease then splits into subgroups corresponding to disease states dominated by metabolic, vascular, and heart valve disease (*Diabetic complications*, *CAD with arteriosclerosis*, *Lipid disorders*, *Aortic stenosis*, *Cerebrovascular disease*, and *General vascular disease*) and those with acute coronary syndrome, aneurysm, and cardiac surgery (*Aneurysm*, *CAD with myocardial ischemia*, *CAD with cerebral involvement*, *Cardiac surgery*, *Acute coronary syndrome*, and *CAD, high acuity*).

**Table 3:** Clinical description of heart failure disease states.

Disease state	Description
Diabetic complications	Advanced diabetic complications (diabetic kidney disease, neuropathy, retinopathy, diabetic foot) with high rates of obesity, hypertension, and coronary artery disease
CAD w/arteriosclerosis	Coronary artery disease and associated comorbidities (hypertension, hyperlipidemia, diabetes, obesity, COPD, kidney disease, gastric complaints) in a primarily outpatient setting. Higher rates of atrial fibrillation and cardiomegaly
Lipid disorders	Patients with hyperlipidemia, obesity, and hypertension treated in a primarily outpatient setting
Aortic stenosis	Aortic stenosis and associated problems and symptoms, including aortic valve insufficiency, aortic valve calcification, bicuspid aortic valve, endocarditis, and congenital heart disease
Cerebrovascular disease	Patients with several forms of cerebrovascular disease, including cerebral atherosclerosis, carotid stenosis, and stroke (and common sequelae and complications following stroke). Also common are symptoms of cerebrovascular disease such as vertigo and amnesia, among others
General vascular disease	Vascular disease, including cerebrovascular and peripheral vascular disease
Aneurysm	Predominantly male (82%) patients experiencing an aneurysm. Ischemic heart disease and associated problems such as myocardial infarction and angina are common in this group
CAD w/ myocardial ischemia	Coronary artery disease and associated comorbidities (hypertension, hyperlipidemia, diabetes, obesity, COPD, kidney disease, gastric complaints) in a primarily outpatient setting. Higher rates of male sex (62%), angina, and myocardial infarction
CAD w/ cerebral involvement	Coronary artery disease and associated comorbidities (hypertension, hyperlipidemia, diabetes, obesity, COPD, kidney disease, gastric complaints) in a primarily outpatient setting. Higher rates of female sex (62%) and vertebrobasilar insufficiency
Cardiac surgery	Cardiac surgery, primarily coronary artery bypass grafting and valve repair
Acute coronary syndrome	Acute coronary syndrome, including acute myocardial infarction and unstable angina
CAD, high acuity	Coronary artery disease in a primarily inpatient setting. Common comorbidities include hypertension, diabetes, COPD, and stomach disease
Electrocardiography w/ apnea	Electrocardiography diagnostic findings, including cardiac arrhythmia, ventricular arrhythmia, premature ventricular contractions, atrioventricular block, atrial fibrillation, bradycardia, and tachycardia. Also includes respiratory findings commonly found during sleep studies
Electrocardiography	Electrocardiography diagnostic findings, including cardiac arrhythmia, ventricular arrhythmia, premature ventricular contractions, atrioventricular block, atrial fibrillation, bradycardia, and tachycardia
Echocardiography	Echocardiography diagnostic findings, including valve insufficiency, diastolic dysfunction, left ventricular hypertrophy, stenosis, and cardiomegaly
Atrial fibrillation	Atrial fibrillation and flutter, with significantly enriched rates of obesity, hypertension, and thyroid disorders
Advanced & decompensated HF	Late-stage heart failure and associated symptoms, including edema, dyspnea, and cardiac asthma. Patients experience high rates of acute decompensation, in-hospital mortality, and acute problems such as multiple organ failure, cardiac arrest, respiratory failure, and acute kidney failure. Patients typically have several comorbidities and concomitant problems, including atrial fibrillation, COPD, anemia, and dilated cardiomyopathy
Dilated cardiomyopathy	Dilated cardiomyopathy with well-known symptoms, such as dyspnea, cardiac asthma, edema, valve insufficiency, arrhythmia, clotting events, and pulmonary hypertension. This group is predominantly male and has frequent documentation of alcohol abuse, chronic liver disease, and COPD, supporting evidence of alcoholic cardiomyopathy
Hypertrophic cardiomyopathy	Hypertrophic cardiomyopathy and commonly reported symptoms, including syncope, heart murmur, and mitral valve insufficiency
Non-CV encounters	Group with lower rates of cardiovascular complaints but higher rates of other problems such as autoimmune disease (e.g., toxic diffuse goiter, autoimmune thyroiditis, Graves disease, lupus, and rheumatoid arthritis), blood cancers (e.g., multiple myeloma, lymphoma, and leukemia), and gynecological and obstetric findings (etc., fibroid tumor, pregnancy, menopause)
Pediatric cardiomyopathy	Primarily pediatric population with cardiomyopathy related to myocarditis and pericarditis

The first split within the non-ischemic heart disease branch contains infants and children with congenital heart disease (*Congenital heart defects* and *Neonatal intensive care* (*NICU*)). The next branch contains disease states dominated by cardiac diagnostic modalities (*Echocardiography*, *Electrocardiography*, and *Electrocardiography with apnea*). Finally, at higher values of K, the non-ischemic branch culminates in two further subgroups; the first contains patients with various types of cardiomyopathies (*Hypertrophic cardiomyopathy* and *Pediatric cardiomyopathy*) and various non-cardiac complaints (*Non-cardiovascular* (*CV*) *encounters*), while the second contains patients with advanced heart failure and high-risk comorbidities (*Atrial fibrillation*, *Dilated cardiomyopathy*, and *Advanced & decompensated HF*).

Table 3 provides a brief clinical description of each disease state (based on information in Table 2, Fig. 3, Supplementary Table S4). A more in-depth description of each disease state can be found in the supplementary appendix.

### Quantifying HF patients at risk

Understanding risk in patient populations is central to healthcare, from therapy development to clinical decision making. HF patients in particular are at significantly higher risk of morbidity and mortality^4^. From the previous section, we can observe that the HF disease states represent differential levels of disease severity, and that there are complaints associated with certain states that correspond to poor outcomes (e.g., “decompensation” in *Advanced & decompensated HF*). To quantify the risk within the HF cohort, we defined clinical events of interest, including patient encounter type (hospitalization, ICU admission), in-hospital mortality, acute conditions (ischemic stroke, acute kidney injury, decompensation, pulmonary embolism, aneurysm), and procedures (CABG, angioplasty, septal defect correction, aneurysm repair, valve repair & prosthesis, pacemaker implantation, and heart transplant). We then labeled each data point in the dataset with binary labels that indicate whether or not each clinical event occurred within the 30-day snapshot window. Additionally, to quantify the future risk of an event, we labeled snapshots that occurred *before* snapshots containing the event.

**Figure 4:**
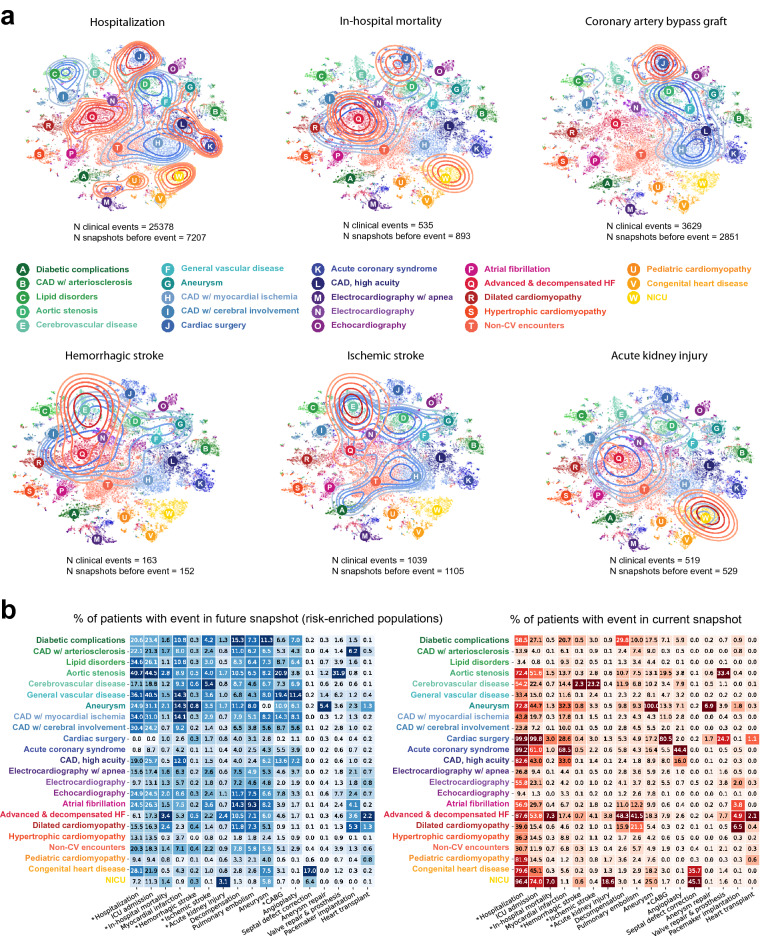
Current and future risk enrichment for clinical events in HF disease states. (**a**) Kernel density estimate of clinical events overlaid over HF disease states for six events: hospitalization, in-hospital mortality, and coronary artery bypass graft, hemorrhagic stroke, ischemic stroke, and acute kidney injury. Red density shows the density of snapshots in which the clinical event occurs (N clinical events). Blue density shows the density of snapshots before the first occurrence of the event (N snapshots before event). (**b**) Prevalence of clinical events (%) for the patient population in each disease state cluster in (**a**).

Using these labels, the prevalence of clinical events in present and future snapshots can be visualized over the entire HF cohort using kernel density estimation (Fig. 4a). For example, in-hospital mortality is concentrated within *Advanced & decompensated HF*, *NICU*, and a concentrated area of snapshots in *Cardiac surgery*. We also observe that snapshots within the *Aortic valve disease* cluster show relative enrichment for risk for mortality in a future snapshot. Similarly, although CABG occurs primarily within the *Cardiac surgery* disease state, we observe that patients in *CAD, high acuity*, *CAD with myocardial ischemia*, *Cerebral ischemia*, and *Aneurysm* are most at risk for a future CABG. Similarly, although hemorrhagic and ischemic strokes are both concentrated within the *Cerebrovascular disease* state, risk enrichment differs for these two events; for hemorrhagic stroke, patients in *Aneurysm*, *Dilated cardiomyopathy*, *Advanced & decompensated HF*, and *Aortic stenosis* are most at risk, while for ischemic stroke, patients in *Diabetic complications*, *Atrial fibrillation*, *Aortic stenosis*, *General vascular disease* and *Aneurysm* were most at risk.

We also quantified risk of present and future events over all events (Fig. 4b). Using this framework, we can gain insight into the probability of near-term and future events associated with HF subpopulation disease states. For example, in *Lipid disorders* patients, the prevalence of stroke is very low (0.5%) but risk for a future stroke is relatively high (3.0%), uncovering the well-studied association between elevated lipid levels and stroke^4,44–46^ without relying on lipid level measurements as an explicit biomarker. Similarly, we observe that patients in the *Dilated cardiomyopathy* state have a relatively high risk of future in-hospital mortality (2.4%), indicating that patients within this group are likely to suffer adverse outcomes and present a large expense to health systems. This approach to comprehensive risk assessment provides an at-a-glance, high-level understanding of risks of adverse outcomes and patterns of hospital resource consumption across HF disease states which could benefit targeted strategies for risk mitigation in HF subpopulations.

### Building a temporal progression map of HF disease states

**Figure 5:**
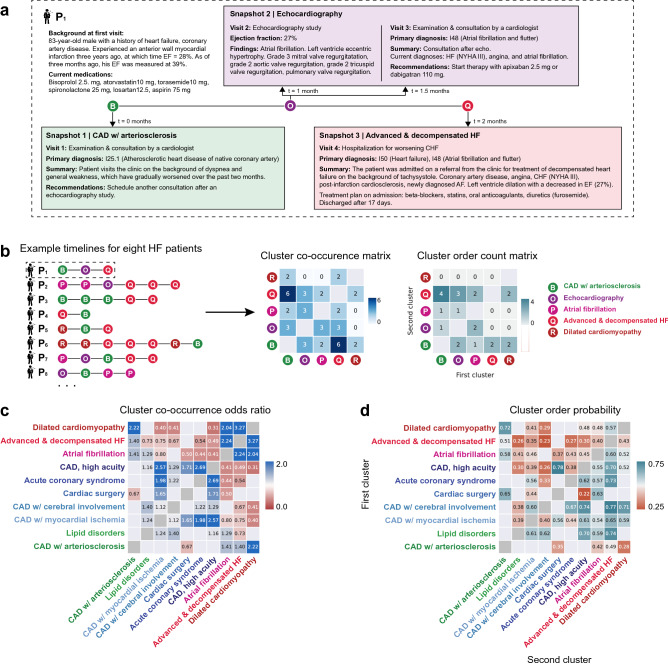
(**a**) Example timeline of a patient in the heart failure cohort. Visits have been aggregated into time bins and a clinical summary of the visits is provided. The disease state (cluster assignment) of each snapshot is shown. (**b**) Schematic illustrating the construction of a cluster co-occurrence matrix and cluster order count matrix using eight patient timelines from the HF cohort. The first timeline (P1) is expanded in (**a**). (**c**) Odds ratio of clusters co-occurring within HF patients for a selected subset of clusters, computed over all patients. Values greater than 1 (blue) indicate a positive association, while values less than 1 (red) indicate a negative association. Odds ratios falling outside of a 95% confidence interval are masked. (**d**) For selected cluster pairs, probability of the first cluster (y-axis) coming before the second cluster (x-axis) in patients in which both clusters co-occur across the entire HF population.

Understanding the patterns of disease progression is vital in chronic, slowly progressing diseases like heart failure. Here, we develop an approach to understand the temporal progression of HF disease states within patient timelines. HF typically manifests as the end stage of another disease process and as a result, HF patients typically experience a large number of comorbidities and clinical states over the course of their disease. To illustrate how multiple disease state clusters manifest during HF progression, Fig. 5a shows an exemplary case study from a patient in the HF cohort. The patient was first referred to a cardiologist at the health system used in our study for worsening symptoms on a background of coronary artery disease and heart failure, at which time the complaints mentioned in the patient’s clinical notes led the disease state to be classified as *CAD with arteriosclerosis*. At the patient’s next snapshot, labeled as *Echocardiography*, they received an echocardiogram, which revealed newly diagnosed atrial fibrillation. Finally, in their last snapshot, the patient was hospitalized with acutely decompensated heart failure and was assigned the state *Advanced & decompensated HF*.

To understand the dominant patterns of disease state progression over time across the entire HF cohort, we employ a twofold approach that first finds significantly co-occurring disease state pairs within patient timelines and then determines the probability of each disease state occurring first or second within a patient timeline. To do so, we count the number of patients in which each disease state pair co-occurs, then compute the odds ratio of the two states co-occurring. A positive and statistically significant odds ratio quantifies the association between pairs of disease states. Next, for each disease state pair ${S}_{i}$ and ${S}_{j}$, we count the number of times that ${S}_{i}$ occurs before ${S}_{j}$ in patients in which both disease states occur. From this, we can compute the probability of ${S}_{i}$ preceding ${S}_{j}$ across the entire HF cohort, revealing the order in which positively associated disease state pairs tend to occur. The process of computing cluster co-occurrence and temporal ordering is shown for eight exemplary HF patient timelines in Fig. 5b. Statistically significant cluster co-occurrence odds ratios and cluster order probabilities for ten disease state clusters are shown in Fig. 5c,d (results for all clusters are shown in Supplementary Figs. S4 & S6).

**Figure 6:**
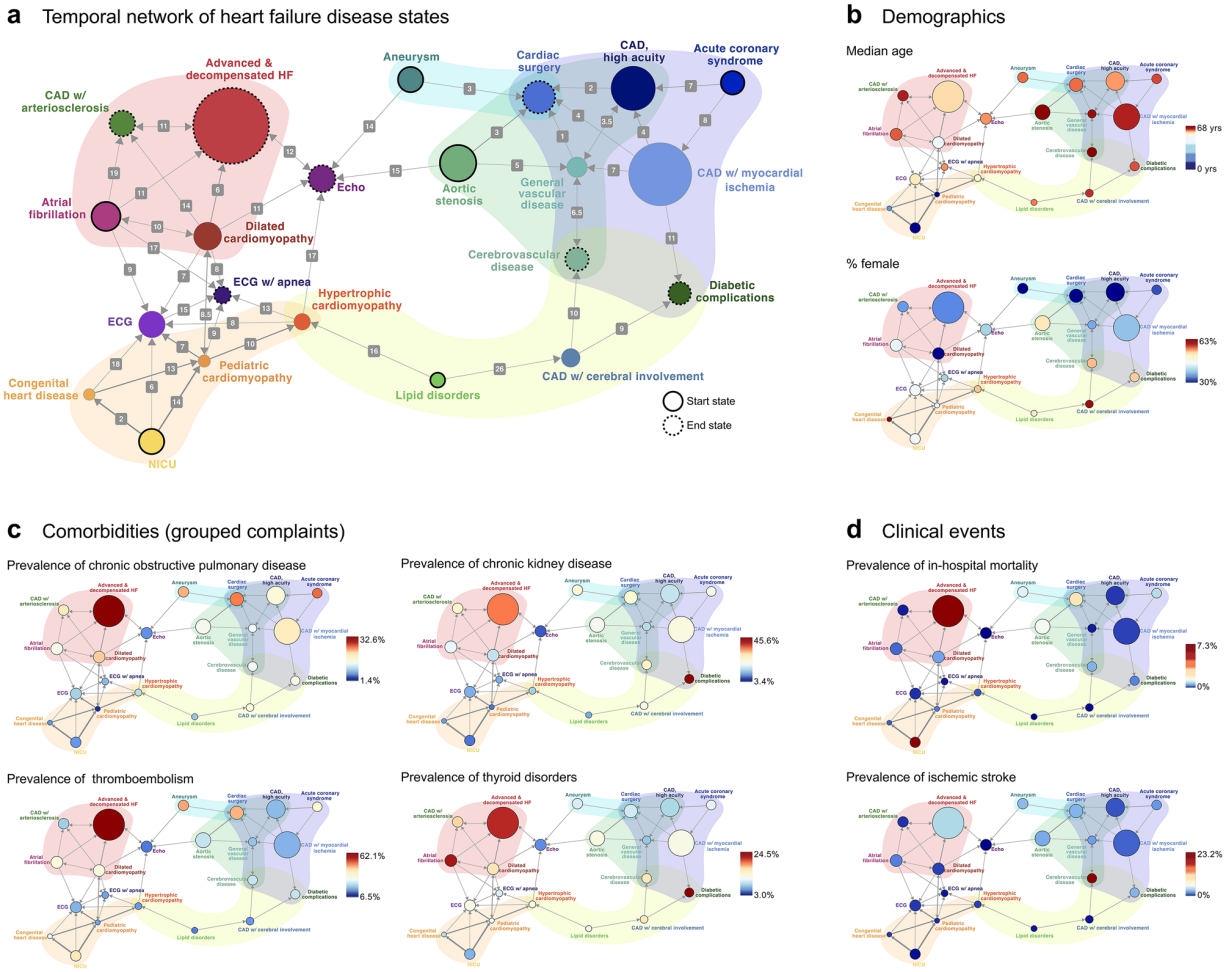
(**a**) Temporal patterns of HF evolution. Network diagram showing connections between positively associated clusters (odds ratio > 1.33). Node size is proportional to the number of snapshots with the corresponding cluster assignment. Arrow thickness is proportional to the odds ratio in Fig. 5c. Arrow direction is derived from Fig. 4b; probabilities in the range [0.45, 0.55] are shown as bidirectional arrows. The median number of 30 days bins (months) for patients to transition between disease states is overlaid on the arrows. When arrows are bidirectional, the mean of the two values is shown. Disease states with only outgoing arrows are as labeled as “start” states, whereas disease states with only incoming arrows are labeled as “end” states. HF disease state subnetworks are shown with transparent overlays. (**b**) The prevalence of demographics (age, sex), (**c**) comorbidities (grouped complaints), and (**d**) clinical events overlaid on the network in (**a**).

Using the odds ratio computed from disease state co-occurrence between all clusters, we can construct a temporal network of HF disease states (Fig. 6a, odds ratio ≥ 1.33). The directionality of network edges reflects the disease state order probability in Fig. 5d, where probabilities in the range [0.45, 0.55] are shown as bidirectional arrows. The median time to transition between two disease states is overlaid on each edge. In this representation, we can observe disease states with only outgoing arrows, which we labeled as “start” states, and disease states with only incoming arrows, which we labeled as “end” states. We also observed a set of partially overlapping HF disease state “subnetworks” that contain both “start” and “end” states and a network of high-frequency state transition pathways, which are shown with transparent overlays. To aid in the interpretation and clinical insights of this network, demographic information (Fig. 6b), grouped complaint phenotypes (Fig. 6c), and clinical events (Fig. 6d) are shown overlaid on the network.

Visualizing demographics, grouped complaint phenotypes, and clinical events on top of this network provides a unique framework for hypothesis-free and scalable insight generation within a health system. Some example subnetworks are highlighted below.

#### Juvenile HF

The youngest patients are concentrated in the subnetwork containing *NICU*, *Congenital heart disease*, and *Pediatric cardiomyopathy* (Fig. 6b, orange overlay). The disease state *NICU* serves as an entry point for HF for infants with congenital heart disease; those who survive the ICU (Fig. 6d) transition to the more stable states of *Congenital heart disease* and *Pediatric cardiomyopathy*.

#### Coronary artery disease, male-dominated

Another subnetwork we observed contains disease states pertaining to ischemic heart disease (Fig. 6a, blue overlay). In this subnetwork, *Acute coronary syndrome* serves as an entry point into further disease states, which supports the understanding of myocardial infarction as a driver of HF^4^. Within this male-dominated subnetwork (Fig. 6b), patients then progress to disease states *CAD with myocardial ischemia* and *CAD, high acuity*. Because they have HF stemming from an ischemic etiology, patients within this subnetwork frequently reach advanced disease end states of *Cardiac surgery* (CABG), *Cerebrovascular disease*, and *Diabetic complications*.

#### Coronary artery disease, female-dominated

In this subnetwork, we observe that *Lipid disorders*, which contains obese patients with hyperlipidemia and hypertension, is also an entry point to HF (Fig. 6a, yellow overlay). This state is commonly followed by *CAD with cerebral involvement*, which contains similar complaints as *Lipid disorders* but also contains additional comorbidities indicative of a more advanced disease state, (e.g., higher rates of atherosclerosis and angina, Fig. 3a). These patients then commonly progress to *Diabetic complications* and *Cerebrovascular disease*. Within the HF network, this subnetwork exhibits the highest prevalence of females (Fig. 6b), corroborating findings of distinct and separate progression patterns for males and females with HF^47^.

#### High-risk HF

We observe another subnetwork (Fig. 6a, red overlay) in which patients with snapshots in the *Atrial fibrillation*, *Dilated cardiomyopathy*, and *CAD with arteriosclerosis* clusters are likely to also experience *Advanced & decompensated HF*, the end state for this subnetwork and the state with the highest adult morality (Fig. 6d) and rates of other indicators of poor outcomes, including thromboembolism (Fig. 6d), respiratory failure, and decompensation (Fig. 3a). Although *CAD with arteriosclerosis* has complaints that are similar to the other disease states of ischemic heart disease, it also contains several complaints that distinguish it from the other groups, including higher rates of cardiomegaly (Fig. 3a) and thyroid disorders (Fig. 6c), which may point to potential factors for differential risk identification within HF subpopulations. In fact, thyroid disorders have a uniformly high prevalence in the subnetwork with advanced HF and within the *Diabetic complications* state, identifying correlates of poor outcomes and potentially identifying patients in need of alternative treatment strategies^48^. Similarly, the prevalence of CKD is highest in *Advanced & decompensated HF* and *Diabetic complications* (Fig. 6c), two of the most advanced disease states within the HF network, matching previous findings of CKD as a risk factor for poor outcomes^47^.

Taken together, these results highlight the typical progression patterns of HF disease states within a single health system. Importantly, we show that there are several subnetworks of disease states, which contain patients with similar clinical manifestations and progression pathways of HF.

## Discussion

Understanding real-world disease manifestation and progression is a crucial step in developing and implementing effective interventions^49^. Historically this has been challenging in practice, especially in multifaceted syndromes such as HF where patients are elderly, highly complex, and have a range of lifestyle factors that vary widely across populations and play a large role in disease development and progression^4,50^. Disease-specific guidelines derived from results of RCTs with narrow inclusion/exclusion criteria, enrollment biases, and highly protocolized care may not be generalizable to large segments of the population across diverse geographies, sociodemographic categories, or health systems^49^. Additionally, such guidelines typically rely heavily on biomarkers and functional classifications, which may not reflect the nuance of disease presentation and severity that may be necessary to drive more targeted intervention strategies^10,11^. As a result, pharmaceutical companies and healthcare providers do not have an accurate and complete map of the HF disease landscape that is inclusive of all manifestations, progression patterns, and treatment responses across the full spectrum of HF patients that can be used to guide development of better interventions and management strategies. Generating practice-based evidence from RWD has emerged as a potential solution to these problems^20^, but challenges in data quality and methodological limitations to take noisiness into account have limited the applications of RWD^51–53^. Much research has focused on supervised predictive models that use limited variables or curated data for specific use cases^22–26^, but these approaches do not offer a more top-down view of disease that can be used in higher-level decision making.

In this study, we present an alternative approach to understanding the real-world manifestation and progression of HF by using a data-driven methodology for identifying HF disease states and common trajectories. In contrast to top-down approaches that use predefined criteria to classify HF disease states, the unsupervised, hypothesis-free approach presented in this study requires no high-level data structuring or top-down definitions. Instead, we utilized free-text mentions of complaints extracted from clinical notes, which contain the rich descriptors of patient disease that are not found elsewhere in RCTs or other secondary use datasets. The resultant HF disease states are clinically meaningful in terms of HF etiology, comorbid conditions, symptoms, disease severity, and care utilization patterns, and have the potential to provide a richer and more complete picture of patient disease states at both an individual and population level compared to top-down classifications. Characterizing these HF states over time reveals a more differentiated picture of HF presentation and evolution dynamics across a large population, which begins to characterize a comprehensive HF phenome that can be computed on large datasets at scale to capture the patterns of clinical presentation of HF patients based on real-world clinical practice.

We demonstrate the utility of the HF phenome in a large HF population in Western Russia. By revealing how patients with similar disease states typically progress into more severe disease states over time, our approach allows us to identify differential risk patterns for future undesirable events in high-risk HF disease states. For example, by identifying the disease states in which thromboembolisms (Fig. 6c) or ischemic strokes (Fig. 6d) are most prevalent, the patients on the pathways that lead to these higher-risk states can be specifically targeted for early intervention. We also observe HF disease states (for example, those corresponding to outpatient CAD) that appear very similar in terms of complaints and patient characteristics but that have very different risk profiles and progression pathways, which may not be differentiated using a limited set of top-down criteria (such as trial inclusion/exclusion criteria). This intriguing finding that similar-looking patient subgroups can experience dramatically different progression pathways further supports the assertion that current classification schemes do not adequately represent real world HF disease trajectories and warrants further research into the factors leading to disparate outcomes across HF subpopulations. This breakdown of the HF disease landscape into clinically-similar disease states over time allows for the quantification of risk of various endpoints within more precisely defined subpopulations, which can help providers decide which populations to focus on within resource-constrained care settings. At the same time, for the pharmaceutical industry there is also potential to enable risk-enrichment strategies in RCTs that more reliably reflect disease progression in the real world. EHR-derived progression phenotypes could also provide evidence for alternative clinical endpoints that truly reflect the disease burden and medical need under standard of care conditions. This can be particularly valuable when population-specific guidance from RCTs are limited, unreliable, or unavailable.

An additional advantage of the data-driven approach employed in this work is that the insights into HF manifestation and progression are generated in a scalable manner on routinely collected EHR data and can thus serve as the basis for a highly tailored and localized landscape of disease that reflects the population and practices specific to targeted subpopulations, including those found in individual healthcare systems. Using a single unified approach, we find that we are able to replicate many well-established etiologies, risk factors, and progression patterns of HF. At the same time, our approach also surfaces patterns localized to the specific study population that may not be apparent via top-down methods. For example, in the Russian EHR dataset used for this study, we found a high prevalence of younger males with alcoholic cardiomyopathy^54^ and high prevalence of structural valve changes and complications due to rheumatism^55^, two groups which would likely still be present but likely at much lower numbers in a US dataset. Localized HF landscapes could serve as the foundation for data-driven personalized medicine, with the potential to deliver real-world insights tuned to the particular needs of clinicians and their patients at the point of care. Given that large portions of real-world HF populations are still underrepresented in clinical research, especially older patients, women, minorities^56^, and patients living outside specific geographies that are typically targeted for RCTs, this approach may be of particular value for risk-bearing healthcare entities to better understand outcomes and treatment performance more accurately and completely across diverse populations. Periodic regeneration and assessment of localized HF landscapes within healthcare systems over time could also help providers understand the impact of changes in HF management strategies and the local patient population on outcomes and provide a feedback system for further hypothesis generation and optimization of care programs.

## Limitations and future directions

In this study, we utilize clinical notes from a single health system to generate an unsupervised, hypothesis-free understanding of HF disease states and progression pathways. A potential limitation of our study is that although a large number of HF patients were used in the analysis (N = 25,861), it remains to be determined whether the HF disease states presented in this work will remain stable across larger patient populations and geographies. Additionally, in this work we defined HF disease states using only complaints extracted from clinical narratives in the EHR without using any structured data (e.g., diagnostic codes) or structured or unstructured data corresponding to clinical interventions (e.g., medications, procedures) or measurements (e.g., vitals, labs, diagnostics). Future avenues of research can explore utilizing additional sources of information in the EHR as data sources for HF subtyping, as well as additional healthcare data sources such as administrative data and claims.

The clustering methodology used in this study assigns one disease state per data point. A future direction of study could utilize multi-assignment clustering models, which may be explored in an attempt to explicitly model multimorbidity. Furthermore, we observe patients with similar complaints (i.e., CAD disease states) that ultimately experience very different progression pathways and outcomes; future research could explicitly model longer term disease state trajectories rather than the pairwise approach taken in this work to understand more complex, nuanced relationships between disease progression and outcomes.

We demonstrate the ability to generate a high-level understanding of the real-world manifestation of HF within a health system using only unstructured data. A natural extension of this work would be to further analyze these HF subgroups using interventions, outcomes, and other covariates. For example, future research could analyze how covariates such as age, sex, comorbidities or other complaints, or even traditional biomarkers such as LVEF may correlate with or serve as drivers of disease progression between the HF disease states identified in this study. Additionally, because the methodology developed in this study groups patients together by similarity of disease manifestation at a particular point in time, future research could characterize the treatment response of different HF subgroups, potentially identifying treatments with differential impact on disease progression and critical outcome measures across different stages and etiologies of HF. Such an approach can thus serve as the basis for comparative efficacy studies and potentially serve as a supplement or alternative to other population matching methods such as propensity scoring.

## Methods

### Description of dataset

In this study, we analyzed de-identified secondary healthcare data that was extracted from the EHR system of a national medical research center located in Western Russia^39^. The center provides the full cycle of medical services, including inpatient and outpatient departments, imaging, rehabilitation services, perinatal care (including pediatric intensive care and surgery), and dentistry. Inpatient services are spread across various institutes and departments, and include, among others, internal medicine, functional diagnostics, intensive care units (ICU), including neonatal ICU (NICU), surgery (including cardiovascular, oncology, neurology, robotic surgery, etc.), clinical pharmacology, and chemotherapy. The longitudinal records used in this study were collected over a 10-year time span (2008–2018). Use of de-identified data for research purposes was approved by the institution. The study was verified IRB exempt as non-human subjects research not requiring informed consent according to 45CFR46.104(d) (Solutions IRB). All research was performed in accordance with relevant guidelines and regulations.

A Russian-language clinical NER system^36,39^ was used to extract free-text *complaints* from the totality of the unstructured notes in patient EHRs. The NER system (Droice Flamingo) extracts mentions of clinical concepts from several clinically relevant ontologies included in the Unified Medical Language System^57^ (UMLS) and maps them to a concept unique identifier (CUI), which allows different strings to be matched to the same normalized entity (e.g., “Type 2 diabetes” and “DM2” will both be assigned the same CUI). In this study, we extracted entities in SNOMED CT and represented each entity by its normalized CUI. Within UMLS, each CUI has one or more semantic types. We limited our analysis to entities corresponding to patient complaints (e.g., diseases, signs, symptoms, conditions; for a full list of UMLS semantic types, see Supplementary Table S1) and discarded entities corresponding to interventions (e.g., medications, procedures) and anatomy. We also removed from the analysis all entities with negative polarity (e.g., “Patient denies headache”).

### Clustering complaint feature vectors

We utilized a cluster-based approach to construct HF disease states. The HF disease state clusters used as the basis of this study are constructed from a large dataset (> 100,000 examples) and underwent extensive clinical interpretation; we thus chose K-means as the clustering algorithm because of the inherent interpretability of the resultant cluster centroids and its ability to scale well to large datasets. We aggregated all positively mentioned complaints for each patient over the entirety of the EHR timeline to generate a vector of counts of each complaint for each patient, which was used as the input to K-means clustering. The vector of counts of complaints of each patient was transformed using term frequency-inverse document frequency^41^ (TF-IDF). Templated forms and copy and pasting are common phenomena in clinical text and become even more prevalent in patients with longer records. To reduce the contribution of repeated text chunks and normalize term counts over patients with different timeline lengths, we utilized a logarithmic term frequency, followed by an L2 normalization of each patient vector.

The relative frequency of complaints in the dataset follows a power law distribution. To limit the dimensionality of the representation and to exclude rare terms, we restricted the complaints vocabulary $V$ to 99% the most frequent complaints, which reduced the vocabulary size from 9,375 to 1,276. Vectorization of the entire heart failure cohort results in a two-dimensional matrix of the form $P=({p}_{i,j})\epsilon {R}^{N\times V}$. Here, N denotes the number of snapshots in the cohort (N = 103,833).

### Evaluation of clustering models

We hypothesized that clustering with different $K$ values (numbers of clusters) would group HF snapshots by different levels of disease hierarchy (i.e., small values of K would result in cohorts grouped by broad disease features, whereas a large values of $K$ would results in cohorts grouped by more granular disease features). To determine which values of $K$ yield stable clusters for the HF dataset, we quantified cluster stability for each value of $K$ with a bootstrapping strategy^42^. For values of K that represent true clusters in the underlying dataset, these clusters should be re-identifiable in randomly subsampled portions of the original dataset.

First, for $K\epsilon [\mathrm{2,3},\dots ,30]$, we clustered data points via K-means clustering to obtain reference clustering results $[{C}_{K=1}^{ref}, {C}_{K=2}^{ref}, ..., {C}_{K=30}^{ref}]$. Then for each reference clustering result for each value of K, we utilized a cluster bootstrapping strategy in which we 1) subsampled a fixed fraction of the original dataset ${f}_{d}$ then 2) clustered again to generate a bootstrapped clustering result ${C}_{K=k}^{{f}_{d}}$. We repeated the bootstrapping for varying fractions of data ${f}_{d}\epsilon [0.5, 0.25, ..., 0.0078125]$ to investigate whether the size of the available dataset would limit the level of accuracy and robustness of the cluster identification. This procedure was repeated 1000 times for each value of $K$ and each fraction of data.

To evaluate the stability of clustering results, we compared the similarity of each bootstrapped clustering result to the corresponding reference clustering at each value of $K$. The Jaccard index characterizes how robust the grouping of patients within a cluster is to changes in the specific population of patients through random subsampling of the data set. Similarity was thus calculated using the Jaccard index across the reference and bootstrapped clustering results:

$$J({C}_{K=k}^{ref}, {C}_{K=k}^{{f}_{d}})=\frac{1}{N}{\sum }_{i=1}^{k}{\sum }_{j=1}^{k}{n}_{ij} \cdot {J}_{ij}$$

Here $N$ denotes the number of data points used in both clustering models, ${n}_{ij}$ is the number of datapoints present in cluster $i$ from reference clustering ${C}_{K=k}^{ref}$ and cluster $j$ from bootstrapped clustering ${C}_{K=k}^{{f}_{d}}$. ${J}_{ij}$ is the Jaccard index between in clusters $i$ and $j$:

$${J}_{ij} = \frac{\left|{c}_{i}^{ref} \bigcap {c}_{j}^{{f}_{d}}\right|}{\left|{c}_{i}^{ref} \bigcup {c}_{j}^{{f}_{d}}\right|}$$

For each value of $K$ and each fraction of data, this analysis yields a distribution of 1000 values of $J$, which can be used to quantitatively compare the stability of clustering across different values of $K$. In this formulation, a value of $J=1$ means that the data has been partitioned identically in the reference and bootstrapped clustering (perfect stability). As less data is sampled for bootstrapping (lower values of ${f}_{d}$), we can empirically test the amount of data at which the clustering result breaks down.

To test the stability of the medical concepts characterizing the different patient clusters, we utilized a *semantic similarity* measure based on a modification of the Jaccard index. S*emantic similarity S* quantifies the similarity of the significant complaints associated with each cluster between the reference and bootstrapped clustering results:

$$S({C}_{K=k}^{ref}, {C}_{K=k}^{{f}_{d}})=\frac{1}{N}{\sum }_{i=1}^{k}{\sum }_{j=1}^{k}{n}_{ij} \cdot {S}_{ij}$$

As before, $N$ denotes the number of datapoints used in both clustering models, ${n}_{ij}$ is the number of datapoints present in cluster $i$ from reference clustering ${C}_{K=k}^{ref}$ and cluster $j$ from bootstrapped clustering ${C}_{K=k}^{{f}_{d}}$. ${S}_{ij}$ is the semantic similarity index between in clusters $i$ and $j$, and quantifies the overlap of the significantly associated complaints vocabularies between the two clusters:

$${S}_{ij} = \frac{\left|{v}_{s,i}^{ref} \bigcap {v}_{s,j}^{{f}_{d}}\right|}{\left| {v}_{s,j}^{{f}_{d}}\right|}$$

As with the Jaccard index bootstrapping, we repeated the bootstrapping for varying fractions of data ${f}_{d}\epsilon [\mathrm{0.5,0.25},...,0.0078125]$. Robust identification of local maxima in ${J}_{ij}$ and ${S}_{ij}$ across increasingly diminishing fractions of the data provides the basis for selection of stable clusters.

### Selection of K and constructing disease state hierarchies

To select the valid values of $K$ that can be considered HF disease states at differing levels of hierarchy, we considered local maxima when sampling 50% of data (${f}_{d}=0.5$) resulting in $m$ values of $K\epsilon [{k}_{max}^{1},{k}_{max}^{2},\dots ,{k}_{max}^{m}]$. After choosing to analyze a set of clusters satisfying both local maximum criteria at a given value of $K$, which we denote ${m}^{*}$, we aimed to visualize the hierarchical structure of the clustering result. Doing so allows us to understand which disease states are more similar to each other and thus frame the results at various levels of hierarchy. To do so, we computed the Jaccard index between the clustering results ${C}_{max}^{m*}$ and the clustering result for all local maxima for values of $K$ less than ${m}^{*}$ ${C}_{max}^{m}$, $m \epsilon [{m}^{*}-1,{m}^{*}-2,\dots ,1]$. This results in a Jaccard similarity matrix of size $[{m}^{*} \times m]$; we then computed the pairwise distance between each cluster in ${C}_{max}^{m*}$. We repeated this for each value of $m$, computed the average, then use the resulting combined distance matrix to create a dendrogram using hierarchical clustering (complete linkage). The resulting HF disease state dendrogram allows us to understand the hierarchical relationship between clusters at different values of $K$ and provides a visualization that can be understood as a phylogenetic tree of complaints and symptoms.

### Statistical testing for identification of distinctive features of disease states clusters

To interpret clusters of patients discovered via K-means clustering, we utilized statistical testing to find complaints that were significantly overrepresented within the cluster as compared to the rest of the heart failure population. Doing so allows us to determine the distinguishing features (medical complaints) of each cluster. More specifically, for each cluster $i$ we break the patient matrix $P$ into two submatrices, ${P}_{i}$ and ${P}_{j}$, where ${P}_{i}$ contains the data points of all patients from cluster $i$ ($k=i$) and ${P}_{j}$, contains the data points from all other clusters ($k\ne i$). For each feature $f$, we then use a right-sided t-test to test the null hypothesis that the mean of the TF-IDF features in ${P}_{i}^{f}$ are equal to ${P}_{j}^{f}.$ Rejection of the null hypothesis for feature $f$ means that this complaint is overrepresented in cluster $i$ and can be interpreted as a distinguishing characteristic of the cluster. Performing this test for each complaint within a cluster yields a vocabulary of significantly associated complaints ${V}_{s}$. We employed Bonferroni correction for multiple comparisons. Samples were tested to confirm to be normally distributed.

### Grouping complaint features into clinical phenotypes

The complaint features used in this study were extracted from clinical text and mapped to SNOMED CT, which has a hierarchical structure. As a result, there are terms in the feature vector that are more specific than others. For example, there are features that correspond to *anemia*, a more general term, as well as *iron-deficiency anemia*, *hemolytic anemia*, and *hypochromic anemia*, which are more specific types of anemia. To summarize these hierarchical relationships between disease state features, we defined 50 high-level complaints and utilized a keyword-based search to group input complaint features into these *grouped complaint* clinical phenotypes (Supplementary Table S3).

### Calculating clinical characteristics for HF disease states

In addition to analyzing the NLP-derived concepts, each HF disease state was further characterized by the sex distribution (% female), median age, median BMI, and prevalence of in-hospital mortality. The sex of each patient and in-hospital mortality were derived directly from structured fields within the EHR. For each data element in the de-identified analysis dataset, age was calculated as the difference between the element timestamp and the patient’s date of birth (days), with ages above 90 years set to 90 years (32,872 days); the age used to compute the median age of the disease state snapshot was the age of the patient when they first entered the snapshot, converted to years. The BMI of the patient at each disease state was taken as the average of all BMI measurements taken within the 30 day window. Because BMI is not measured at all visits, for each disease state, the fraction of data points used in the calculation of median BMI is provided in Supplementary Fig. S2.

### Clinical summary of HF disease states

Each HF disease state cluster can be understood as HF patient timeline segments grouped by similar patterns of complaints, symptoms, and comorbidities; disease states are complex and typically multimorbid. Although it is difficult to summarize such complex patterns succinctly, to facilitate communication of results and interpretation of figures, each disease state was given a clinically interpretable name and concise description by the authors (Table 3). The cluster names and descriptions were based on interpretation of Table 2, Supplementary Table S2, and Figs. 2 & 3.

### Defining temporal progression between HF disease states

Once we characterized HF disease states based on complaints over 30-day time windows, we sought to quantify the temporal relationship between these states to understand HF disease evolution. This was accomplished using a two-step process.

#### Quantifying the association between disease states

First, we quantified the likelihood that each disease state (cluster) pair co-occur within a patient’s timeline over the entire HF population. This was done by computing a cluster-wise co-occurrence matrix (Fig. 5b), then computing the odds ratio between each cluster pair^58^ (Fig. 5c). For disease states *i* and *j*, the odds ratio would be computed as ${OR}_{ij}=\frac{{n}_{i,j}{n}_{\neg i,\neg j}}{{n}_{i,\neg j}{n}_{\neg i,j}}$, where ${n}_{i,j}$ is the number of times disease states *i* and *j* cooccur across the HF cohort, ${n}_{i,\neg j}$ is the number of times disease state *i* occurs with any disease state that is not disease state *j*, ${n}_{\neg i,j}$ is the number of times disease state *j* occurs with any disease state that is not disease state *i*, and ${n}_{\neg i,\neg j}$ is the number of disease state co-occurrences across all disease states that are not *i* or *j*. Statistically significant cluster pairs with odds ratios > 1 were considered positively correlated.

#### Temporally ordering disease states

Next, for each cluster pair ${c}_{i}$ and ${c}_{j}$, we computed a count matrix that counts which cluster comes first in patients where both clusters occur (Fig. 5b). Normalizing these counts into probabilities yields the probability that ${c}_{i}$ occurs before ${c}_{j}$ over the entire HF dataset (Fig. 5d).

Combining steps 1 & 2 for each cluster pair, we can both quantify the strength of the association between the clusters and assign a temporal directionality to positively associated disease state clusters. These relationships can be visualized as a graph, which describes the temporal relationship between HF disease states (Fig. 6). To emphasize stronger associations, statistically significant odds ratios > 1.33 were used as edges in the graph. The direction of edges was computed using the temporal ordering of clusters, where probabilities in the range [0.45, 0.55] are shown as bidirectional arrows.

### Statistical package versions

TF-IDF vectorization, K-means clustering, and t-SNE were implemented using Python’s Scikit-learn (0.19.1). Statistical tests were implemented using Scipy (1.3.0).

## Supplementary Information

Supplementary Information 1.

Supplementary Information 2.

## Data Availability

The patient-level electronic health record dataset was made available for this study with permission from the originating health system. The de-identified analysis dataset that supports the findings of this study may be made available to qualified investigators upon request with appropriate institutional review board approval and execution of a data use agreement with Droice Labs. For requests for access to the data, interested researchers should contact data-requests@droicelabs.com.
