# Supplementary material for: Data-driven identification of heart failure disease states and progression pathways using electronic health records
Source: Sci Rep. 2022 Oct 25;12:17871. doi: 10.1038/s41598-022-22398-4 (PMC9596465; doi:10.1038/s41598-022-22398-4)
Supplement: Supplementary file 1 — Supplementary Information 1. [file 41598_2022_22398_MOESM1_ESM.docx]

## Supplementary results

### Metabolic syndrome

**Diabetic complications.** This cluster is dominated by concepts related to diabetes, with 100% of snapshots containing textual references to diabetes. This disease state contains patients with the highest BMI (median 31.86, interquartile range 27.83-35.92); most patients are obese (72.3% of snapshots contain grouped complaints indicating obesity, the highest rate of any disease state), which is consistent with findings of obesity and diabetes risk^1,2^. The grouped complaints (Fig. 2) indicate a population with advanced stages of diabetes with multiple complications (71.7% and 53.7% of snapshots contain a mention of a form of diabetic complication or a vision disorder, respectively); this can be elucidated further by examining other significantly enriched concepts within the cluster, including diabetic nephropathy (*diabetic nephropathy*, 48.4%; kidney *disease*, 24.4%; *chronic kidney disease*, 20.3%; *chronic kidney failure*, 7.82%; *proteinuria*, 15.7%), neuropathy (*diabetic polyneuropathy*, 66.1%; *diabetic foot*, 8.64%), retinopathy (*retinal disease*, 30.1%; *diabetic retinopathy*, 17.1%; *non-proliferative diabetic retinopathy*, 15.7%), and abnormal glucose levels (*poor glycemic control*, 6.75%; *hyperglycemia*, 22.7%; *hypoglycemia*, 13.9%, *glycosuria*, 3.34%).

Heart failure and diabetes frequently co-occur along with high rates of hypertension (mentions in 87.5% of data points) and coronary artery disease (87.1%), and HF in diabetics is associated with poor prognosis^3–6^, which matches the relatively advanced disease state of the patients in this cluster. Interestingly, *endocarditis* is also significantly enriched within this disease state (20.4% prevalence), where previous studies have shown the increasing risk between more advanced stages of diabetic complications and the incidence of infective endocarditis^7^.

**Lipid disorders.** The *Lipid disorders* cluster contains patients that uniformly suffer from hyperlipidemia (100% prevalence) and are typically overweight or obese (median BMI 28.08, interquartile range 25.22-31.62; obesity complaints present in 69.4% of snapshots). These patients also experience *hypertensive disease* in 66.7% of snapshots and associated findings like *increased in blood pressure* in 15.2%, which is consistent with the common association between dyslipidemia and hypertension^8^, as well as both diseases role in being predictors of heart failure^9^. This disease state corresponds to the lowest-acuity setting of care for the HF cohort, with the vast majority of visits classified as ambulatory.

### Valve disease

**Aortic stenosis.** This cluster contains snapshots of HF patients with aortic stenosis (AS). HF is frequent in AS patients^10,11^; within these snapshots, we observe frequent mentions of *chronic heart failure* (83.2% of snapshots), along with many indicators of AS, including *aortic valve stenosis* (94.8%), *aortic valve insufficiency* (61.2%), and *aortic valve calcification* (5.09%), among others. The significantly enriched complaints within the cluster corroborate many other well-known epidemiological patterns in aortic valve disease, including the association between *bicuspid aortic valve* (18.6%), AS, aortic insufficiency and regurgitation, and *infective endocarditis* (7.77%)^12^; and that bicuspid aortic valve is the most common form of *congenital heart disease* (19.9%) and frequently leads to the necessity of a valve surgery in a patient’s 50s or 60s due to AS^13^. This is supported by service codes, which show that over 16% of snapshots in this cluster included a valve repair or replacement procedure. Other complaints in this cohort are consistent with previous findings of cardiovascular risk factors for aortic valve disease, including *hypertensive disease* (81.3%) and *hyperlipidemia* (16.6%), as well as the higher median age of this cluster compared to others (median 68.28, IQR 60.79-76.17)^14^. Interestingly, there is also a relatively high prevalence of mentions of *chronic rheumatic heart disease* (14.0%), which can be a driver of heart valve damage and AS^15^; the Russian Federation has historically high rates of rheumatic heart disease^16^.

### Vascular disease

**Aneurysm.** The *Aneurysm* cluster is dominated by concepts related to aneurysms, including *aneurysm* (99.7%), *aortic aneurysm* (12.7%), and *abdominal aortic aneurysm* (11.1%), among others. There are also mentions of several related concepts, including *thrombus* (31.6%), which can occur with aneurysms^17^, as well as mentions of ischemic heart disease such as *myocardial ischemia* (58.8%), *coronary heart disease* (75.1%), *angina pectoris* (57.6%), and *atherosclerosis* (51.9%), which are often the cause of aneurysms^18^. Interestingly, 71.4% of snapshots in the cluster contain mentions of *myocardial infarction* and 17.8% have mentions of *acute myocardial infarction*, which can be a driver of *left ventricular aneurysm* (18.2%), *dyskinesia* of the heart (16.3%), *ventricular tachycardia* (18.6%), and *thrombosis* (11.2%). The cluster is predominantly male (82.01%), which matches previously reported incidences of aneurysm in males vs. females^19^.

**Cerebrovascular disease.** The *Cerebrovascular disease* cluster contain some of the oldest patients, with a median age of 68.13 years (interquartile range 59.00 to 75.89 years) at the time of their visit. 81.39% of these snapshots contained an ICD-10 code for cerebrovascular disease (I60-I69), which encompasses a range problems including cerebral ischemia, encompassing complaints such as *cerebral atherosclerosis* (42.5% of snapshots) and *carotid stenosis* (7.6%); several sub-classifications of stroke, including *ischemic stroke* (19.8%), *cerebral infarction* (15.1%), *transient ischemic attack* (12.7%), and *cerebrovascular accident*; common sequelae of stroke such as *Broca’s aphasia* (5.36%), *hemianopsia* (6.23%), *hemiparesis* (18.2%), and *spastic hemiplegia* (4.94%); and common neurological findings and symptoms, including *encephalopathy* (82.0%), *senile plaques* (23.4%), *hydrocephalus* (11.0%), *vertigo* (10.8%), *amnesia* (8.83%), and *gliosis* (6.55%). This cluster also contains significantly enriched mentions of other neurological problems, including *traumatic brain injury* (6.13%).

In addition to cerebrovascular and neurologic disease, there are several ophthalmological concepts (e.g., *cataract*, *glaucoma*) and orthopedic concepts (e.g., *spinal diseases*, *lordosis*, *spondylosis*, *spinal stenosis*, *herniated disk*, *kyphosis*, and *osteochondrosis*) significantly enriched within the cluster. Taken together, this is consistent with the older demographics of this cluster, where neurological examinations are likely to reveal age-related eye disorders and orthopedic issues are common and may contribute to falls (especially combined with the neurologic and movement deficits from cerebrovascular disease, including disability falling stroke) and subsequent brain injuries.

**General vascular disease.** Snapshots within this cluster are significantly enriched with concepts related to vascular disease, including *stenosis* (92.2% of snapshots), *atherosclerosis* (59.7%), and *calcification* (28.8%). This disease state contains evidence of vascular disease from various anatomical locations; for example, there is evidence of cerebrovascular disease from ICD-10 codes (Table 2) and mentions of concepts such as *cerebrovascular accident*, *carotid stenosis*, and *senile plaques*, which have been associated with cerebral ischemia^20^. Additionally, concepts such as *lower limb ischemia*, *pain in lower limb*, *intermittent claudication*, and *superficial ulcer* show evidence of peripheral vascular disease^21^, while *atherosclerosis of aorta* and *chronic myocardial ischemia* indicate coronary artery disease.

### Coronary artery disease

There are six HF disease state clusters with various manifestations of coronary artery disease (CAD), the dominant etiology of congestive heart failure^22^. All six clusters primarily represent snapshots of HF patients with CAD and hypertension, two major and often co-occurring risk factors for of heart failure^23^. The majority of these disease states are dominated by males, matching the global patterns of HF with ischemic etiology^24^, and contain patients in their 60s, which supports previous findings that the Russian Federation has unusually high rates of cardiovascular disease before the age of 70^25^. Additionally, these clusters all have a similar chronic disease profile based on their ICD-10 codes and complaint concepts. Within these disease states, three contain snapshots of patients who are primarily seen in an outpatient setting while the other three have higher rates of inpatient and surgical encounters (Table 2).

**Coronary artery disease (outpatient).** Within the CAD disease states, there are three that contain snapshots of patients receiving care in a predominantly outpatient setting, as evidenced by the high rates of ambulatory encounters and correspondingly low rates of inpatient or surgical encounters (Table 2).

The two most similar clusters within this group are *CAD with cerebral involvement* and *CAD with myocardial ischemia*. Both clusters have high rates of *chronic heart failure*, *hypertensive disease*, *hyperlipidemia*, *cerebrovascular disorders*, *diabetes*, *angina pectoris*, *obesity*, *nephritis*, *chronic obstructive pulmonary disease*, and *myocardial ischemia* & *myocardial infarction*, and numerous gastric problems such as *cholelithiasis*. *CAD with cerebral involvement* is so named because of its high rates of *vertebrobasilar insufficiency* (91.6%) and the presence of associated symptoms within the significantly overrepresented concepts, including *dizziness*, *nausea*, *vomiting*, *vertigo*, and *dystonia*^26^. In contrast, *CAD with myocardial ischemia* has higher rates of complaints such as angina and myocardial infarction (Figure 2). Although the overall complaints in these clusters are similar, there is a large sex disparity between the clusters. *CAD with cerebral involvement* has the highest prevalence of females out of all CAD states (61.77%), while *CAD with myocardial ischemia* is 61.68% male. This sex difference is also reflected in the ratio of prevalence of certain complaints between the two states. For example, liver disease (1.38:1), and cardiomyopathy (4.8:1) occur more frequently in *CAD with cerebral involvement*; myocardial infarction (0.56:1) and angina (0.68:1) occur much more frequently in *CAD with myocardial ischemia*. Additionally, there are several other problems that are heavily associated with females that are significantly enriched with *CAD with cerebral involvement*, including *osteochondrosis* and *autoimmune thyroiditis*, as well as male-specific issues like *prostatic hyperplasia* associated with *CAD with myocardial ischemia*.

*CAD with arteriosclerosis***.** This cluster also contains snapshots containing visits of patients with heart failure concomitant with coronary artery disease, as evidenced by mentions of *chronic heart failure* (81.2% of snapshots), *coronary arteriosclerosis* (81.3%), *coronary heart disease* (66.1%), *atherosclerosis* (43.7%), and *myocardial ischemia* (41.0%). Also present are several findings common in heart failure, including *interventricular dyssynchrony*^27^ (14.2%) and *cardiomegaly* (11.4%). These visits also contain significantly enriched mentions of common comorbidities; grouped complaints show enriched prevalence of hyperlipidemia (46.0%), hypertension (79.1%), chronic obstructive pulmonary disease (19.2%), obesity (52.3%), and chronic kidney disease (23.9%). Some notable differences between the *CAD with arteriosclerosis* state and *CAD with cerebral involvement* and *CAD with myocardial ischemia* include the prevalence of certain predictors of morbidity and mortality in HF, including the almost twofold rate of mentions of atrial fibrillation^28,29^ (40.2% vs. 23.6% and 24.9%) and the tenfold rate of mentions of cardiomegaly^28^ (11.4% vs. 0.9% and 0.8%).

**Coronary artery disease (inpatient).** There are three disease states that contain snapshots of patients with CAD with a majority of their encounters corresponding to inpatient and surgical treatment (Table 2).

*Acute coronary syndrome***.** This cluster contains the snapshots of patients undergoing *acute coronary syndrome* (59.2% of snapshots), including myocardial infarction (*myocardial infarction*, 77.60; *acute myocardial infarction*, 56.2%), and unstable angina (*unstable angina*, 62.6%; *progressive angina*, 22.9%). This cluster also contains diagnostic findings associated with myocardial infarction, such as *ST segment elevation* (21.2%) and *ST segment depression* (14.5%). This interpretation is supported by the fact that 34.7% of snapshots contain an ICD-10 code for acute myocardial infarction, 97.70% contain an inpatient visit, 52.06% an ICU visit, 61.85% a surgical procedure, and 63.22% an angiography procedure (Table 2; Supplementary Table S5).

*CAD, high-acuity***.** This cluster contains snapshots of patients with high prevalence of *heart failure* (67.2%), ischemic heart disease and related complaints (including *coronary heart disease*, 95.8%; *myocardial ischemia*, 76.3%; *myocardial infarction*, 77.4%; and *angina pectoris*, 88.4%), and *hypertensive disease* (90.8%). Additionally, this state (along with the other CAD disease states) has significantly enriched mentions of comorbidities including diabetes, stomach disease, and COPD (Table 2, Figure 2). Although this state also has high mentions of myocardial infarction, this state lacks diagnostic codes indicating an acute myocardial infarction during the snapshot; this disease state can thus be understood as patients with a previous MI.

*Cardiac surgery***.** Finally, the *Cardiac surgery* cluster contains patients who suffer from ischemic heart disease (75.9% of the snapshots in this cluster contained complaints of *myocardial ischemia*, and 85.3% had complaints of *coronary heart disease*, among others) and underwent cardiac surgery (as evidenced by concepts such as *postpericardiotomy syndrome* in 38.1%, *surgical fistula* in 27.7%, and *wound healing* in 25.1% of patients). Additionally, high prevalence of concepts such as *central venous pressure finding* and *pulmonary artery pressure* indicate the placement of an arterial line, indicating a surgical or other high acuity setting. This interpretation was confirmed via service codes, which reveal that 97.68% of these snapshots included a surgical procedure, including coronary artery bypass grafting (CABG, 79.68% of snapshots) and heart valve repair or prosthesis (23.18%).

### Atrial fibrillation & advanced heart failure

**Advanced & decompensated HF**. This cluster contains patients with late-stage heart failure. They experience frequent mentions of *chronic heart failure* (95.50% of snapshots), as well *as* higher levels of complaints associated with later stages of heart failure, including *dyspnea* (88.6%), *edema* (73.4%) and *peripheral edema* (49.4%), *cardiac asthma* (33.4%), and *cyanosis* (20.6%), among others. As is typical with late-stage heart failure, patients with visits in this cluster also experience poor outcomes and decompensation related to heart failure. This interpretation is supported by the highest in-hospital mortality rate (11.69%) of all clusters, as well as numerous significantly associated problems such as *decompensation* (43.% of snapshots), *kidney failure* (10.9%), *respiratory failure* (17.7%), *multiple organ failure* (11.3%), *cardiac arrest* (11.1%), and *sudden cardiac death* (6.77%). Snapshots in this cluster also have multiple comorbidities associated with higher rates of poor cardiovascular outcomes and decompensation, such as *chronic kidney disease*, *atrial fibrillation*, *chronic obstructive pulmonary disease*, and *anemia*^29–32^. Additionally, high rates of *atrial fibrillation* and *dilated cardiomyopathy* in acute decompensated heart failure patients is supported by the literature^33,34^.

**Atrial fibrillation.** This cluster is named for the high prevalence of complaints such as *atrial fibrillation and flutter* (mentions in 89.1% of snapshots), *atrial fibrillation* (87.4%), *paroxysmal atrial fibrillation* (43.1%), *atrial flutter* (39.8%), and *persistent atrial fibrillation* (20.7%), among several others. These snapshots also record high incidences of *chronic heart failure* (80.9%), which commonly co-occurs with atrial fibrillation; the cluster also exhibits several of the common comorbidities that frequently occur with these two conditions, including *obesity* (38.0%) and *hypertensive disease* (78.7%)^35^. Interestingly, *hypothyroidism* (9.81%) and *subclinical hypothyroidism* (8.06%) are significantly associated with the cluster, although previous studies have shown an inverse relationship between atrial fibrillation and hypothyroidism^36^; this may be explained by the predisposition of patients with hypothyroidism to get heart failure, obesity, *hyperlipidemia* (16.1% of snapshots), and some of the other cardiovascular risk factors within the cluster.

### Cardiomyopathy

**Dilated cardiomyopathy.** Snapshots with dilated cardiomyopathy (DCM) clustered well, with 96.40% of snapshots containing mentions of DCM (compared to 8.31% of snapshots outside the cluster) and 87.20% of snapshots with an I42 ICD-10 code for cardiomyopathy. Within these snapshots, the patients exhibited typical complaints for DCM, including *dyspnea* (61.9% of snapshots), *cardiac asthma* (8.71%), *edema* (37.3%), *swelling* (17.4%), and *ascites* (7.13%); valve insufficiencies such as *mitral valve insufficiency* (62.7%) and *tricuspid valve insufficiency* (46.5%); arrhythmias including *ventricular tachycardia* (29.0%)/*paroxysmal ventricular tachycardia* (18.0%), *atrial fibrillation* (32.7%), *premature ventricular contractions* (47.6%), and *interventricular dyssynchrony* (8.49%); clotting events including *pulmonary thromboembolism* (9.27%); and *pulmonary hypertension* (25.0%).

Although incidence of DCM is typically biased towards men^37^, there is a disproportionally low proportion of females in the cohort (23.1%). This was hypothesized to be due to the Russian origin of the dataset, where rates of alcohol consumption in males and corresponding alcoholic cardiomyopathy are high^38,39^. This is supported by EHR templates documenting lifestyle risk factors; within the DCM group, 30.1% of the patients had documentation of alcohol consumption as a lifestyle factor. Thus, this cluster can also be interpreted as *Alcoholic cardiomyopathy*. This interpretation is corroborated by some of the comorbid conditions significantly enriched within the cluster, including *chronic liver disease* (41.4% of snapshots) and *chronic obstructive airway disease* (18.7%)^40^.

**Pediatric cardiomyopathy.** The *Pediatric cardiomyopathy* cluster contains patients with a median age of 10.13 (3.05, 15.43 IQR). Based on the significant concepts associated with this group, this cluster seems to contain a mix of the youngest heart failure patients (excluding neonates with congenital heart disease). First, 72.8% of snapshots within this group had mentions of *myocarditis* mentioned in their notes (with *pericarditis*, 50.4%, also significantly associated), which is known to cause cardiomyopathy^41^ and lead to heart failure in children^42^. Additionally, this cluster also contains another group of patients with peripartum cardiomyopathy, as evidenced by significant concepts such as *pregnancy*, *childbirth*, *primigravida*, *premature birth*, *breast feeding*, *preeclampsia*, *fetal movement*, and *duration of gestation*, where previous studies have outlined the close relationship between pregnancy, myocarditis, and cardiomyopathy^43^. Thus, this cluster can be understood as HF in pediatric patients and mothers with pregnancy-related complications. The algorithmic approach did not separate the two groups into two independent clusters at the selected number of clusters K=23.

**Hypertrophic cardiomyopathy.** Patients with HCM were well-clustered within the HF cohort; the complaint *hypertrophic cardiomyopathy* is mentioned in the clinical text 100% of the snapshots of the eponymous cluster (and is only present in 4.15% of snapshots outside the cluster), and 88.74% of these snapshots contained a visit with the I42 ICD-10 code for cardiomyopathy. These patients also exhibit complaints that are typical complications of HCM, including *syncope* (10.2% of patients), *heart murmur* (18.8%), and *mitral valve insufficiency* (53.9%)^44^. Interestingly, although most studies show a bias toward men of up to 60% in HCM cohorts, our study contains a relatively gender-balanced cohort (54.31% female), which is consistent with the autosomal dominant inheritance pattern of this disorder^45^.

### Congenital heart defects

There are two clusters that contain patients with congenital heart disease (*Congenital heart defects* and *NICU*). With a median age of 22.79 (IQR 3.22-50.63) and 0.50 (IQR 0.08, 2.68), respectively, these two clusters contain some of the youngest patients with congenital heart defects as the etiology of heart failure. This is evidenced by the concepts associated with each cluster (discussed below), as well as the high rate of diagnostic coding for congenital malformations of the circulatory system.

**Congenital heart disease.** This cluster contains snapshots of patients with *congenital heart disease* (mentions in 90.80% of snapshots), primarily *atrial septal defects* (100%). This cluster is predominantly female (62.98%), which is consistent with previous findings that atrial septal defects (ASD) occur more frequently in females than males at a ratio of approximately 2:1^46–48^. This cluster also contains frequent diagnostic findings associated with ASD, such as *heart murmur* (21.5%), *systolic murmur* (23.8%), and *right ventricular hypertrophy* (10.0%), as well as symptoms resulting from ASD, such as *pulmonary hypertension* (29.2%)^49–52^. Additionally, ASD has been associated with respiratory insufficiency and recurrent respiratory infections^52,53^, which is also found within this group in the form of significantly higher mentions of *respiration disorders* (14.1%), *viral respiratory infections* (18.3%), and *acute respiratory disease* (6.26%) than the rest of the HF cohort. With a relatively young median age of 22.79 (3.22-50.63), the patients falling into this group within the HF cohort are likely to represent patients with ASD ultimately leading to diastolic heart failure^52,53^.

**NICU.** While this cluster also contains patients with *congenital heart disease* (mentions in 95.00% of snapshots) such as *atrial septal defects* (42.5% of snapshots), *ventricular septal defects* (60.5%), and *transpositions of great vessels* (8.44%), visits in this cluster represent a high-acuity setting. This is supported by concept mentions such as *central venous pressure finding* (27.0%), which indicate the placement of an arterial line, and *surgical fistula* (18.1%), as well as the finding that 86.01% of these snapshots contain inpatients visits and over 65% contain some form of surgery. In contrast to the *Congenital heart disease* cluster, this cluster also experiences snapshots with poorer outcomes. This is supported by concept mentions such as *respiratory failure* (29.9%), *air embolism* (28.9%), *acute kidney failure* (12.6%), and *ventricular hemorrhage* (11.6%), and the fact that 9.81% of snapshots in this cluster resulted in in-hospital mortality, the highest mortality rate of any cluster. This may be in part explained by some of the other concepts associated with this cluster, including *premature birth* (20.0%), *fetal growth retardation* (7.73%), and *Down syndrome* (6.23%), suggesting that this cluster has more comorbidities or problems that are associated with poorer outcomes^54,55^.

### Diagnostics

**Electrocardiography.** There are two clusters that contain snapshots that primarily contain findings associated with electrocardiography (ECG) findings (*Electrocardiography with apnea*; and *Electrocardiography*. Both clusters contain concepts are frequent findings on ECG, including *cardiac arrhythmia*, *ventricular arrhythmia*, *premature ventricular contractions*, *atrioventricular block*, *atrial fibrillation*, *bradycardia*, and *tachycardia*, among others. *Electrocardiography with apnea* additionally contains respiratory complaints such as *apnea* and *slow shallow breathing* found during sleep studies.

**Echocardiography.** This cluster contains primarily echocardiography findings. This cluster had the highest prevalence of snapshots with an echo (over 99% of snapshots, Supplementary Table S5). Examples of echo findings include *valve insufficiency* of all four heart valves, *diastolic dysfunction*, *left ventricular hypertrophy*, and *stenosis*, as well as other findings such as *cardiomegaly*.

### Other

**Non-CV encounters.** This cluster contains snapshots containing a myriad of complaints, although no complaints dominate a majority of the visits. It has a lower rate of cardiovascular ICD-10 codes and complaints than other clusters, and there are several groups of non-cardiovascular related complaints such as autoimmune disease (*toxic diffuse goiter*, *autoimmune thyroiditis*, *Graves disease*, *lupus erythematosus*, and *rheumatoid arthritis*, among others) blood cancers (*multiple myeloma*, *lymphoma*, *chronic lymphocytic leukemia*, and *chronic myeloid leukemia*), and gynecological and obstetric findings (*pregnancy*, *gynecological history*, *menopause present*, and *fibroid tumor*, among others).

## Supplementary references

S1. Al-Goblan, A. S., Al-Alfi, M. A. & Khan, M. Z. Mechanism linking diabetes mellitus and obesity. *Diabetes. Metab. Syndr. Obes.* **7**, 587–591 (2014).

S2. Felber, J.-P. & Golay, A. PAPER Pathways from obesity to diabetes. *Int. J. Obes.* **26**, 39–45 (2002).

S3. Rosano, G. M., Vitale, C. & Seferovic, P. Heart Failure in Patients with Diabetes Mellitus. *Card. Fail. Rev.* **3**, 52–55 (2017).

S4. Bell, D. S. H. *Heart Failure The frequent, forgotten, and often fatal complication of diabetes*. (2003).

S5. Bertoni, A. G. *et al.* *Heart Failure Prevalence, Incidence, and Mortality in the Elderly With Diabetes*. http://care.diabetesjournals.org (2004).

S6. Lehrke, M. & Marx, N. Diabetes Mellitus and Heart Failure. *Am. J. Cardiol.* **120**, S37–S47 (2017).

S7. Østergaard, L. *et al.* Duration and complications of diabetes mellitus and the associated risk of infective endocarditis. *Int. J. Cardiol.* **278**, 280–284 (2019).

S8. O, H. R. *et al.* Dyslipidemia and the Risk of Incident Hypertension in Men. *Hypertension* **47**, 45–50 (2006).

S9. Biykem, B. *et al.* Contributory Risk and Management of Comorbidities of Hypertension, Obesity, Diabetes Mellitus, Hyperlipidemia, and Metabolic Syndrome in Chronic Heart Failure: A Scientific Statement From the American Heart Association. *Circulation* **134**, e535–e578 (2016).

S10. Spitzer, E. *et al.* Aortic Stenosis and Heart Failure: Disease Ascertainment and Statistical Considerations for Clinical Trials. *Card. Fail. Rev.* **5**, 99–105 (2019).

S11. Kamperidis, V. *et al.* Diagnosis and management of aortic valve stenosis in patients with heart failure. *European Journal of Heart Failure* vol. 18 469–481 (2016).

S12. Ward, C. Clinical significance of the bicuspid aortic valve. *Heart* **83**, 81 (2000).

S13. Iung, B. *et al.* A prospective survey of patients with valvular heart disease in Europe: The Euro Heart Survey on Valvular Heart Disease. *Eur. Heart J.* **24**, 1231–1243 (2003).

S14. Rajamannan, N. M. *et al.* Calcific aortic valve disease: not simply a degenerative process: A review and agenda for research from the National Heart and Lung and Blood Institute Aortic Stenosis Working Group. Executive summary: Calcific aortic valve disease-2011 update. *Circulation* **124**, 1783–1791 (2011).

S15. Afifi, A., Hosny, H. & Yacoub, M. Rheumatic aortic valve disease-when and who to repair? *Ann. Cardiothorac. Surg.* **8**, 383–389 (2019).

S16. Folomeeva, O., Amirdzhanova, V., Iakusheva, E., Lobareva, L. & Loginova, Ei. Incidence rate of rheumatic diseases in Russian population: 10-year analysis. *Ter Arkh* **74**, 5–11 (2002).

S17. Cabin, H. & Roberts, W. Left Ventricular Aneurysm , lntraaneurysmal Thrombus and Systemic Embolus in Coronary Heart Disease *. in (2005).

S18. Hernesniemi, J. A., Vänni, V. & Hakala, T. The prevalence of abdominal aortic aneurysm is consistently high among patients with coronary artery disease. *J. Vasc. Surg.* **62**, 232-240.e3 (2015).

S19. Makrygiannis, G. *et al.* Sex Differences in Abdominal Aortic Aneurysm: The Role of Sex Hormones. *Ann. Vasc. Surg.* **28**, 1946–1958 (2014).

S20. Kalaria, R. N. The role of cerebral ischemia in Alzheimer’s disease. *Neurobiol. Aging* **21**, 321–330 (2000).

S21. Gerhard-Herman, M. D. *et al.* 2016 AHA/ACC guideline on the management of patients with lower extremity peripheral artery disease: Executive Summary: A report of the American college of cardiology/American Heart Association task force on clinical practice guidelines. *Circulation* vol. 135 e686–e725 (2017).

S22. Remme, W. J. *Overview of the Relationship Between Ischemia and Congestive Heart Failure*. *Clin. Cardiol* vol. 23 (2000).

S23. Velagaleti, R. S. & Vasan, R. S. Heart failure in the twenty-first century: is it a coronary artery disease or hypertension problem? *Cardiol. Clin.* **25**, 487–v (2007).

S24. Bui, A. L., Horwich, T. B. & Fonarow, G. C. Epidemiology and risk profile of heart failure. *Nature Reviews Cardiology* vol. 8 30–41 (2011).

S25. Lakunchykova, O. *et al.* Why does Russia have such high cardiovascular mortality rates? Comparisons of blood-based biomarkers with Norway implicate non-ischaemic cardiac damage. *J. Epidemiol. Community Health* **74**, 698–704 (2020).

S26. Lima Neto, A. *et al.* Pathophysiology and Diagnosis of Vertebrobasilar Insufficiency: A Review of the Literature. *Int. Arch. Otorhinolaryngol.* **21**, 302–307 (2017).

S27. Ghio, S. *et al.* Interventricular and intraventricular dyssynchrony are common in heart failure patients, regardless of QRS duration. *Eur. Heart J.* **25**, 571–578 (2004).

S28. Pocock, S. J. *et al.* Predictors of mortality and morbidity in patients with chronic heart failure. *Eur. Heart J.* **27**, 65–75 (2006).

S29. Ford, I. *et al.* Top ten risk factors for morbidity and mortality in patients with chronic systolic heart failure and elevated heart rate: The SHIFT Risk Model. © 2015 Elsevier Ireland Ltd. All rights reserved. *Int. J. Cardiol.* **184**, 163–169 (2015).

S30. Joseph, S. M., Cedars, A. M., Ewald, G. A., Geltman, E. M. & Mann, D. L. Acute decompensated heart failure: contemporary medical management. *Texas Hear. Inst. J.* **36**, 510–520 (2009).

S31. J, P. N. *et al.* Association of Gout With Long‐Term Cardiovascular Outcomes Among Patients With Obstructive Coronary Artery Disease. *J. Am. Heart Assoc.* **7**, e009328 (2018).

S32. Farmakis, D., Parissis, J., Lekakis, J. & Filippatos, G. Acute Heart Failure: Epidemiology, Risk Factors, and Prevention. *Rev. Española Cardiol. (English Ed.* **68**, 245–248 (2015).

S33. Nieminen, M. S. *et al.* EuroHeart Failure Survey II (EHFS II): a survey on hospitalized acute heart failure patients: description of population. *Eur. Heart J.* **27**, 2725–2736 (2006).

S34. P, D. J. Atrial Fibrillation and Acute Decompensated Heart Failure. *Circ. Hear. Fail.* **2**, 72–73 (2009).

S35. Anter, E., Jessup, M. & Callans, D. J. Atrial fibrillation and heart failure: Treatment considerations for a dual epidemic. *Circulation* vol. 119 2516–2525 (2009).

S36. Udovcic, M., Pena, R. H., Patham, B., Tabatabai, L. & Kansara, A. Hypothyroidism and the Heart. *Methodist Debakey Cardiovasc. J.* **13**, 55–59 (2017).

S37. Fairweather, D. L., Cooper, L. T. & Blauwet, L. A. Sex and Gender Differences in Myocarditis and Dilated Cardiomyopathy. *Curr. Probl. Cardiol.* **38**, 7–46 (2013).

S38. Leon, D. A., Shkolnikov, V. M., McKee, M., Kiryanov, N. & Andreev, E. Alcohol increases circulatory disease mortality in Russia: Acute and chronic effects or misattribution of cause? *Int. J. Epidemiol.* **39**, 1279–1290 (2010).

S39. Keenan, K. *et al.* Social factors influencing Russian male alcohol use over the life course: A qualitative study investigating age based social norms, masculinity, and workplace context. *PLoS One* **10**, (2015).

S40. Goldberg, D. S. & Fallon, M. B. The Art and Science of Diagnosing and Treating Lung and Heart Disease Secondary to Liver Disease. *Clin. Gastroenterol. Hepatol.* **13**, 2118–2127 (2015).

S41. Mason, J. W. Myocarditis and dilated cardiomyopathy: An inflammatory link. *Cardiovascular Research* vol. 60 5–10 (2003).

S42. Canter, C. E. & Simpson, K. P. Diagnosis and treatment of myocarditis in children in the current era. *Circulation* **129**, 115–128 (2014).

S43. Midei, M. G., Dement, S. H., Feldman, A. M., Hutchins, G. M. & Baughman, K. L. *Peripartum Myocarditis and Cardiomyopathy*. http://ahajournals.org.

S44. Maron, B. J. *Hypertrophic Cardiomyopathy A Systematic Review*. https://jamanetwork.com/.

S45. Siontis, K. C., Ommen, S. R. & Geske, J. B. Sex, Survival, and Cardiomyopathy: Differences Between Men and Women With Hypertrophic Cardiomyopathy. *Journal of the American Heart Association* vol. 8 e014448 (2019).

S46. Engelfriet, P. & Mulder, B. J. M. Gender differences in adult congenital heart disease. *Neth. Heart J.* **17**, 414–417 (2009).

S47. Amel-Shahbaz, S. *et al.* The epidemiological aspects of congenital heart disease in central and southern district of Iran. *Adv. Biomed. Res.* **3**, 233 (2014).

S48. Craig, R. J. & Selzer, A. Natural history and prognosis of atrial septal defect. *Circulation* **37**, (1968).

S49. Christensen, D. D., Vincent, R. N. & Campbell, R. M. Presentation of Atrial Septal Defect in the Pediatric Population. *Pediatr. Cardiol.* **26**, 812–814 (2005).

S50. DIAMOND, M. A., DILLON, J. C., HAINE, C. L., CHANG, S. & FEIGENBAUM, H. Echocardiographic Features of Atrial Septal Defect. *Circulation* **43**, 129–135 (1971).

S51. Bradley, E. A. & Zaidi, A. N. Atrial Septal Defect. *Cardiology Clinics* vol. 38 317–324 (2020).

S52. Martin, S. S., Shapiro, E. P. & Mukherjee, M. Atrial septal defects – Clinical manifestations, echo assessment, and intervention. *Clinical Medicine Insights: Cardiology* vol. 8 93–98 (2015).

S53. Le Gloan, L., Legendre, A., Iserin, L. & Ladouceur, M. Pathophysiology and natural history of atrial septal defect. *J. Thorac. Dis.* **10**, S2854–S2863 (2018).

S54. Lopes, S. A. V. do A. *et al.* Mortality for Critical Congenital Heart Diseases and Associated Risk Factors in Newborns. A Cohort Study. *Arq. Bras. Cardiol.* **111**, 666–673 (2018).

S55. Freeman, S. B. *et al.* Ethnicity, sex, and the incidence of congenital heart defects: a report from the National Down Syndrome Project. *Genet. Med.* **10**, 173–180 (2008).

## Supplementary figures

**
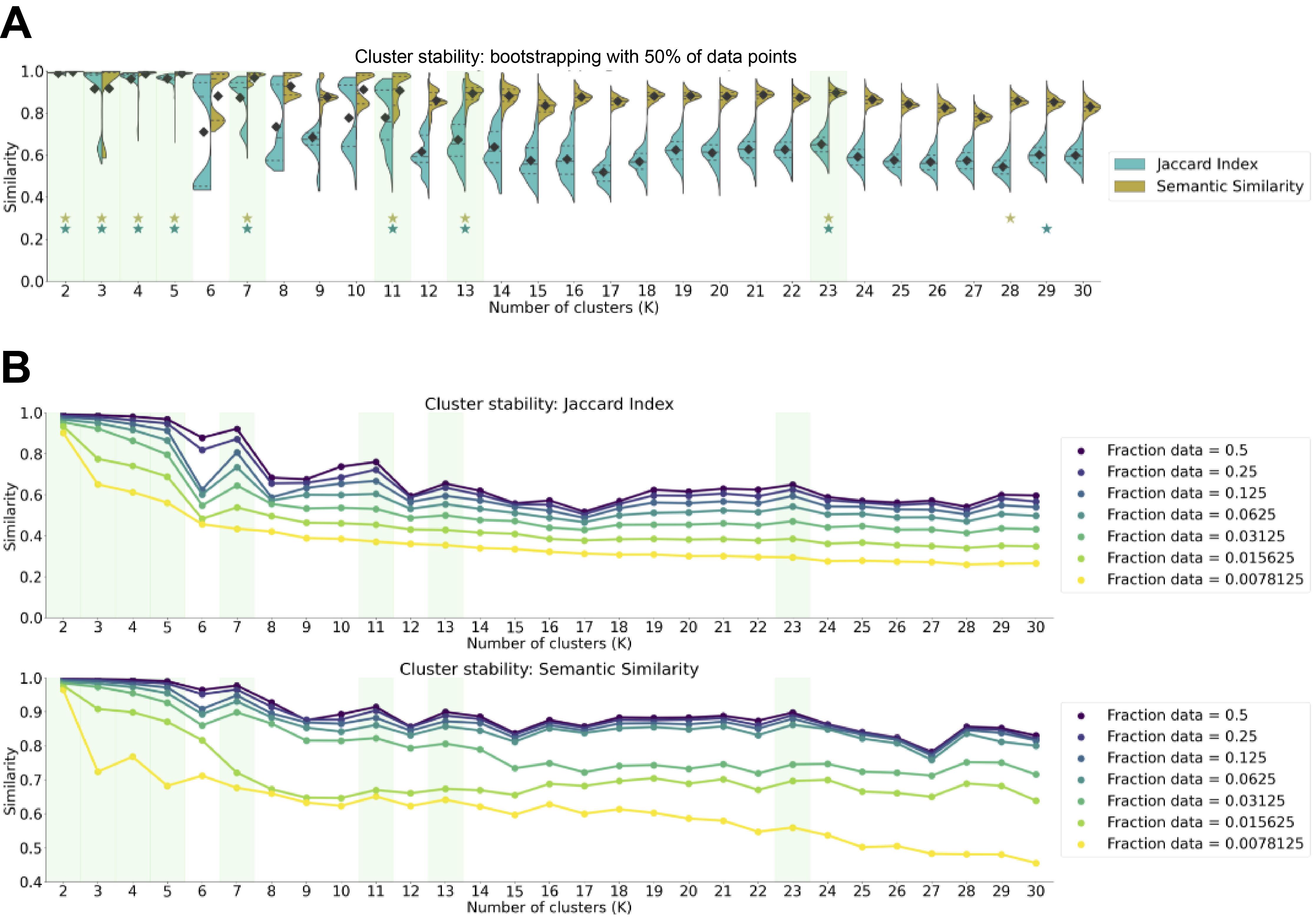
**

*Supplementary Figure S1: Cluster bootstrapping results. (A) Violin plot depicting the distributions of Jaccard index (left violin, cyan) and semantic similarity values (right violin, khaki) between original K-means clustering and clustering on a bootstrapped dataset subsampling 50% of the dataset (number of iterations = 1000). Diamonds mark the median of the distribution, while dotted lines denote the 25^th^/75^th^ quartiles. Local maxima for the Jaccard index/semantic similarity are marked with cyan/khaki stars. Green corridors show values of K for which the Jaccard index and semantic similarity are both local maxima, K ≤23. (B) Median values of the Jaccard index (top) and semantic similarity (bottom) between original K-means clustering and clustering on a bootstrapped dataset subsampling for fractions of data in [0.5, 0.25, …, 0.0078125].*


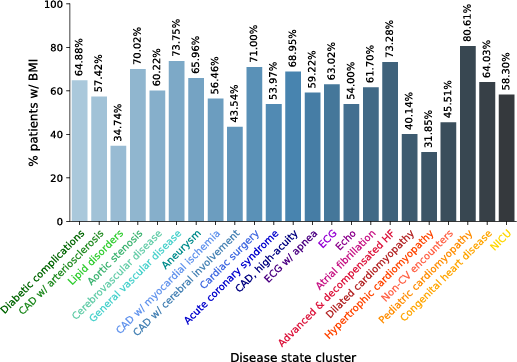


*Supplementary Figure S2: Percentage of patients in each disease state cluster with at least one BMI measurement.*

*
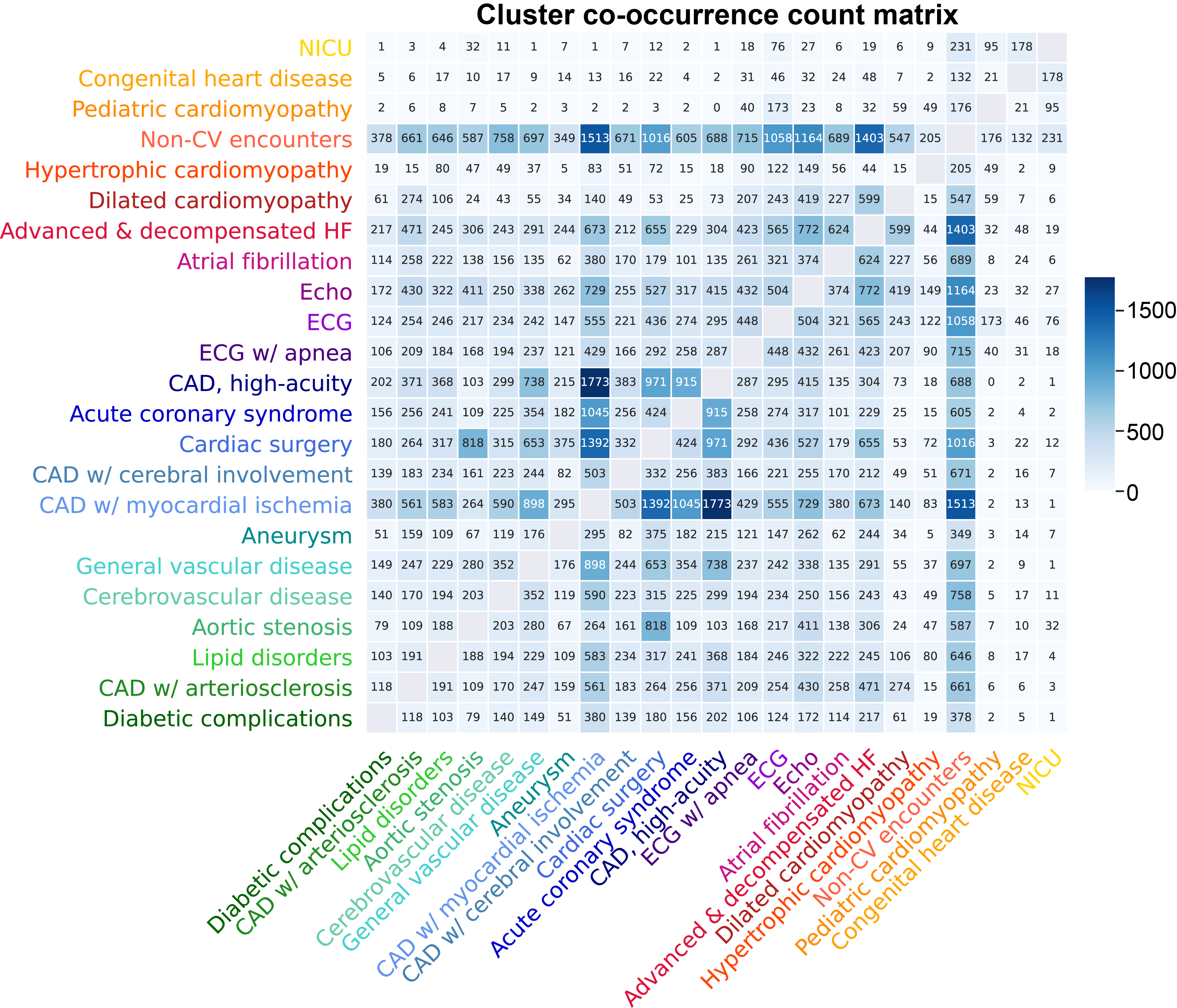
*

*Supplementary Figure S3: Counts of disease state clusters co-occurring within HF patient timelines. Each element (i,j) in the matrix denotes the number of times that cluster k_i_ and k_j_ co-occurred within a patient’s timeline across the entire HF cohort.*

*
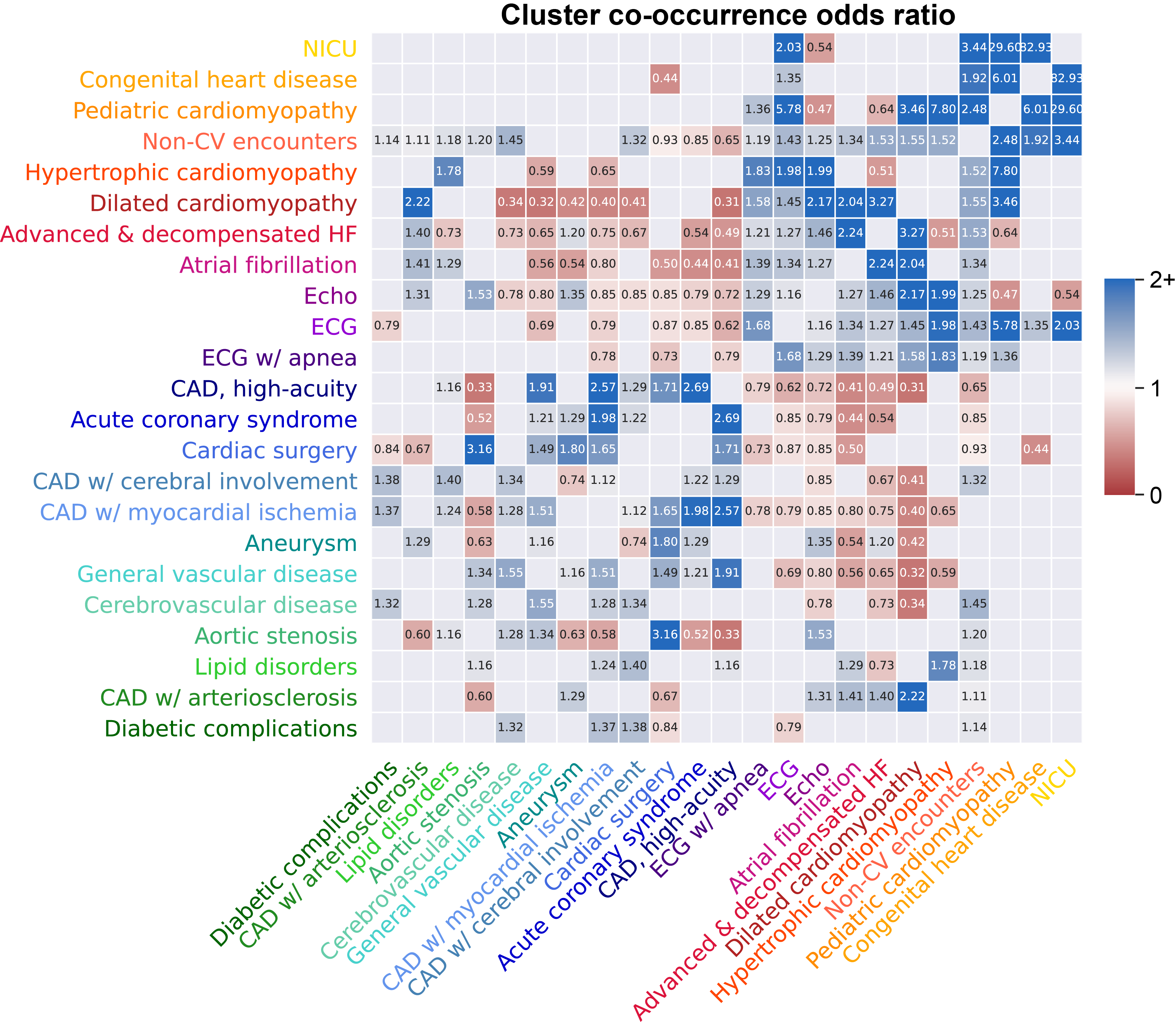
*

*Supplementary Figure S4: Odds ratio of disease state clusters co-occurring within HF patient timelines. Each element (i,j) in the matrix denotes the odds ratio that cluster k_i_ and k_j_ co-occurr within a patient’s timeline across the entire HF cohort. Values > 1 (blue) show positive associations, while values < 1 show negative associations (red).*


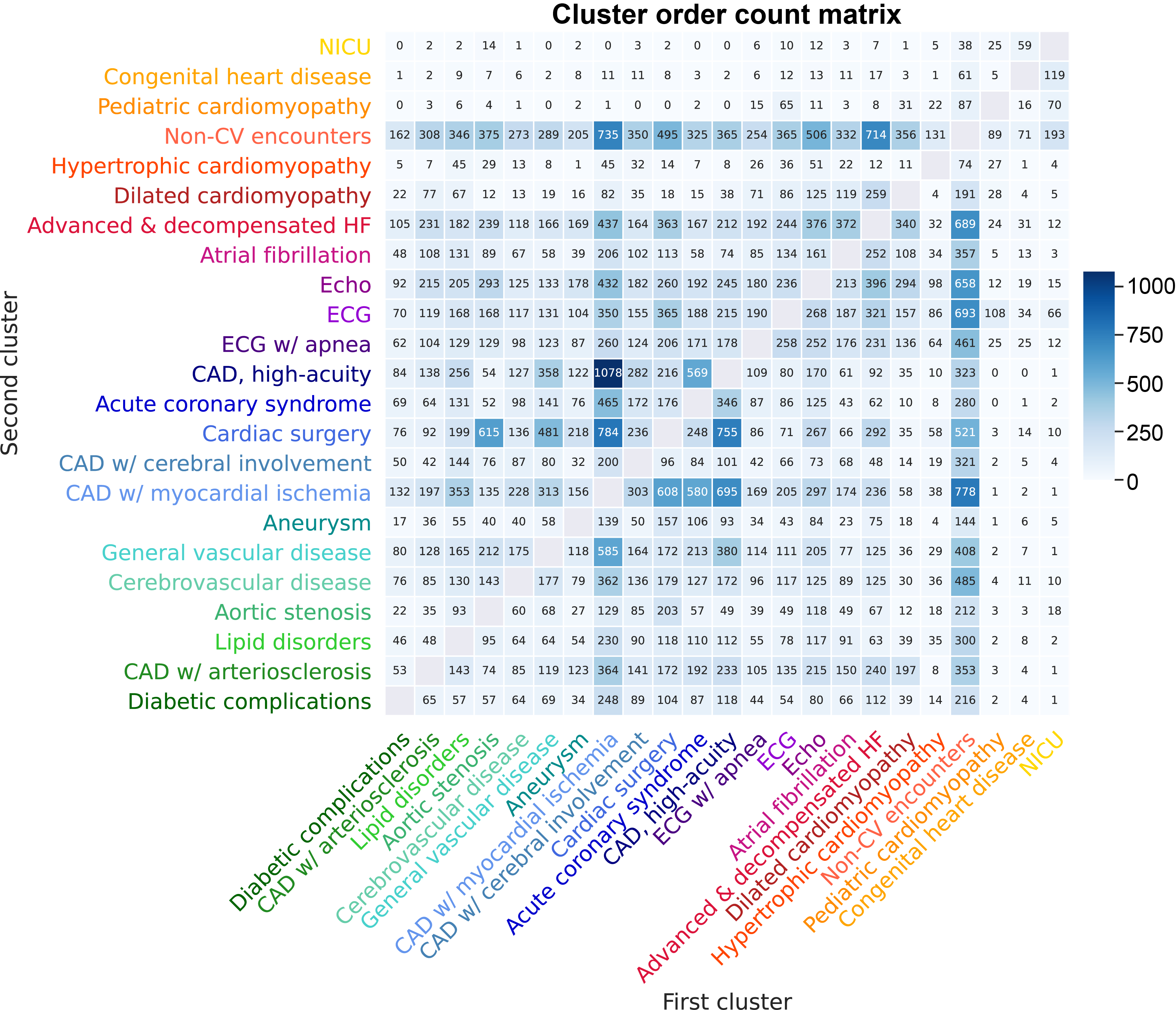


*Supplementary Figure S5: Cluster order counts of disease state clusters co-occurring within HF patient timelines. Each element (i,j) in the matrix denotes the number of times that cluster k_i_ occurred before cluster k_j_ when they both co-occur within a patient’s timeline, across the entire HF cohort.*


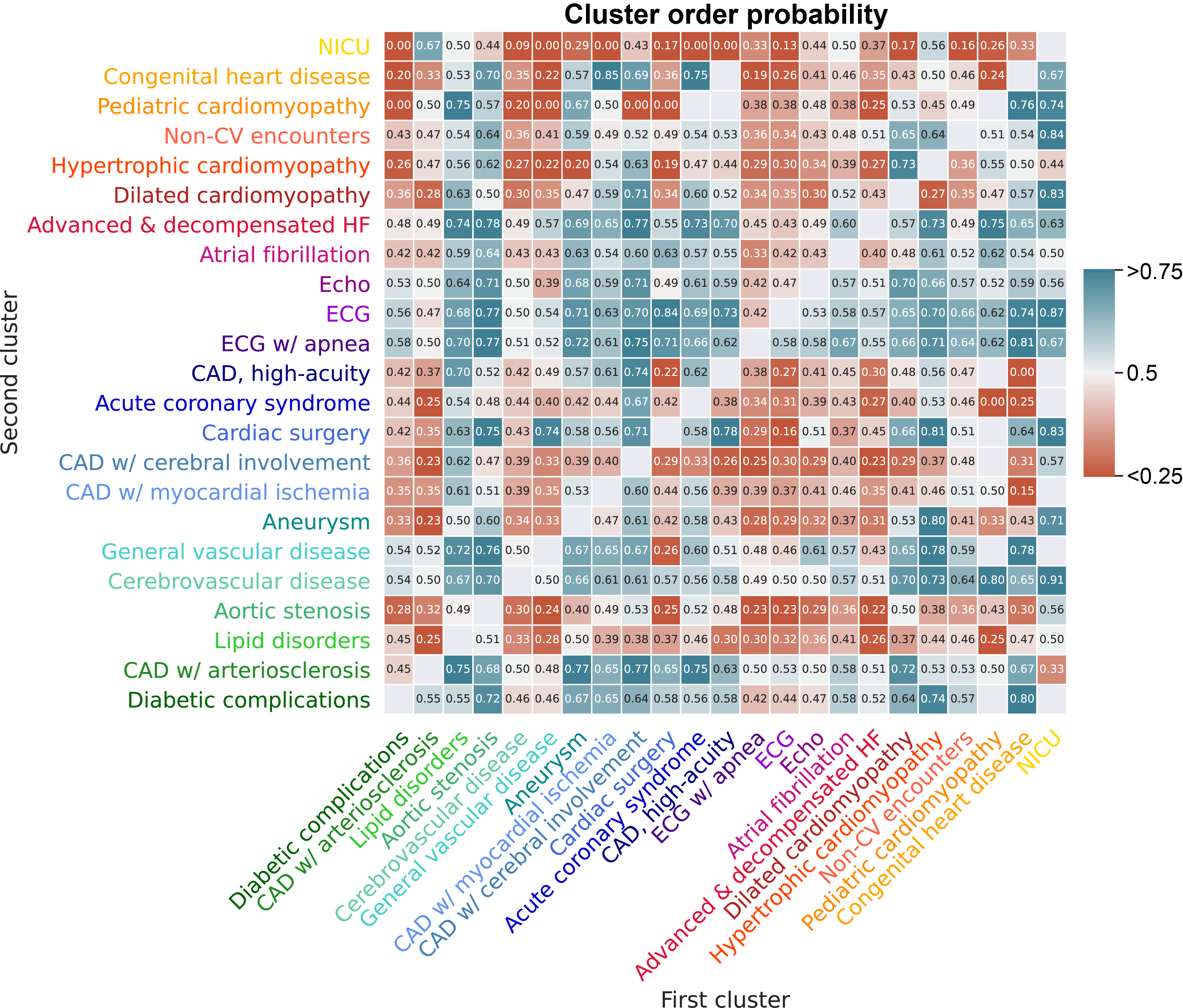


*Supplementary Figure S6: Cluster order probabilities of disease state clusters co-occurring within HF patient timelines. Each element (i,j) in the matrix denotes the probability that cluster k_i_ occurred before cluster k_j_ when they both co-occur within a patient’s timeline, across the entire HF cohort. Values in blue show a higher probability (<50%), while values in red show a lower probability (>50%).*


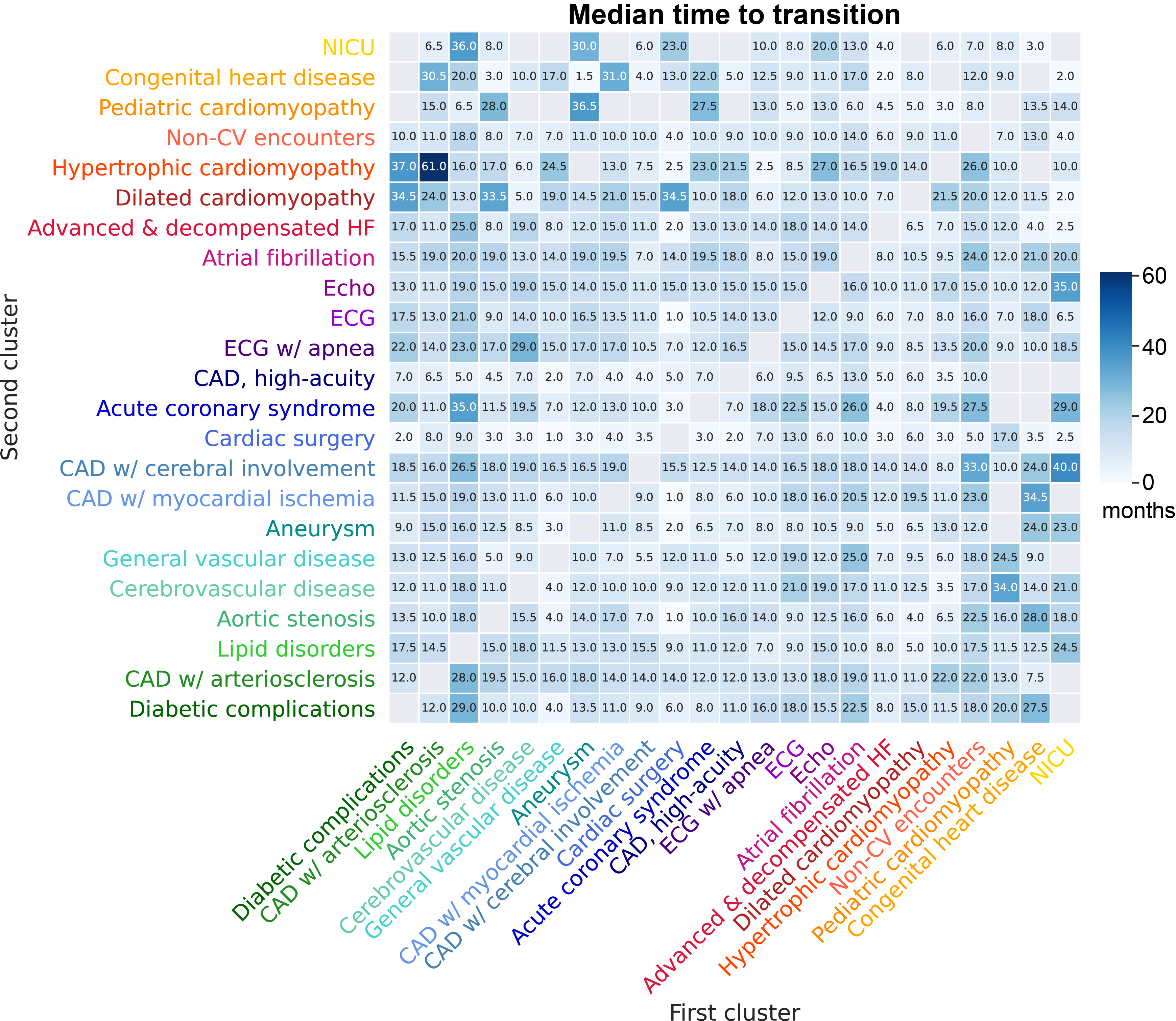


*Supplementary Figure S7: Median time to transition between disease state clusters co-occurring within HF patient timelines. Each element (i,j) in the matrix denotes the median time it took for cluster k_i_ to transition to cluster k_j_ when they both co-occur within a patient’s timeline, across the entire HF cohort.*

## Supplementary tables

| TUI | Semantic type description |
| --- | --- |
| T020 | Acquired Abnormality |
| T190 | Anatomical Abnormality |
| T049 | Cell or Molecular Dysfunction |
| T019 | Congenital Abnormality |
| T047 | Disease or Syndrome |
| T050 | Experimental Model of Disease |
| T033 | Finding |
| T037 | Injury or Poisoning |
| T048 | Mental or Behavioral Dysfunction |
| T191 | Neoplastic Process |
| T046 | Pathologic Function |
| T184 | Sign or Symptom |

*Supplementary Table S1: List of UMLS type unique identifiers (TUIs) used to denote complaints.*

See **supplementary_table_s2.xlsx**

*Supplementary Table S2: Statistically significant concepts in each disease state cluster.*

| Complaint | Matched terms | |
| --- | --- | --- |
| Acute coronary syndrome | | Acute coronary syndrome |
| Amyloid plaques | | Senile plaques |
| Anemia | | Anemia; Iron deficiency anemia; Chronic anemia; Hypochromic anemia; Anemia of chronic disease; Anemia of prematurity; Deficiency anemias; Normocytic normochromic anemia; Hemolytic anemia; Normocytic anemia; Microcytic hypochromic anemia |
| Aneurysm | | Aneurysm; Left ventricular aneurysm; Aortic aneurysm; Pseudoaneurysm; Abdominal aortic aneurysm; Aneurysmal bone cysts; Microaneurysm; Saccular aneurysm; Aneurysm of ascending aorta; Dissecting aneurysm; Aneurysm of infrarenal abdominal aorta; Abdominal aortic aneurysm without rupture; Heart aneurysm |
| Angina | | Angina pectoris; Unstable angina; Exercise-induced angina; Progressive angina; Cardiac pain; Variant angina pectoris; Stable angina; Typical angina |
| Aphasia | | Broca aphasia; Aphasia; Mixed aphasia |
| Arthritis | | Gouty arthritis; Degenerative polyarthritis; Rheumatoid arthritis; Hyperuricemia without signs of inflammatory arthritis and tophaceous disease; Osteoarthritis of hip; Arthritis; Knee osteoarthritis; Chronic gouty arthritis; Seropositive rheumatoid arthritis; Generalized osteoarthritis; Polyarthritis; Psoriatic arthritis; Periarthritis |
| Asthma | | Cardiac asthma; Asthma; Asthma attack; Mixed asthma; Allergic asthma |
| Atherosclerosis | | Atherosclerosis; Cerebral atherosclerosis; Atherosclerosis of aorta; Coronary arteriosclerosis; Generalized atherosclerosis; Arteriosclerosis; Renal artery stenosis; Vertebral artery stenosis; Pulmonary artery stenosis; Internal carotid artery stenosis; Cerebral arteriosclerosis |
| Atrial fibrillation & flutter | | Atrial fibrillation; Paroxysmal atrial fibrillation; Atrial flutter; Atrial fibrillation and flutter; Persistent atrial fibrillation; Permanent atrial fibrillation; Paroxysmal atrial flutter; Chronic atrial fibrillation; History of atrial fibrillation |
| Cancer | | Multiple myeloma; Basal cell carcinoma; Lymphoma; Hodgkin disease; Chronic lymphocytic leukemia; Intrahepatic cholangiocarcinoma; Adenocarcinoma; Chronic myeloid leukemia; Acute myelocytic leukemia; Myeloid leukemia; Renal cell carcinoma; Adenocarcinoma of prostate; Malignant neoplasm of prostate; Merkel cell carcinoma; Papillary thyroid carcinoma; Leukemia; Malignant neoplasm of lung; Acute leukemia; Bronchioloalveolar adenocarcinoma; Acute lymphocytic leukemia; Breast carcinoma; Stomach carcinoma; Carcinoma; Liver carcinoma |
| Cardiomegaly | | Cardiomegaly |
| Cardiomyopathy | | Cardiomyopathies; Dilated cardiomyopathy; Hypertrophic cardiomyopathy; Ischemic cardiomyopathy; Hypertrophic cardiomyopathy without obstruction; Secondary dilated cardiomyopathy; Restrictive cardiomyopathy; Primary cardiomyopathies; Cardiomyopathy associated with another disorder |
| Carditis | | Myocarditis; Infective endocarditis; Pericarditis; Endocarditis; Subacute endocarditis; Subacute bacterial endocarditis; Acute myocarditis; Chronic myocarditis; Constrictive pericarditis; Carditis; Lymphocytic myocarditis; Viral myocarditis; Bacterial endocarditis |
| Cholecystolithiasis | | Cholelithiasis; Cholecystitis; Chronic cholecystitis; Cholecystolithiasis |
| Chronic kidney disease | | Chronic pyelonephritis; Chronic kidney diseases; Chronic kidney disease stage 3; Chronic kidney failure; Chronic kidney disease stage 2; Chronic kidney disease stage 4; Renal insufficiency; Chronic kidney disease stage 1; Chronic kidney disease stage 5 |
| Chronic obstructive pulmonary disease | | Chronic obstructive airway disease; Chronic bronchitis; Pulmonary emphysema; Emphysematous bronchitis; Chronic bullous emphysema; Subcutaneous emphysema; Paraseptal emphysema; Centriacinar emphysema; Panacinar emphysema; Severe chronic obstructive pulmonary disease |
| Congenital heart defects | | Congenital heart disease; Atrial septal defects; Ventricular septal defects; Congenital heart defects; Perimembranous ventricular septal defect; Aortopulmonary septal defect; Heart septal defects; Atrioventricular septal defect; Residual ventricular septal defect |
| Coronary artery disease | | Coronary heart disease; Myocardial ischemia; Chronic myocardial ischemia; Coronary arteriosclerosis; Coronary artery disease; Silent myocardial ischemia; Transient myocardial ischemia |
| Cough | | Coughing; Dry cough; Productive cough; Cough reflex; Paroxysmal cough; Persistent cough; Decreased coughing |
| Decompensation | | Decompensation; Decompensated chronic heart failure |
| Diabetes | | Non-insulin-dependent diabetes mellitus; Diabetes mellitus; Diabetic polyneuropathies; Diabetic nephropathy; Insulin-dependent diabetes mellitus; Nonproliferative diabetic retinopathy; Diabetic retinopathy; Diabetic foot; Complications of diabetes mellitus; Steroid-induced diabetes; Diabetes insipidus; History of diabetes mellitus type 2; Diabetic maculopathy; Gestational diabetes; Diabetic neuropathies; History of diabetes mellitus; Central diabetes insipidus |
| Diabetic complications | | Diabetic polyneuropathies; Diabetic foot; Complications of diabetes mellitus; Diabetic maculopathy; Diabetic neuropathies |
| Dyspnea | | Dyspnea; Dyspnea on exertion; Actual resting dyspnea; Paroxysmal dyspnea; Inspiratory dyspnea; Expiratory dyspnea; Paroxysmal nocturnal dyspnea |
| Edema | | Edema; Swelling; Peripheral edema; Pulmonary edema; Edema of lower extremity; Joint swelling; Angioedema; Edema of foot; Soft tissue swelling |
| Fatigue | | Fatigue; Tired; Quickly exhausted; Chronic fatigue syndrome |
| Gastritis | | Chronic gastritis; Gastritis; Erosive gastritis; Gastroduodenitis; Chronic erosive gastritis; Atrophic gastritis; Superficial gastritis; Chronic superficial gastritis; Chronic antral gastritis; Reflux gastritis |
| Gout | | Gout; Gouty arthritis; Gouty nephropathy; Chronic gouty arthritis; Primary gout |
| Heart failure | | Chronic heart failure; Heart failure; Left-sided heart failure; Biventricular congestive heart failure; Congestive heart failure; Decompensated chronic heart failure; Diastolic heart failure; Acute heart failure |
| Heart murmur | | Systolic murmurs; Heart murmur; Diastolic murmurs; Soft systolic murmur |
| Hernia | | Hernia; Hiatal hernia; Inguinal hernia; Umbilical hernia; Ventral hernia; Esophageal hernia; Diaphragmatic hernia; Herniated disk; Scrotal hernia; Sliding hernia; Bilateral inguinal hernia; Incisional hernia |
| Hyperlipidemia | | Dyslipidemias; Hyperlipidemia; Lipid metabolism disorders; Hypercholesterolemia; Hypertriglyceridemia; Familial hypercholesterolemia |
| Hypertension | | Hypertensive disease; Pulmonary hypertension; Hypertensive heart disease; History of hypertension; Idiopathic pulmonary arterial hypertension; Hypertensive crisis; Pulmonary arterial hypertension; Portal hypertension; Chronic thromboembolic pulmonary hypertension; Hypertensive encephalopathy; Secondary pulmonary hypertension; Essential hypertension; Systolic hypertension; White coat hypertension; Secondary hypertension |
| Left ventricular hypertrophy | | Left ventricular hypertrophy |
| Liver disease | | Chronic liver disease; Liver failure; Liver cirrhosis; Liver fibrosis; Liver damage; Fatty liver; Liver dysfunction |
| Lymphadenopathy | | Lymphadenopathy; Hilar lymphadenopathy; Mediastinal lymphadenopathy |
| Multiple organ failure | | Multiple organ failure |
| Myocardial infarction | | Myocardial infarction; Acute myocardial infarction; Subendocardial myocardial infarction; History of myocardial infarction; Postmyocardial infarction syndrome; Recent myocardial infarction |
| Obesity | | Obesity; Overweight; Morbid obesity; Abdominal obesity; Constitutional obesity |
| Paresis | | Hemiparesis; Left hemiparesis; Paraparesis; Spastic hemiplegia; Quadriparesis; Right hemiparesis |
| Pleural effusion | | Hydrothorax; Pleural effusion disorder; Bilateral pleural effusion |
| Proteinuria | | Proteinuria |
| Respiratory failure | | Respiratory failure |
| Rheumatic heart disease | | Chronic rheumatic heart disease; Rheumatic heart disease; Rheumatic mitral regurgitation; Rheumatic fever; Rheumatic aortic stenosis; Rheumatic aortic stenosis with regurgitation; Rheumatic disease of mitral valve |
| ST elevation | | ST segment elevation |
| Stroke | | Transient ischemic attack; Ischemic stroke; Cerebral infarction; Cerebral hemorrhage; Brain hemorrhage |
| Thromboembolism | | Thrombus; Thrombosis; Pulmonary embolism; Pulmonary thromboembolisms; Thromboembolism; Recurrent pulmonary embolism; Thrombophlebitis; Postthrombotic syndrome; Air embolism; Venous thrombosis; Deep vein thrombosis; Embolism; Old thrombus; Cerebral embolism; Non-occlusive thrombus; Deep thrombophlebitis |
| Thyroid disorders | | Autoimmune thyroiditis; Hypothyroidism; Subclinical hypothyroidism; Secondary hyperparathyroidism; Thyroid nodule; Papillary thyroid carcinoma; Hyperthyroidism; Hyperthyroidism secondary to amiodarone; Thyroid associated opthalmopathies; Hyperparathyroidism; Postoperative hypothyroidism; Toxic thyroid adenoma; Subclinical hyperthyroidism; Thyroiditis |
| Valve disease | | Mitral valve insufficiency; Tricuspid valve insufficiency; Aortic valve insufficiency; Aortic valve stenosis; Pulmonary valve insufficiency; Bicuspid aortic valve; Aortic valve disorder; Mitral valve stenosis; Heart valve disease; Mitral valve prolapse syndrome; Diseases of mitral valve; Mitral stenosis with insufficiency; Heart valve regurgitation; Aortic valve calcification; Pulmonary valve stenosis; Congenital atresia of pulmonary valve; Calcification of mitral valve |
| Vision disorders | | Cataract; Retinal diseases; Myopia; Presbyopia; Nonproliferative diabetic retinopathy; Diabetic retinopathy; Astigmatism; Macular degeneration; Myopic astigmatism; Proliferative retinopathy; Mixed astigmatism; Cataract secondary to ocular disorder; Corneal opacity; Age-related cataract; Severe myopia; Age related macular degeneration; Proliferative vitreoretinopathy; Central serous chorioretinopathy |

*Supplementary Table S3: Mapping of input features (complaints) into grouped complaints.*

|  | Encounter type | | | | |
| --- | --- | --- | --- | --- | --- |
| Cluster name | **Surgery/** **diagnostics** | **Inpatient** | **Ambulatory** | **Newborn/** **obstetrics** | **Dentistry** |
| ECG | 23.9847 | 50.5886 | 60.7122 | 0.5297 | 0.0000 |
| Cardiac surgery | 78.2167 | 99.9436 | 31.2077 | 0.0188 | 0.0376 |
| Atrial fibrillation | 36.7141 | 45.6807 | 75.7791 | 0.0000 | 0.0000 |
| General vascular disease | 46.0202 | 31.3131 | 82.9495 | 0.0000 | 0.0000 |
| Acute coronary syndrome | 61.9495 | 97.7040 | 35.5458 | 0.0417 | 0.0000 |
| Hypertrophic cardiomyopathy | 23.5361 | 31.5451 | 75.8972 | 0.6800 | 0.0000 |
| CAD, high-acuity | 69.7392 | 69.0267 | 58.0958 | 0.0000 | 0.0000 |
| Pediatric cardiomyopathy | 58.6946 | 81.0663 | 14.2501 | 1.8934 | 0.0000 |
| Aortic stenosis | 45.5185 | 45.0107 | 72.7788 | 0.0586 | 0.0391 |
| ECG w/ apnea | 13.7837 | 22.5924 | 85.4861 | 0.0000 | 0.0000 |
| CAD w/ myocardial ischemia | 30.9596 | 30.0883 | 81.2500 | 0.0000 | 0.0116 |
| Non-CV encounters | 15.1545 | 26.9829 | 78.5410 | 1.3252 | 0.0131 |
| NICU | 55.2608 | 86.0080 | 16.2025 | 10.6543 | 0.0000 |
| CAD w/ arteriosclerosis | 11.3797 | 7.9221 | 95.8375 | 0.0000 | 0.0000 |
| Dilated cardiomyopathy | 18.4550 | 29.2322 | 82.0146 | 0.0235 | 0.0000 |
| Aneurysm | 42.4306 | 54.6133 | 66.7961 | 0.0896 | 0.0000 |
| Echocardiography | 7.6093 | 7.1001 | 95.0869 | 0.0300 | 0.0000 |
| Cerebrovascular disease | 18.6821 | 54.0484 | 66.2110 | 0.0000 | 0.0000 |
| Congenital heart disease | 49.7452 | 53.7207 | 54.0775 | 1.1213 | 0.0000 |
| Advanced & decompensated HF | 29.5942 | 80.2363 | 50.8269 | 0.0719 | 0.0103 |
| Diabetic complications | 32.3613 | 50.7468 | 69.9502 | 0.0711 | 0.0711 |
| Lipid disorders | 9.7899 | 2.6159 | 99.4055 | 0.0000 | 0.0000 |
| CAD w/ cerebral involvement | 21.9258 | 19.7506 | 93.7935 | 0.0000 | 0.0000 |

*Supplementary Table S4: For each disease state cluster, percentage of data points (snapshots) that contain a healthcare encounter that can be categorized as surgery/diagnostics, inpatient, ambulatory, newborn/obstetrics, or dentistry.*

|  | **Service** | | | | | | | | | |
| --- | --- | --- | --- | --- | --- | --- | --- | --- | --- | --- |
| **Cluster name** | **ICU** | **Surgery** | **ECG** | **Echo** | **Angio-graphy** | **CABG** | **Valve repair & prosthesis** | **Defect correction** | **Aneurysm repair** | **Cardiac ablation** |
| ECG | 17.0394 | 19.0700 | 88.3755 | 65.1265 | 7.5338 | 4.0906 | 2.9429 | 0.3826 | 0.1177 | 4.2084 |
| Cardiac surgery | 99.2664 | 97.6862 | 95.4101 | 82.7126 | 49.4357 | 79.6840 | 23.2318 | 0.1693 | 1.5425 | 0.0564 |
| Atrial fibrillation | 19.1361 | 21.4325 | 48.7972 | 49.2346 | 4.2100 | 0.2734 | 0.3827 | 0.0000 | 0.1093 | 9.8961 |
| General vascular disease | 12.8889 | 15.1515 | 29.5354 | 39.9596 | 18.2626 | 3.9596 | 0.1616 | 0.0000 | 0.1212 | 0.0000 |
| Acute coronary syndrome | 52.0559 | 61.8451 | 88.2488 | 88.9793 | 63.2227 | 4.5293 | 0.4592 | 0.0000 | 0.1044 | 0.1670 |
| Hypertrophic cardiomyopathy | 10.2380 | 11.1069 | 39.4409 | 62.9392 | 9.8602 | 0.4156 | 0.4911 | 0.0000 | 0.0000 | 0.2645 |
| CAD, high-acuity | 30.2608 | 43.9964 | 37.7502 | 52.0619 | 36.5221 | 5.3669 | 0.1061 | 0.0000 | 0.0455 | 0.0455 |
| Pediatric cardiomyopathy | 2.9895 | 8.6198 | 83.8565 | 77.2297 | 6.0289 | 0.0000 | 0.0997 | 0.0997 | 0.0000 | 2.4913 |
| Aortic stenosis | 26.8502 | 28.2367 | 46.1238 | 66.3152 | 21.5778 | 9.2755 | 16.4812 | 0.0195 | 0.3515 | 0.1757 |
| ECG w/ apnea | 6.9831 | 7.0288 | 88.9092 | 63.3957 | 3.5600 | 2.0082 | 1.2323 | 0.0000 | 0.0913 | 0.4108 |
| CAD w/ myocardial ischemia | 11.4777 | 13.3132 | 28.8220 | 34.4447 | 8.6547 | 6.2500 | 0.2207 | 0.0000 | 0.0232 | 0.1510 |
| Non-CV encounters | 6.1536 | 7.9118 | 33.6286 | 35.6820 | 3.8509 | 0.9775 | 1.2006 | 0.1640 | 0.0525 | 0.3739 |
| NICU | 9.7701 | 67.1088 | 78.8462 | 22.9222 | 26.9673 | 0.9726 | 0.8621 | 30.1503 | 0.0884 | 0.0442 |
| CAD w/ arteriosclerosis | 1.8463 | 2.3498 | 12.2860 | 45.7872 | 1.6784 | 0.1678 | 0.0000 | 0.0000 | 0.0000 | 0.1007 |
| Dilated cardiomyopathy | 9.3449 | 10.9885 | 30.0775 | 52.6649 | 6.4334 | 0.1409 | 0.3052 | 0.0704 | 0.0235 | 0.8453 |
| Aneurysm | 25.6494 | 28.5160 | 47.5963 | 56.1959 | 24.4849 | 6.4198 | 1.7020 | 0.0597 | 4.2699 | 0.3583 |
| Echocardiography | 0.8089 | 0.5392 | 21.5698 | 99.0114 | 1.5279 | 0.0899 | 0.0899 | 0.0000 | 0.0000 | 0.0300 |
| Cerebrovascular disease | 17.1048 | 13.5647 | 57.6236 | 42.7971 | 12.5832 | 2.1030 | 0.7010 | 0.0351 | 0.3155 | 0.0000 |
| Congenital heart disease | 8.3588 | 41.5392 | 50.8665 | 48.1651 | 11.9266 | 1.8349 | 0.8155 | 20.6422 | 0.0000 | 0.3058 |
| Advanced & decompensated HF | 37.8942 | 30.3955 | 75.1823 | 78.2024 | 21.4278 | 3.8418 | 4.0267 | 0.0719 | 0.2157 | 1.9209 |
| Diabetic complications | 14.6871 | 17.7098 | 46.4083 | 39.4026 | 10.3129 | 3.3784 | 0.3556 | 0.0000 | 0.1067 | 0.1422 |
| Lipid disorders | 0.5945 | 0.6342 | 19.1439 | 39.4768 | 0.9116 | 0.1585 | 0.0793 | 0.0000 | 0.0000 | 0.0000 |
| CAD w/ cerebral involvement | 5.5684 | 7.2506 | 26.5661 | 36.0209 | 8.7007 | 1.4501 | 0.1740 | 0.0290 | 0.0290 | 0.1450 |

*Supplementary Table S5: For each disease state cluster, percentage of data points (snapshots) that contain a service that can be categorized as intensive care (ICU), surgery, electrocardiogram (ECG), echocardiography (echo), angiography, coronary artery bypass grafting (CABG), valve repair & prosthesis, heart defect correction, aneurysm repair, or cardiac ablation.*
