# Supplementary material for: Data-driven identification of heart failure disease states and progression pathways using electronic health records
Source: Sci Rep. 2022 Oct 25;12:17871. doi: 10.1038/s41598-022-22398-4 (PMC9596465; doi:10.1038/s41598-022-22398-4)
Supplement: Supplementary file 2 — Supplementary Information 2. [file 41598_2022_22398_MOESM2_ESM.pdf]

Cluster name	Concept	p-value
Acute coronary syndrome	Coronary heart disease	0
Acute coronary syndrome	Myocardial Ischemia	0
Acute coronary syndrome	Sinus rhythm	0
Acute coronary syndrome	Stenosis	0
Acute coronary syndrome	Blood flow	0
Acute coronary syndrome	Myocardial Infarction	0
Acute coronary syndrome	Cardiac Arrhythmia	0
Acute coronary syndrome	Pain	0
Acute coronary syndrome	Atrial Premature Complexes	0
Acute coronary syndrome	Unstable angina	0
Acute coronary syndrome	Systemic arterial pressure	0
Acute coronary syndrome	Acute Coronary Syndrome	0
Acute coronary syndrome	Wakefulness	0
Acute coronary syndrome	Acute myocardial infarction	0
Acute coronary syndrome	Akinesia	0
Acute coronary syndrome	Hepatitis B	0
Acute coronary syndrome	Chest Pain	0
Acute coronary syndrome	Peristalsis	0
Acute coronary syndrome	Peripheral edema	0
Acute coronary syndrome	Bradycardia	0
Acute coronary syndrome	Dysplasia	0
Acute coronary syndrome	Pain of sternum	0
Acute coronary syndrome	Coronary Stenosis	0
Acute coronary syndrome	Exercise-induced angina	0
Acute coronary syndrome	History of hypertension	0
Acute coronary syndrome	Respiratory Insufficiency	0
Acute coronary syndrome	Infarction	0
Acute coronary syndrome	Finding of blood glucose level	0
Acute coronary syndrome	Progressive Angina	0
Acute coronary syndrome	Cardiovascular Diseases	0
Acute coronary syndrome	ST segment elevation	0
Acute coronary syndrome	Left-Sided Heart Failure	0
Acute coronary syndrome	Finding of sodium level	0
Acute coronary syndrome	Subendocardial myocardial infarction	0
Acute coronary syndrome	Mitral valve area	3.938E-290
Acute coronary syndrome	Finding of creatinine level	8.5253E-289
Acute coronary syndrome	Supraventricular arrhythmia	2.8462E-260
Acute coronary syndrome	Stricture of artery	3.1846E-253
Acute coronary syndrome	Ventricular arrhythmia	7.7645E-253
Acute coronary syndrome	Urination	6.7902E-240
Acute coronary syndrome	Secondary pulmonary hypertension	1.3052E-228
Acute coronary syndrome	Restenosis	1.1663E-226
Acute coronary syndrome	Heart failure	3.8453E-223
Acute coronary syndrome	Angina Pectoris	3.9616E-222
Acute coronary syndrome	Urea level	1.0078E-220

Acute coronary syndrome	Cholesterol level test result	3.7494E-203
Acute coronary syndrome	Adams-Stokes Syndrome	6.0512E-202
Acute coronary syndrome	Pain, Burning	5.52E-199
Acute coronary syndrome	Finding of potassium level	1.2604E-189
Acute coronary syndrome	Glucose level	2.2581E-189
Acute coronary syndrome	Rest pain	2.0312E-188
Acute coronary syndrome	Vasodilation	4.4751E-187
Acute coronary syndrome	Hemostatic function	6.0341E-178
Acute coronary syndrome	Premature Cardiac Complex	1.5948E-166
Acute coronary syndrome	ST segment depression	6.8198E-160
Acute coronary syndrome	Aspartate transaminase level	7.6313E-160
Acute coronary syndrome	Hematoma	7.5303E-157
Acute coronary syndrome	Hypertensive heart disease	1.9017E-151
Acute coronary syndrome	Chronic bronchitis	2.3156E-146
Acute coronary syndrome	Blood group transfusion observation	4.923E-131
Acute coronary syndrome	Bilirubin level result	3.8077E-128
Acute coronary syndrome	ST segment	5.777E-115
Acute coronary syndrome	Radiographic contrast agent nephropathy	7.4822E-115
Acute coronary syndrome	Pulmonary Edema	4.77E-111
Acute coronary syndrome	Thrombosis	2.2853E-103
Acute coronary syndrome	Dyslipidemias	5.33498E-98
Acute coronary syndrome	Subcutaneous hematoma	1.43088E-97
Acute coronary syndrome	Albumin level	9.37562E-95
Acute coronary syndrome	Muscle Rigidity	2.47209E-94
Acute coronary syndrome	Slow shallow breathing	1.7089E-93
Acute coronary syndrome	Hypokinesia	3.87303E-93
Acute coronary syndrome	Premature ventricular contractions	1.98957E-90
Acute coronary syndrome	Color of urine	5.93258E-89
Acute coronary syndrome	Necrosis	1.87117E-86
Acute coronary syndrome	Cardiac rhythm type	1.56853E-84
Acute coronary syndrome	Hiatal Hernia	6.4586E-84
Acute coronary syndrome	Coronary Artery Disease	1.79678E-76
Acute coronary syndrome	Chronic gastritis	4.3226E-73
Acute coronary syndrome	Weakness	1.49758E-72
Acute coronary syndrome	Apnea	2.11684E-70
Acute coronary syndrome	Sinus bradycardia	2.18821E-70
Acute coronary syndrome	Hypertensive disease	1.70793E-67
Acute coronary syndrome	Wheezing	1.38043E-64
Acute coronary syndrome	Sclerosis	3.46862E-64
Acute coronary syndrome	Cardiogenic shock	3.83288E-63
Acute coronary syndrome	Sleeplessness	1.11916E-59
Acute coronary syndrome	Patent ductus arteriosus	1.77853E-57
Acute coronary syndrome	Cardiac pain	2.83485E-56
Acute coronary syndrome	Angina Pectoris, Variant	4.33045E-56
Acute coronary syndrome	Sinus Arrhythmia	5.23433E-56
Acute coronary syndrome	Parasystole	3.67208E-55

Acute coronary syndrome	Chest discomfort	8.55714E-55
Acute coronary syndrome	Sweating	1.72975E-54
Acute coronary syndrome	Cyanosis	1.83672E-53
Acute coronary syndrome	Surgical fistula	3.77068E-53
Acute coronary syndrome	Seizures	5.13404E-53
Acute coronary syndrome	Protein level	8.83025E-51
Acute coronary syndrome	Pseudoaneurysm	9.28405E-51
Acute coronary syndrome	Chronic myocardial ischemia	3.35669E-50
Acute coronary syndrome	Diastolic dysfunction	1.8228E-48
Advanced & decompensated HF	Dyspnea	0
Advanced & decompensated HF	Mitral Valve Insufficiency	0
Advanced & decompensated HF	Heart failure	0
Advanced & decompensated HF	Tricuspid Valve Insufficiency	0
Advanced & decompensated HF	Edema	0
Advanced & decompensated HF	Atrial Fibrillation	0
Advanced & decompensated HF	Weakness	0
Advanced & decompensated HF	Diuresis	0
Advanced & decompensated HF	Pulmonary Hypertension	0
Advanced & decompensated HF	Peripheral edema	0
Advanced & decompensated HF	Swelling	0
Advanced & decompensated HF	Decompensation	0
Advanced & decompensated HF	Ventricular Tachycardia	0
Advanced & decompensated HF	Effusion	0
Advanced & decompensated HF	Cardiac asthma	0
Advanced & decompensated HF	Hypotension	0
Advanced & decompensated HF	Ventricular arrhythmia	0
Advanced & decompensated HF	Hydrothorax	0
Advanced & decompensated HF	Coughing	0
Advanced & decompensated HF	Paroxysmal ventricular tachycardia	0
Advanced & decompensated HF	Pulmonary Embolism	0
Advanced & decompensated HF	Atrial Flutter	0
Advanced & decompensated HF	Ascites	0
Advanced & decompensated HF	Urination	0
Advanced & decompensated HF	Finding of creatinine level	0
Advanced & decompensated HF	Cardiomegaly	0
Advanced & decompensated HF	Pulmonary Thromboembolisms	0
Advanced & decompensated HF	Thromboembolism	0
Advanced & decompensated HF	Finding of blood glucose level	0
Advanced & decompensated HF	Hepatomegaly	0
Advanced & decompensated HF	Pulmonary Vascular Resistance	0
Advanced & decompensated HF	Dry cough	0
Advanced & decompensated HF	Inflammation	0
Advanced & decompensated HF	Noninflammatory pericardial Effusion	0
Advanced & decompensated HF	Orthotopic graft	0
Advanced & decompensated HF	Asphyxia	0
Advanced & decompensated HF	Multiple Organ Failure	0

Advanced & decompensated HF	Position of kidney	0
Advanced & decompensated HF	Kidney Failure	0
Advanced & decompensated HF	Hemoptysis	0
Advanced & decompensated HF	Azotemia	0
Advanced & decompensated HF	Recurrent pulmonary embolism	0
Advanced & decompensated HF	Anasarca	0
Advanced & decompensated HF	Actual Resting Dyspnea	0
Advanced & decompensated HF	Orthopnea	0
Advanced & decompensated HF	Biventricular congestive heart failure	0
Advanced & decompensated HF	Bilateral pneumonia	0
Advanced & decompensated HF	Cachexia	0
Advanced & decompensated HF	Acidosis	0
Advanced & decompensated HF	Renal Insufficiency	0
Advanced & decompensated HF	Liver Failure	0
Advanced & decompensated HF	Hypoalbuminemia	0
Advanced & decompensated HF	Hypoproteinemia	0
Advanced & decompensated HF	Hyponatremia	0
Advanced & decompensated HF	Oliguria	0
Advanced & decompensated HF	Hypovolemia	0
Advanced & decompensated HF	Catheter related infection	0
Advanced & decompensated HF	Chills	9.6574E-297
Advanced & decompensated HF	Hypokalemia	5.8425E-294
Advanced & decompensated HF	Pleural Diseases	6.9976E-289
Advanced & decompensated HF	Leukocytosis	9.1188E-284
Advanced & decompensated HF	Thrombus	1.9023E-283
Advanced & decompensated HF	Leukopenia	3.3111E-279
Advanced & decompensated HF	Finding of creatine kinase level	4.6079E-277
Advanced & decompensated HF	Respiratory Failure	3.2948E-273
Advanced & decompensated HF	Nosocomial pneumonia	6.4914E-264
Advanced & decompensated HF	Finding of acid-base balance	4.1323E-262
Advanced & decompensated HF	Permanent atrial fibrillation	2.939E-253
Advanced & decompensated HF	Finding of sodium level	3.0149E-251
Advanced & decompensated HF	Viral respiratory infection	9.8841E-251
Advanced & decompensated HF	Right ventricular failure	5.5744E-242
Advanced & decompensated HF	Lymphadenopathy	5.6379E-241
Advanced & decompensated HF	Congestion	6.1723E-239
Advanced & decompensated HF	Premature ventricular contractions	2.1945E-238
Advanced & decompensated HF	Pathological Dilatation	3.7764E-237
Advanced & decompensated HF	Infection of bloodstream	4.1195E-236
Advanced & decompensated HF	Hyperhomocysteinemia	1.3325E-233
Advanced & decompensated HF	Choking	2.0604E-223
Advanced & decompensated HF	Sudden Cardiac Death	2.8414E-222
Advanced & decompensated HF	Hepatic Insufficiency	1.3377E-220
Advanced & decompensated HF	Respiratory Insufficiency	4.1678E-215
Advanced & decompensated HF	Wakefulness	5.648E-211
Advanced & decompensated HF	Tachycardia	3.747E-209

Advanced & decompensated HF	Aortopulmonary Septal Defect	5.4273E-209
Advanced & decompensated HF	Hyperkalemia	1.8673E-200
Advanced & decompensated HF	Paroxysmal dyspnea	8.5291E-200
Advanced & decompensated HF	Gouty arthritis	8.9604E-198
Advanced & decompensated HF	Lethargy	1.1459E-197
Advanced & decompensated HF	Invasive arterial pressure	4.6343E-197
Advanced & decompensated HF	Hematoma	7.4985E-196
Advanced & decompensated HF	Finding of potassium level	2.1538E-195
Advanced & decompensated HF	Asthma attack	2.422E-188
Advanced & decompensated HF	Acrocyanosis	1.2878E-185
Advanced & decompensated HF	Weight Gain	6.8493E-184
Advanced & decompensated HF	Heart valve regurgitation	7.9765E-184
Advanced & decompensated HF	Metabolic Inhibition	1.9059E-182
Advanced & decompensated HF	Steroid-induced diabetes	7.7738E-178
Advanced & decompensated HF	Hepatoptosis	3.5177E-174
Advanced & decompensated HF	Chronic Kidney Diseases	3.7572E-174
Advanced & decompensated HF	Chronic heart failure	2.2638E-173
Aneurysm	Aneurysm	0
Aneurysm	Myocardial Infarction	0
Aneurysm	Thrombus	0
Aneurysm	Left ventricular aneurysm	0
Aneurysm	Dissection of aorta	0
Aneurysm	Aortic Aneurysm	0
Aneurysm	Abdominal Aortic Aneurysm	0
Aneurysm	Cardiac dyskinesia	1.1976E-303
Aneurysm	Akinesia	4.5726E-232
Aneurysm	Atherosclerosis of aorta	2.9048E-182
Aneurysm	Aneurysm of ascending aorta	1.8241E-135
Aneurysm	Old thrombus	5.7903E-117
Aneurysm	Myocardial Ischemia	5.79663E-95
Aneurysm	Coronary heart disease	8.33533E-90
Aneurysm	Atherosclerosis	3.26496E-83
Aneurysm	Saccular Aneurysm	1.47611E-62
Aneurysm	Pathological Dilatation	3.94682E-53
Aneurysm	Chronic Hepatitis C	3.75377E-52
Aneurysm	Prostatic Hyperplasia	5.64092E-52
Aneurysm	Aortic Diseases	1.12783E-46
Aneurysm	Chronic gastritis	4.794E-45
Aneurysm	Circulatory arrest	8.01442E-45
Aneurysm	Suppression	7.23861E-44
Aneurysm	Generalized atherosclerosis	3.42049E-41
Aneurysm	Ischemic cardiomyopathy	4.04281E-41
Aneurysm	Ulceration	1.58762E-39
Aneurysm	Acute myocardial infarction	5.21206E-39
Aneurysm	Angina Pectoris	5.5305E-39
Aneurysm	Aortic Valve Insufficiency	8.87108E-39

Aneurysm	Chronic heart failure	5.38548E-34
Aneurysm	Dyslipidemias	1.60285E-33
Aneurysm	Thrombosis	5.07871E-33
Aneurysm	Mitral Valve Insufficiency	1.13645E-31
Aneurysm	Arterial insufficiency	5.21919E-31
Aneurysm	Ventricular Tachycardia	9.93067E-29
Aneurysm	Peptic Ulcer	1.93966E-28
Aneurysm	Chronic Obstructive Airway Disease	3.03718E-28
Aneurysm	Rupture	2.90786E-25
Aneurysm	Dupuytren Contracture	7.22922E-25
Aneurysm	Chronic myocardial ischemia	1.50151E-23
Aneurysm	Dysfunction of papillary muscle	3.16503E-18
Aneurysm	Hypertensive disease	1.72145E-17
Aneurysm	Chronic antral gastritis	7.3712E-17
Aneurysm	Subarachnoid Hemorrhage	8.1478E-16
Aneurysm	Pulmonary Edema	3.87208E-15
Aneurysm	Surgical fistula	2.21305E-14
Aneurysm	Sudden Cardiac Death	2.36237E-14
Aneurysm	Coronary Stenosis	1.47731E-13
Aneurysm	Cyst	5.69652E-13
Aneurysm	Duodenal Ulcer	8.39669E-13
Aneurysm	Stenosis	3.86609E-12
Aneurysm	Chronic glomerulonephritis	1.02371E-11
Aneurysm	Dysplasia	2.03696E-11
Aneurysm	Systolic dysfunction	2.83603E-11
Aneurysm	Gastric hemorrhage	2.88479E-11
Aneurysm	Colonic Polyps	2.33775E-10
Aneurysm	Pseudoaneurysm	5.97171E-10
Aneurysm	Chronic pyelonephritis	1.66452E-09
Aneurysm	History of myocardial infarction	6.18433E-09
Aortic stenosis	Aortic Valve Stenosis	0
Aortic stenosis	Stenosis	0
Aortic stenosis	Aortic Valve Insufficiency	0
Aortic stenosis	Calcification	0
Aortic stenosis	Heart Diseases	0
Aortic stenosis	Heart valve disease	0
Aortic stenosis	Heart Neoplasm	0
Aortic stenosis	Aortic valve disorder	0
Aortic stenosis	Bicuspid aortic valve	0
Aortic stenosis	Atherosclerosis of aorta	0
Aortic stenosis	Chronic rheumatic heart disease	0
Aortic stenosis	Mitral Valve Stenosis	0
Aortic stenosis	Aortic valve area	0
Aortic stenosis	Aortic valve calcification	0
Aortic stenosis	Fibrous ring	4.9228E-278
Aortic stenosis	Heart murmur	5.2149E-189

Aortic stenosis	Idiopathic pulmonary arterial hypertension	2.7238E-156
Aortic stenosis	Infective endocarditis	1.0338E-144
Aortic stenosis	Pulmonary Hypertension	1.9436E-143
Aortic stenosis	Cerebrovascular Disorders	4.626E-139
Aortic stenosis	Aortic Diseases	6.6961E-134
Aortic stenosis	Left Ventricular Hypertrophy	1.5621E-132
Aortic stenosis	Mitral Valve Insufficiency	1.454E-126
Aortic stenosis	Congenital heart disease	3.9814E-108
Aortic stenosis	Concentric hypertrophy	4.4449E-107
Aortic stenosis	Arrhythmogenic Right Ventricular Dysplasia	1.1783E-100
Aortic stenosis	Congenital Heart Defects	1.4976E-100
Aortic stenosis	Fibrosis	8.83306E-95
Aortic stenosis	Mitral stenosis with insufficiency	2.99819E-68
Aortic stenosis	Subacute endocarditis	4.97596E-58
Aortic stenosis	Blood flow	6.13284E-57
Aortic stenosis	Postpericardiotomy Syndrome	2.08232E-55
Aortic stenosis	Encephalopathies	5.69999E-54
Aortic stenosis	Systolic Murmurs	2.66417E-53
Aortic stenosis	Tricuspid Valve Insufficiency	1.29048E-51
Aortic stenosis	Heart murmur quality, blowing	6.08651E-51
Aortic stenosis	Chronic heart failure	1.10404E-47
Aortic stenosis	Rheumatism	2.90999E-47
Aortic stenosis	Respiratory Distress Syndrome	1.33678E-37
Aortic stenosis	Hodgkin Disease	1.22665E-36
Aortic stenosis	Hypertensive disease	5.56417E-35
Aortic stenosis	Chronic gastritis	4.2371E-33
Aortic stenosis	Atherosclerosis	9.10876E-29
Aortic stenosis	Rheumatic Heart Disease	3.07342E-26
Aortic stenosis	Paroxysmal atrial fibrillation	2.55097E-25
Aortic stenosis	Hyperlipidemia	3.08205E-25
Aortic stenosis	Cholelithiasis	2.48118E-23
Aortic stenosis	Osteogenesis	1.69681E-22
Aortic stenosis	Nodular Goiter	1.936E-21
Aortic stenosis	Subacute Bacterial Endocarditis	2.47442E-20
Aortic stenosis	Atrophic Vaginitis	5.46326E-20
Aortic stenosis	Atrophic	1.86255E-18
Aortic stenosis	Cerebral Atherosclerosis	1.09584E-17
Aortic stenosis	Pulmonary Valve Insufficiency	3.76202E-17
Aortic stenosis	Varicosity	4.23208E-17
Aortic stenosis	Hypochromic anemia	7.84728E-17
Aortic stenosis	Diseases of mitral valve	2.07971E-16
Aortic stenosis	Syncope	6.46532E-16
Aortic stenosis	Hypertensive heart disease	9.98725E-16
Aortic stenosis	Effusion	1.00303E-15
Aortic stenosis	Dyspnea	8.77798E-15
Aortic stenosis	Anemia	1.19245E-14

Aortic stenosis	Diastolic dysfunction	2.11964E-14
Aortic stenosis	Mitral restenosis	3.27081E-14
Aortic stenosis	Atrophic Gastritis	4.24638E-14
Aortic stenosis	Diastolic Murmurs	1.4743E-13
Aortic stenosis	Chronic anemia	1.65631E-13
Aortic stenosis	Cerebrovascular accident	4.17194E-13
Aortic stenosis	Sensorineural Hearing Loss	1.00992E-12
Aortic stenosis	Generalized atherosclerosis	1.08398E-12
Aortic stenosis	Hypercholesterolemia	1.85889E-12
Aortic stenosis	Soft systolic murmur	3.14193E-12
Aortic stenosis	Cyst	6.84399E-12
Aortic stenosis	Prostatic Hyperplasia	5.71761E-11
Aortic stenosis	Lesion	1.07824E-10
Aortic stenosis	Atrial standstill	1.35839E-10
Aortic stenosis	Liver cyst	2.06863E-10
Aortic stenosis	Cardiac activity	7.7703E-10
Atrial fibrillation	Atrial fibrillation and flutter	0
Atrial fibrillation	Atrial Fibrillation	0
Atrial fibrillation	Paroxysmal atrial fibrillation	0
Atrial fibrillation	Atrial Flutter	0
Atrial fibrillation	Persistent atrial fibrillation	0
Atrial fibrillation	Under local anesthesia	0
Atrial fibrillation	Irregular heart beat	0
Atrial fibrillation	Ablation frequency	0
Atrial fibrillation	Heart beat	1.9675E-166
Atrial fibrillation	Palpitations	1.5621E-149
Atrial fibrillation	Post-op diagnosis	2.7411E-139
Atrial fibrillation	Fibrillation	2.2685E-124
Atrial fibrillation	Cardiac rhythm type	6.5493E-119
Atrial fibrillation	Chronic heart failure	2.1294E-116
Atrial fibrillation	Cardiac conduction	1.7868E-108
Atrial fibrillation	Nodular Goiter	2.4235E-99
Atrial fibrillation	Permanent atrial fibrillation	1.90643E-97
Atrial fibrillation	Paroxysmal atrial flutter	4.29505E-87
Atrial fibrillation	Tachyarrhythmia	1.74574E-69
Atrial fibrillation	Obesity	1.83419E-69
Atrial fibrillation	Premature ventricular contractions	2.4992E-64
Atrial fibrillation	Thyrotoxicosis	1.22218E-58
Atrial fibrillation	Varicosity	1.6114E-55
Atrial fibrillation	Hypertensive disease	1.65524E-55
Atrial fibrillation	Cardiac Arrhythmia	2.69408E-54
Atrial fibrillation	Thrombus	1.42191E-49
Atrial fibrillation	Dyspnea	2.78009E-49
Atrial fibrillation	Subclinical hypothyroidism	5.48578E-47
Atrial fibrillation	Hypothyroidism	1.81829E-46
Atrial fibrillation	Sick Sinus Syndrome	2.47425E-44

Atrial fibrillation	Tachycardia	2.17905E-40
Atrial fibrillation	Edema	1.0184E-39
Atrial fibrillation	Peripheral edema	1.83605E-34
Atrial fibrillation	Hyperthyroidism secondary to amiodarone	2.25941E-27
Atrial fibrillation	Ventricular Tachycardia	2.90348E-26
Atrial fibrillation	Secondary dilated cardiomyopathy	3.62555E-23
Atrial fibrillation	Mean blood pressure	1.77749E-20
Atrial fibrillation	Gastrointestinal Hemorrhage	1.61362E-19
Atrial fibrillation	Atrial dilatation	2.09474E-19
Atrial fibrillation	Supraventricular arrhythmia	2.71941E-19
Atrial fibrillation	Monomorphic ventricular tachycardia	1.63904E-17
Atrial fibrillation	Hyperlipidemia	4.20591E-16
Atrial fibrillation	Nontoxic goiter	1.34213E-15
Atrial fibrillation	Dizziness	1.00195E-14
Atrial fibrillation	Hypertensive heart disease	1.66352E-14
Atrial fibrillation	Autoimmune thyroiditis	5.36231E-14
Atrial fibrillation	Impaired glucose tolerance	8.09444E-14
Atrial fibrillation	Weakness	3.06377E-13
Atrial fibrillation	Paroxysmal ventricular tachycardia	1.41439E-12
Atrial fibrillation	Chronic rheumatic heart disease	2.27207E-12
Atrial fibrillation	Chronic venous insufficiency	3.6993E-12
Atrial fibrillation	Bradycardia	7.92121E-12
Atrial fibrillation	Premature Cardiac Complex	1.39856E-11
Atrial fibrillation	Cardiac Arrest	1.53235E-10
Atrial fibrillation	Posttransfusion purpura	3.02643E-10
Atrial fibrillation	Cholelithiasis	5.30398E-10
Atrial fibrillation	Chronic pancreatitis	2.71859E-09
Atrial fibrillation	Cardiovascular finding	8.50253E-09
CAD w/ arteriosclerosis	Coronary Arteriosclerosis	0
CAD w/ arteriosclerosis	Chronic heart failure	0
CAD w/ arteriosclerosis	Hernia	0
CAD w/ arteriosclerosis	Esophageal Diseases	0
CAD w/ arteriosclerosis	Interventricular dyssynchrony	0
CAD w/ arteriosclerosis	Splenomegaly	1.8756E-216
CAD w/ arteriosclerosis	Hyperuricemia	2.6297E-138
CAD w/ arteriosclerosis	Cardiac dilatation	2.714E-118
CAD w/ arteriosclerosis	Coronary heart disease	7.3546E-110
CAD w/ arteriosclerosis	Cardiomegaly	8.4063E-101
CAD w/ arteriosclerosis	Atherosclerosis	9.5553E-100
CAD w/ arteriosclerosis	Nephrosclerosis	5.70157E-98
CAD w/ arteriosclerosis	Chronic Kidney Diseases	5.73438E-76
CAD w/ arteriosclerosis	Dyslipidemias	5.03169E-75
CAD w/ arteriosclerosis	Hypertensive disease	7.17177E-74
CAD w/ arteriosclerosis	Chronic liver disease	3.66207E-71
CAD w/ arteriosclerosis	Premature Cardiac Complex	3.06745E-65
CAD w/ arteriosclerosis	Varicosity	8.7203E-65

CAD w/ arteriosclerosis	Fever	8.9383E-53
CAD w/ arteriosclerosis	Cholelithiasis	4.0584E-50
CAD w/ arteriosclerosis	Ischemic cardiomyopathy	4.8013E-45
CAD w/ arteriosclerosis	Chronic Obstructive Airway Disease	1.21504E-44
CAD w/ arteriosclerosis	Obesity	2.13724E-41
CAD w/ arteriosclerosis	Chronic kidney disease stage 2	4.43359E-34
CAD w/ arteriosclerosis	Overweight	3.96021E-33
CAD w/ arteriosclerosis	Hiatal Hernia	8.97318E-33
CAD w/ arteriosclerosis	Cardiovascular finding	1.30624E-31
CAD w/ arteriosclerosis	Paroxysmal ventricular tachycardia	1.45885E-30
CAD w/ arteriosclerosis	Premature Cardiac Complex	6.40624E-30
CAD w/ arteriosclerosis	Gout	3.86486E-28
CAD w/ arteriosclerosis	Myocardial Ischemia	2.04984E-27
CAD w/ arteriosclerosis	Atrial Fibrillation	3.8326E-26
CAD w/ arteriosclerosis	Chronic bullous emphysema	2.29118E-23
CAD w/ arteriosclerosis	Chronic kidney disease stage 3	9.23285E-22
CAD w/ arteriosclerosis	Prostatic Hyperplasia	5.54033E-20
CAD w/ arteriosclerosis	Cyst	1.81182E-16
CAD w/ arteriosclerosis	Psoriasis	2.60726E-16
CAD w/ arteriosclerosis	Hypothyroidism	7.38005E-15
CAD w/ arteriosclerosis	Cholecystitis	7.69307E-14
CAD w/ arteriosclerosis	Cerebrovascular Disorders	1.31138E-13
CAD w/ arteriosclerosis	Hemangioma of liver	1.43914E-13
CAD w/ arteriosclerosis	Ventricular Tachycardia	1.79947E-13
CAD w/ arteriosclerosis	Chronic cystitis	6.8085E-13
CAD w/ arteriosclerosis	Non-Insulin-Dependent Diabetes Mellitus	2.42998E-12
CAD w/ arteriosclerosis	Cavernous Hemangioma of Brain	2.89146E-12
CAD w/ arteriosclerosis	Esophageal Hernia	4.64225E-12
CAD w/ arteriosclerosis	Subclinical hypothyroidism	5.22841E-12
CAD w/ arteriosclerosis	Hyperlipidemia	2.0269E-11
CAD w/ arteriosclerosis	Irritable Bowel Syndrome	2.08241E-11
CAD w/ arteriosclerosis	Mitral Valve Insufficiency	6.98989E-11
CAD w/ arteriosclerosis	Chronic pyelonephritis	3.42822E-10
CAD w/ arteriosclerosis	Chronic kidney disease stage 4	3.44336E-10
CAD w/ arteriosclerosis	Pyelonephritis	1.31784E-09
CAD w/ arteriosclerosis	Myocardial Infarction	1.42357E-09
CAD w/ arteriosclerosis	Nodular Goiter	4.05446E-09
CAD w/ arteriosclerosis	Iron deficiency anemia	5.05676E-09
CAD w/ cerebral involvement	Pain	0
CAD w/ cerebral involvement	Lesion	0
CAD w/ cerebral involvement	Vertebrobasilar Insufficiency	0
CAD w/ cerebral involvement	Hypertensive disease	0
CAD w/ cerebral involvement	Hyperlipidemia	0
CAD w/ cerebral involvement	Cerebrovascular Disorders	0
CAD w/ cerebral involvement	Varicosity	0
CAD w/ cerebral involvement	Cerebral Atherosclerosis	0

CAD w/ cerebral involvement	Pyelonephritis	0
CAD w/ cerebral involvement	Cholecystitis	0
CAD w/ cerebral involvement	Cerebral Arteriosclerosis	0
CAD w/ cerebral involvement	Coronary heart disease	3.2867E-274
CAD w/ cerebral involvement	Gastritis	3.6253E-251
CAD w/ cerebral involvement	Coronary Arteriosclerosis	2.0041E-231
CAD w/ cerebral involvement	Fever	6.7921E-200
CAD w/ cerebral involvement	Myalgia	2.0771E-195
CAD w/ cerebral involvement	Tinnitus	1.1356E-194
CAD w/ cerebral involvement	Chronic heart failure	3.0356E-192
CAD w/ cerebral involvement	Vomiting	1.0067E-177
CAD w/ cerebral involvement	Chronic liver disease	3.4235E-175
CAD w/ cerebral involvement	Heart Diseases	1.8858E-173
CAD w/ cerebral involvement	Nausea	2.4193E-148
CAD w/ cerebral involvement	Nodular Goiter	2.5443E-138
CAD w/ cerebral involvement	Cholelithiasis	9.65E-119
CAD w/ cerebral involvement	Angina Pectoris	3.1747E-104
CAD w/ cerebral involvement	Vertigo	2.25407E-98
CAD w/ cerebral involvement	Osteochondrosis	8.54739E-95
CAD w/ cerebral involvement	Non-Insulin-Dependent Diabetes Mellitus	4.3787E-86
CAD w/ cerebral involvement	Obesity	1.77712E-76
CAD w/ cerebral involvement	Atherosclerosis	3.88372E-73
CAD w/ cerebral involvement	Paroxysmal supraventricular tachycardia	1.42763E-67
CAD w/ cerebral involvement	Increase in blood pressure	2.1088E-65
CAD w/ cerebral involvement	Chronic cholecystitis	3.51232E-56
CAD w/ cerebral involvement	Dystonia Disorders	4.12281E-56
CAD w/ cerebral involvement	Cardiomyopathies	2.70399E-54
CAD w/ cerebral involvement	Dizziness	3.06759E-44
CAD w/ cerebral involvement	Mean blood pressure	2.96744E-42
CAD w/ cerebral involvement	Chronic Kidney Failure	3.43964E-40
CAD w/ cerebral involvement	Cardiovascular finding	1.19098E-39
CAD w/ cerebral involvement	Palpitations	3.1371E-36
CAD w/ cerebral involvement	Duodenal Ulcer	2.67507E-35
CAD w/ cerebral involvement	Encephalopathies	1.34569E-34
CAD w/ cerebral involvement	Menopause	1.79048E-33
CAD w/ cerebral involvement	Familial hypercholesterolemia	2.89595E-32
CAD w/ cerebral involvement	Pain of sternum	2.31467E-27
CAD w/ cerebral involvement	Heart failure	1.35242E-26
CAD w/ cerebral involvement	Overweight	8.20146E-26
CAD w/ cerebral involvement	Dyspnea	1.31113E-23
CAD w/ cerebral involvement	Chronic Obstructive Airway Disease	7.25838E-22
CAD w/ cerebral involvement	Myocardial Ischemia	3.56661E-20
CAD w/ cerebral involvement	Coarctation	6.41741E-19
CAD w/ cerebral involvement	Pancreatitis	9.80859E-19
CAD w/ cerebral involvement	Chronic myocardial ischemia	1.64303E-18
CAD w/ cerebral involvement	Autoimmune thyroiditis	6.80158E-18

CAD w/ cerebral involvement	Glaucoma	6.07017E-15
CAD w/ cerebral involvement	Bronchitis	1.45377E-13
CAD w/ cerebral involvement	Myopia	2.56149E-12
CAD w/ cerebral involvement	Spasm	3.41338E-12
CAD w/ cerebral involvement	Gastroesophageal reflux disease	3.54772E-12
CAD w/ cerebral involvement	Ache	7.96064E-09
CAD w/ myocardial ischemia	Hypertensive disease	0
CAD w/ myocardial ischemia	Coronary heart disease	0
CAD w/ myocardial ischemia	Chronic heart failure	0
CAD w/ myocardial ischemia	Angina Pectoris	0
CAD w/ myocardial ischemia	Myocardial Infarction	0
CAD w/ myocardial ischemia	Atherosclerosis	0
CAD w/ myocardial ischemia	Myocardial Ischemia	0
CAD w/ myocardial ischemia	Dyslipidemias	0
CAD w/ myocardial ischemia	Cerebrovascular Disorders	0
CAD w/ myocardial ischemia	Non-Insulin-Dependent Diabetes Mellitus	0
CAD w/ myocardial ischemia	Hyperlipidemia	0
CAD w/ myocardial ischemia	Duodenal Ulcer	3.9857E-304
CAD w/ myocardial ischemia	Encephalopathies	3.6669E-268
CAD w/ myocardial ischemia	Obesity	8.9016E-207
CAD w/ myocardial ischemia	Prostatic Hyperplasia	9.3722E-207
CAD w/ myocardial ischemia	Chronic pyelonephritis	3.5607E-203
CAD w/ myocardial ischemia	Cholelithiasis	2.782E-173
CAD w/ myocardial ischemia	Peptic Ulcer	1.9477E-166
CAD w/ myocardial ischemia	Coronary Artery Disease	2.4732E-147
CAD w/ myocardial ischemia	Chronic Obstructive Airway Disease	4.5111E-138
CAD w/ myocardial ischemia	Varicosity	3.2592E-132
CAD w/ myocardial ischemia	Pyelonephritis	1.9373E-128
CAD w/ myocardial ischemia	Chronic liver disease	7.3687E-99
CAD w/ myocardial ischemia	Nodular Goiter	9.29077E-92
CAD w/ myocardial ischemia	Chronic myocardial ischemia	6.44006E-87
CAD w/ myocardial ischemia	Pain	2.643E-82
CAD w/ myocardial ischemia	Generalized atherosclerosis	1.12502E-79
CAD w/ myocardial ischemia	Pain of sternum	2.05354E-75
CAD w/ myocardial ischemia	Cholecystitis	1.91979E-68
CAD w/ myocardial ischemia	Acute myocardial infarction	5.27292E-54
CAD w/ myocardial ischemia	Spinal Cord Injuries	2.10075E-53
CAD w/ myocardial ischemia	Overweight	2.0949E-47
CAD w/ myocardial ischemia	Arterial insufficiency	3.21599E-46
CAD w/ myocardial ischemia	Spinal Diseases	4.89668E-46
CAD w/ myocardial ischemia	Postpericardiotomy Syndrome	3.20249E-39
CAD w/ myocardial ischemia	Chest Pain	1.14822E-37
CAD w/ myocardial ischemia	Fever	9.31504E-37
CAD w/ myocardial ischemia	Spasm	1.19354E-35
CAD w/ myocardial ischemia	Chronic gastritis	2.2261E-34
CAD w/ myocardial ischemia	Exercise-induced angina	6.44228E-33

CAD w/ myocardial ischemia	Chronic cholecystitis	1.26053E-31
CAD w/ myocardial ischemia	Angina Pectoris, Variant	6.45459E-31
CAD w/ myocardial ischemia	Hypertriglyceridemia	7.25939E-31
CAD w/ myocardial ischemia	Paroxysmal atrial fibrillation	1.9834E-29
CAD w/ myocardial ischemia	Impaired glucose tolerance	2.85685E-27
CAD w/ myocardial ischemia	Ischemia	6.07015E-25
CAD w/ myocardial ischemia	Asthma	1.04051E-23
CAD w/ myocardial ischemia	Lesion	1.26495E-23
CAD w/ myocardial ischemia	Cerebral Atherosclerosis	1.07283E-21
CAD w/ myocardial ischemia	Hypercholesterolemia	1.35527E-18
CAD w/ myocardial ischemia	Degenerative polyarthritis	6.40068E-18
CAD w/ myocardial ischemia	Ulcer	1.68622E-17
CAD w/ myocardial ischemia	Cholecystolithiasis	7.90037E-16
CAD w/ myocardial ischemia	Chronic pancreatitis	1.71653E-15
CAD w/ myocardial ischemia	Gastric ulcer	2.87646E-15
CAD w/ myocardial ischemia	Chronic erosive gastritis	2.01626E-14
CAD w/ myocardial ischemia	Intermittent Claudication	8.97801E-14
CAD w/ myocardial ischemia	Prostatic Adenoma	4.36665E-13
CAD w/ myocardial ischemia	Chronic bronchitis	8.11594E-13
CAD w/ myocardial ischemia	Sick Sinus Syndrome	1.52227E-12
CAD w/ myocardial ischemia	Cardiovascular finding	1.80355E-12
CAD w/ myocardial ischemia	Erosive gastritis	4.75886E-12
CAD w/ myocardial ischemia	Chronic glomerulonephritis	9.28718E-12
CAD w/ myocardial ischemia	Hypertensive heart disease	1.45608E-11
CAD w/ myocardial ischemia	Chronic venous insufficiency	2.04024E-11
CAD w/ myocardial ischemia	Atherosclerosis of aorta	2.65959E-10
CAD w/ myocardial ischemia	Hyperesthesia	3.03657E-10
CAD w/ myocardial ischemia	Right coronary artery occlusion	3.81139E-09
CAD, high acuity	Coronary heart disease	0
CAD, high acuity	Hypertensive disease	0
CAD, high acuity	Angina Pectoris	0
CAD, high acuity	Myocardial Infarction	0
CAD, high acuity	Myocardial Ischemia	0
CAD, high acuity	Stenosis	0
CAD, high acuity	Heart failure	0
CAD, high acuity	Hypokinesia	0
CAD, high acuity	Atherosclerosis	0
CAD, high acuity	Chronic gastritis	0
CAD, high acuity	Akinesia	0
CAD, high acuity	Systemic arterial pressure	0
CAD, high acuity	Exercise-induced angina	0
CAD, high acuity	Chronic myocardial ischemia	0
CAD, high acuity	Dysplasia	0
CAD, high acuity	Gastritis	0
CAD, high acuity	Hemostatic function	0
CAD, high acuity	Acute myocardial infarction	0

CAD, high acuity	Coronary Stenosis	0
CAD, high acuity	Pain of sternum	0
CAD, high acuity	History of hypertension	0
CAD, high acuity	Cardiovascular Diseases	0
CAD, high acuity	Stricture of artery	0
CAD, high acuity	Restenosis	0
CAD, high acuity	Hypertensive heart disease	2.7642E-294
CAD, high acuity	Spontaneous respiration	7.7722E-294
CAD, high acuity	Cardiac dyskinesia	1.6193E-246
CAD, high acuity	Peripheral edema	1.3327E-245
CAD, high acuity	Hiatal Hernia	1.8118E-245
CAD, high acuity	Chest Pain	1.4988E-208
CAD, high acuity	Chronic liver disease	4.098E-190
CAD, high acuity	Hepatitis B	3.8418E-188
CAD, high acuity	Fever	2.1695E-183
CAD, high acuity	ST segment depression	3.2164E-170
CAD, high acuity	Transient ischemia	2.3176E-169
CAD, high acuity	Cholesterol level test result	6.603E-155
CAD, high acuity	Cardiovascular finding	1.3135E-143
CAD, high acuity	Seizures	3.4313E-135
CAD, high acuity	Blood group transfusion observation	3.732E-128
CAD, high acuity	Coronary Artery Disease	5.6794E-127
CAD, high acuity	Peristalsis	3.2894E-111
CAD, high acuity	Surgical fistula	1.7568E-107
CAD, high acuity	Ischemia	1.7197E-104
CAD, high acuity	Spinal Diseases	3.056E-99
CAD, high acuity	Pain	1.42074E-91
CAD, high acuity	ST segment	1.80624E-89
CAD, high acuity	Overweight	1.08125E-84
CAD, high acuity	Blood flow	1.38304E-79
CAD, high acuity	Lesion	2.19506E-79
CAD, high acuity	Low Back Pain	9.96357E-69
CAD, high acuity	Infarction	1.04124E-63
CAD, high acuity	Obesity	2.88019E-62
CAD, high acuity	Chronic superficial gastritis	5.10849E-59
CAD, high acuity	Sclerosis	6.83839E-57
CAD, high acuity	Dyslipidemias	4.04702E-55
CAD, high acuity	Pain, Burning	1.49262E-50
CAD, high acuity	Fatigue	9.07946E-46
CAD, high acuity	Radiographic contrast agent nephropathy	9.94808E-41
CAD, high acuity	Dyspnea	1.93668E-39
CAD, high acuity	Cyanosis	4.67454E-39
CAD, high acuity	Non-Insulin-Dependent Diabetes Mellitus	1.0817E-36
CAD, high acuity	Anisocoria	1.16083E-34
CAD, high acuity	Vertebral Artery Stenosis	4.52381E-33
CAD, high acuity	Coarctation	4.71791E-33

CAD, high acuity	Diabetes Insipidus	4.86588E-33
CAD, high acuity	Occlusion of artery	2.02376E-32
CAD, high acuity	Right coronary artery occlusion	2.91189E-29
CAD, high acuity	Rest pain	4.92201E-29
CAD, high acuity	History of myocardial infarction	7.77758E-25
CAD, high acuity	Ulcer	1.37221E-24
CAD, high acuity	Hypoperfusion	4.51541E-23
CAD, high acuity	Stable angina	2.05049E-22
CAD, high acuity	Cardiac complication	2.29015E-22
CAD, high acuity	Peptic Ulcer	1.46057E-21
CAD, high acuity	Gastric ulcer	2.54541E-21
CAD, high acuity	Wheezing	4.51043E-21
CAD, high acuity	Renal tubular disorder	1.35971E-20
CAD, high acuity	Sinus bradycardia	1.17433E-18
CAD, high acuity	Chronic bronchitis	1.41489E-18
CAD, high acuity	Postoperative Complications	7.28053E-18
CAD, high acuity	Adams-Stokes Syndrome	1.18413E-17
CAD, high acuity	Kidney Calculi	9.58132E-16
CAD, high acuity	Decompression Sickness	1.7479E-15
CAD, high acuity	Pneumonia due to Klebsiella pneumoniae	9.62174E-15
CAD, high acuity	Duodenal Ulcer	3.33788E-14
CAD, high acuity	Post-op diagnosis	1.23635E-13
CAD, high acuity	Increase in blood pressure	6.13439E-12
CAD, high acuity	Atrophic Gastritis	1.72595E-11
CAD, high acuity	Spasm	3.43123E-11
CAD, high acuity	Diabetes Mellitus	1.38017E-10
CAD, high acuity	Atrophic	1.23716E-09
CAD, high acuity	Hyperesthesia	1.57721E-09
CAD, high acuity	Inguinal Hernia	1.79508E-09
CAD, high acuity	Left-Sided Heart Failure	2.2277E-09
Cardiac surgery	Blood flow	0
Cardiac surgery	Sinus rhythm	0
Cardiac surgery	Diuresis	0
Cardiac surgery	Color of urine	0
Cardiac surgery	Weakness	0
Cardiac surgery	Peristalsis	0
Cardiac surgery	Central venous pressure finding	0
Cardiac surgery	Atherosclerosis	0
Cardiac surgery	Cardiac index	0
Cardiac surgery	Angina Pectoris	0
Cardiac surgery	Stenosis	0
Cardiac surgery	Hemostatic function	0
Cardiac surgery	Left Ventricular Hypertrophy	0
Cardiac surgery	Cardiac activity	0
Cardiac surgery	Pulmonary artery pressure	0
Cardiac surgery	Chronic gastritis	0

Cardiac surgery	Hypoventilation	0
Cardiac surgery	Effusion	0
Cardiac surgery	Cerebrovascular Disorders	0
Cardiac surgery	Anastomosis	0
Cardiac surgery	Dysplasia	0
Cardiac surgery	Cardiac conduction	0
Cardiac surgery	Leukocytosis	0
Cardiac surgery	Postpericardiotomy Syndrome	0
Cardiac surgery	Decompression Sickness	0
Cardiac surgery	Coughing	0
Cardiac surgery	Cardiac Arrest	0
Cardiac surgery	Bilirubin level result	0
Cardiac surgery	Coronary Artery Disease	0
Cardiac surgery	Pleural Diseases	0
Cardiac surgery	Diaphragmatic breathing	0
Cardiac surgery	Surgical fistula	0
Cardiac surgery	Coronary Stenosis	0
Cardiac surgery	Moderate pain	0
Cardiac surgery	Wound Healing	0
Cardiac surgery	Sinus Tachycardia	0
Cardiac surgery	Acute hemorrhage	0
Cardiac surgery	Pericardial friction rub	0
Cardiac surgery	Postthrombotic Syndrome	0
Cardiac surgery	Abnormal cardiac conduction	0
Cardiac surgery	Finding of potassium level	0
Cardiac surgery	Urea level	0
Cardiac surgery	Dry tongue	0
Cardiac surgery	Finding of acid-base balance	0
Cardiac surgery	Venous Engorgement	0
Cardiac surgery	Disorder of electrolytes	0
Cardiac surgery	Microaneurysm	0
Cardiac surgery	Air Embolism	1.2469E-305
Cardiac surgery	Neurologic Deficits	4.5565E-293
Cardiac surgery	Blood coagulation pathway observation	1.2592E-284
Cardiac surgery	Kidney Failure	2.224E-279
Cardiac surgery	Cerebral Atherosclerosis	3.0056E-277
Cardiac surgery	Peripheral edema	4.2415E-276
Cardiac surgery	Hemorrhage	5.8776E-270
Cardiac surgery	Fibrous ring	2.3277E-261
Cardiac surgery	Left atrial hypertrophy	7.1723E-259
Cardiac surgery	Parotitis	5.8003E-250
Cardiac surgery	Pulmonary Fibrosis	1.0442E-242
Cardiac surgery	Myocardial Ischemia	1.4939E-235
Cardiac surgery	Lesion	1.7355E-210
Cardiac surgery	Atelectasis	1.0117E-206
Cardiac surgery	Exercise-induced angina	3.0409E-206

Cardiac surgery	Dyspnea on exertion	6.8283E-203
Cardiac surgery	Sinus bradycardia	6.8309E-203
Cardiac surgery	Aortic valve disorder	1.0596E-200
Cardiac surgery	Dyslipidemias	1.0795E-196
Cardiac surgery	Protein level	1.7979E-192
Cardiac surgery	Finding of creatinine level	2.7936E-192
Cardiac surgery	Discoid atelectasis	7.3808E-182
Cardiac surgery	Cerebrovascular accident	2.2992E-176
Cardiac surgery	Pain	1.0481E-172
Cardiac surgery	Cardiovascular Diseases	1.3792E-171
Cardiac surgery	Stupor	2.8217E-171
Cardiac surgery	Subendocardial ischemia	1.5077E-162
Cardiac surgery	Diastasis	2.1066E-156
Cardiac surgery	History of hypertension	1.6071E-152
Cardiac surgery	Respiratory Distress Syndrome	9.7713E-144
Cardiac surgery	Albumin level	1.4579E-137
Cardiac surgery	Aortic Valve Insufficiency	6.0566E-136
Cardiac surgery	Subcutaneous Emphysema	5.294E-135
Cardiac surgery	Shock	1.1865E-132
Cardiac surgery	Glucose level	8.4057E-125
Cardiac surgery	Calcification	2.7356E-116
Cardiac surgery	Finding of blood glucose level	1.544E-113
Cardiac surgery	Fibrosis	2.0093E-113
Cardiac surgery	Congestive heart failure	6.2511E-113
Cardiac surgery	Conduction disorder of the heart	5.4633E-110
Cardiac surgery	Closed fractures	3.3601E-109
Cardiac surgery	ST segment elevation	2.7788E-106
Cardiac surgery	Hypertensive heart disease	1.7417E-100
Cardiac surgery	Senile Plaques	2.823E-100
Cardiac surgery	Adhesion of diaphragm	4.31033E-94
Cardiac surgery	Diseases of mitral valve	3.19772E-92
Cardiac surgery	Respiratory Failure	1.0235E-91
Cardiac surgery	Acute myocardial infarction	4.63439E-91
Cardiac surgery	Lethargy	3.58234E-90
Cardiac surgery	Tissue Adhesions	8.1888E-87
Cardiac surgery	Infertility	1.16412E-85
Cardiac surgery	Unstable angina	1.1877E-85
Cardiac surgery	Prostatic Hyperplasia	2.76955E-85
Cerebrovascular disease	Dysarthria	0
Cerebrovascular disease	Gagging	0
Cerebrovascular disease	Encephalopathies	0
Cerebrovascular disease	Corneal Reflexes	0
Cerebrovascular disease	Nystagmus	0
Cerebrovascular disease	Pupil reaction to light	0
Cerebrovascular disease	Dysphonia	0
Cerebrovascular disease	Deglutition Disorders	0

Cerebrovascular disease	Dizziness	0
Cerebrovascular disease	Cerebral Atherosclerosis	0
Cerebrovascular disease	Muscle Tension	0
Cerebrovascular disease	Ataxia	0
Cerebrovascular disease	Headache	0
Cerebrovascular disease	Diadochokinesia	0
Cerebrovascular disease	Ischemic stroke	0
Cerebrovascular disease	Hemiparesis	0
Cerebrovascular disease	Numbness	0
Cerebrovascular disease	Cerebral Infarction	0
Cerebrovascular disease	Hydrocephalus	0
Cerebrovascular disease	Neurologic Deficits	0
Cerebrovascular disease	Closed head injuries	0
Cerebrovascular disease	Hypesthesia	0
Cerebrovascular disease	Amnesia	0
Cerebrovascular disease	Cerebellar decompression injury	0
Cerebrovascular disease	Neck Pain	0
Cerebrovascular disease	Carotid Stenosis	0
Cerebrovascular disease	Back Pain	0
Cerebrovascular disease	Gliosis	0
Cerebrovascular disease	Hemianopsia	0
Cerebrovascular disease	Low grade fever	0
Cerebrovascular disease	Broca Aphasia	0
Cerebrovascular disease	Spastic hemiplegia	0
Cerebrovascular disease	Pseudobulbar Palsy	0
Cerebrovascular disease	Incontinence	0
Cerebrovascular disease	Cerebral Embolism	0
Cerebrovascular disease	Cognition Disorders	1.142E-303
Cerebrovascular disease	Hypertensive Encephalopathy	9.8389E-294
Cerebrovascular disease	Disorder of circulatory system	5.6912E-272
Cerebrovascular disease	Left hemiparesis	1.0054E-266
Cerebrovascular disease	Vertigo	9.5529E-266
Cerebrovascular disease	Neurologic Symptoms	2.2169E-255
Cerebrovascular disease	Scheuermann's Disease	8.1104E-251
Cerebrovascular disease	Transient Ischemic Attack	5.0543E-246
Cerebrovascular disease	Paralysed	4.3825E-236
Cerebrovascular disease	Lordosis	6.0855E-231
Cerebrovascular disease	Spondylarthropathies	1.9813E-220
Cerebrovascular disease	Squeezing pain	1.9619E-200
Cerebrovascular disease	Cerebrovascular accident	1.7436E-190
Cerebrovascular disease	Arm Pain	1.1093E-189
Cerebrovascular disease	Mixed aphasia	2.3325E-186
Cerebrovascular disease	Secondary hypertension	3.3784E-185
Cerebrovascular disease	Pulmonary resonance	1.765E-178
Cerebrovascular disease	Atherosclerosis	4.6298E-170
Cerebrovascular disease	Apraxia	5.3294E-157

Cerebrovascular disease	Spondylosis	5.3323E-156
Cerebrovascular disease	Sensory polyneuropathy	8.6057E-153
Cerebrovascular disease	Pain in lumbar spine	1.1435E-150
Cerebrovascular disease	Traumatic Brain Injury	9.9568E-149
Cerebrovascular disease	Stupor	9.0373E-147
Cerebrovascular disease	Diplopia	1.8362E-130
Cerebrovascular disease	Sleep disturbances	4.1443E-128
Cerebrovascular disease	Spinal Stenosis	5.227E-119
Cerebrovascular disease	Changes in retinal vascular appearance	2.0719E-117
Cerebrovascular disease	Hyperventilation	1.9986E-113
Cerebrovascular disease	Stable blood pressure	1.326E-109
Cerebrovascular disease	Stenosis	5.11204E-97
Cerebrovascular disease	Cataract	1.08287E-95
Cerebrovascular disease	Vascular resistance	2.47006E-93
Cerebrovascular disease	Muscular Atrophy	2.62049E-93
Cerebrovascular disease	Low Back Pain	2.27329E-91
Cerebrovascular disease	Mild cognitive disorder	4.2734E-89
Cerebrovascular disease	Neuropathy	1.72229E-87
Cerebrovascular disease	Pain in cervical spine	3.94725E-85
Cerebrovascular disease	Disk, Herniated	5.48704E-85
Cerebrovascular disease	Radiculopathy	3.23555E-84
Cerebrovascular disease	Cognitive functions	2.23383E-79
Cerebrovascular disease	Ischemia	7.79046E-77
Cerebrovascular disease	Right hemiparesis	1.64589E-72
Cerebrovascular disease	T wave feature	2.38639E-67
Cerebrovascular disease	Tinnitus	9.81208E-67
Cerebrovascular disease	Presbyopia	1.29265E-65
Cerebrovascular disease	Cataract secondary to ocular disorder	6.23157E-65
Cerebrovascular disease	Generalized convulsive epilepsy	6.93005E-65
Cerebrovascular disease	Mydriasis	1.19526E-64
Cerebrovascular disease	Tremor	2.63701E-63
Cerebrovascular disease	Osteochondrosis	3.03162E-63
Cerebrovascular disease	Vascular Diseases	4.7383E-61
Cerebrovascular disease	Brain hemorrhage	7.81263E-59
Cerebrovascular disease	Plaque (lesion)	1.00142E-56
Cerebrovascular disease	Kyphosis deformity of spine	3.1536E-54
Cerebrovascular disease	Deglutition	2.02962E-53
Cerebrovascular disease	Blurred vision	2.88018E-53
Cerebrovascular disease	Senile Plaques	2.83425E-52
Cerebrovascular disease	Unsteady gait	9.10794E-52
Cerebrovascular disease	Polyneuropathy	1.41383E-50
Cerebrovascular disease	Spinal Diseases	7.96874E-47
Cerebrovascular disease	Adhesion of pleura	2.59496E-46
Cerebrovascular disease	Generalized atherosclerosis	2.28292E-45
Cerebrovascular disease	Fetal Movement	7.27013E-45
Cerebrovascular disease	Vision	1.82709E-44

Congenital heart disease	Atrial Septal Defects	0
Congenital heart disease	Congenital heart disease	0
Congenital heart disease	Congenital Abnormality	0
Congenital heart disease	Congenital Heart Defects	0
Congenital heart disease	Air Embolism	0
Congenital heart disease	Respiration Disorders	0
Congenital heart disease	Fluid overload	0
Congenital heart disease	Birth	9.1518E-307
Congenital heart disease	Right Ventricular Hypertrophy	2.7883E-270
Congenital heart disease	Pregnancy	5.2237E-222
Congenital heart disease	Cardiac activity	1.1301E-201
Congenital heart disease	Under local anesthesia	2.8329E-160
Congenital heart disease	Systolic Murmurs	3.1978E-157
Congenital heart disease	Childbirth	2.2027E-143
Congenital heart disease	Hereditary Diseases	8.3052E-139
Congenital heart disease	Endocardial Cushion Defects	1.0712E-118
Congenital heart disease	Splitting sensation	1.9669E-113
Congenital heart disease	Toxemia	1.0362E-106
Congenital heart disease	Surgical wound	2.2841E-106
Congenital heart disease	Viral respiratory infection	1.94743E-87
Congenital heart disease	Ebstein Anomaly	1.53156E-86
Congenital heart disease	Acute respiratory disease	3.53197E-86
Congenital heart disease	Laboratory test finding	1.6267E-81
Congenital heart disease	Heart Septal Defects	1.8279E-74
Congenital heart disease	Failure to gain weight	7.87284E-71
Congenital heart disease	Pulmonary Hypertension	2.56166E-67
Congenital heart disease	Psychomotor development	1.28757E-65
Congenital heart disease	Underweight	2.68532E-64
Congenital heart disease	Heart murmur	9.65213E-62
Congenital heart disease	Hydration status	4.19067E-51
Congenital heart disease	Ventricular Septal Defects	5.15357E-48
Congenital heart disease	Perforation	4.44988E-43
Congenital heart disease	Atrioventricular Septal Defect	8.87857E-41
Congenital heart disease	Hypothermia	1.1924E-37
Congenital heart disease	Hemostatic function	2.15206E-36
Congenital heart disease	Vaginitis	2.57615E-35
Congenital heart disease	Gas flow	3.97356E-35
Congenital heart disease	Invasive arterial pressure	7.97078E-34
Congenital heart disease	Sedated state	3.13139E-33
Congenital heart disease	Pre-Eclampsia	1.91059E-32
Congenital heart disease	Weight Gain	2.87294E-32
Congenital heart disease	Liver edge	3.3782E-32
Congenital heart disease	Cardiomegaly	1.32909E-31
Congenital heart disease	Color of urine	4.80771E-31
Congenital heart disease	Pulmonary arterial hypertension	5.32303E-31
Congenital heart disease	Electrocardiogram axis finding	1.25235E-29

Congenital heart disease	Dermatologic disorders	8.65169E-29
Congenital heart disease	Pulmonary Valve Stenosis	4.31999E-27
Congenital heart disease	Nasopharyngitis	3.3879E-25
Congenital heart disease	Atopic dermatitis	7.59225E-24
Congenital heart disease	Pulmonary Stenosis	4.06892E-23
Congenital heart disease	Primigravida	4.73376E-22
Congenital heart disease	Down Syndrome	8.45252E-22
Congenital heart disease	Fetal Growth Retardation	1.99702E-18
Congenital heart disease	Esophageal Diseases	3.9703E-18
Congenital heart disease	Diuresis	5.89649E-16
Congenital heart disease	Ear Inflammation	2.13539E-15
Congenital heart disease	Ptosis	4.27151E-15
Congenital heart disease	Rickets	7.07628E-15
Congenital heart disease	Urinary tract infection	4.91614E-14
Congenital heart disease	Cold sweat	1.47684E-13
Congenital heart disease	Psychomotor development	3.4843E-13
Congenital heart disease	Pulpitis	3.39419E-12
Congenital heart disease	Evoked Potentials	8.33051E-12
Congenital heart disease	Pharyngitis	1.40211E-11
Congenital heart disease	Common atrioventricular canal	2.01357E-11
Congenital heart disease	Productive cough	2.34388E-11
Congenital heart disease	Electrical activity of brain	5.334E-11
Congenital heart disease	Scoliosis	3.47085E-10
Congenital heart disease	Valgus deformity	4.44827E-10
Congenital heart disease	Transposition of Great Vessels	8.87948E-09
Congenital heart disease	Tired	9.10421E-09
Diabetic complications	Non-Insulin-Dependent Diabetes Mellitus	0
Diabetic complications	Diabetes Mellitus	0
Diabetic complications	Diabetic Polyneuropathies	0
Diabetic complications	Diabetic Nephropathy	0
Diabetic complications	Insulin-Dependent Diabetes Mellitus	0
Diabetic complications	Retinal Diseases	0
Diabetic complications	Kidney Diseases	0
Diabetic complications	Cataract	0
Diabetic complications	Hyperglycemia	0
Diabetic complications	Vascular Diseases	0
Diabetic complications	Complications of Diabetes Mellitus	0
Diabetic complications	Endocarditis	0
Diabetic complications	Chronic Kidney Diseases	0
Diabetic complications	Glucose level	0
Diabetic complications	Diabetic Retinopathy	0
Diabetic complications	Polyneuropathy	0
Diabetic complications	Nonproliferative diabetic retinopathy	0
Diabetic complications	Proteinuria	0
Diabetic complications	Hypoglycemia	0
Diabetic complications	Xerostomia	0

Diabetic complications	Diabetic Foot	0
Diabetic complications	Blood coagulation	0
Diabetic complications	History of diabetes mellitus type 2	0
Diabetic complications	Paresthesia	0
Diabetic complications	Poor glycemic control	0
Diabetic complications	Proliferative retinopathy	0
Diabetic complications	Diabetic maculopathy	0
Diabetic complications	Deglutition	8.7377E-304
Diabetic complications	Obesity	9.1848E-298
Diabetic complications	Mydriasis	3.1779E-296
Diabetic complications	Exophthalmos	9.6142E-232
Diabetic complications	Glycosuria	1.888E-201
Diabetic complications	Finding of blood glucose level	5.1558E-199
Diabetic complications	Hydrocephalus, Normal Pressure	3.8644E-187
Diabetic complications	Pain in limb	4.1889E-163
Diabetic complications	Decompensation	7.8977E-151
Diabetic complications	Muscle Cramp	2.3017E-137
Diabetic complications	Hyperopia	2.5033E-136
Diabetic complications	Diabetic Neuropathies	1.9291E-129
Diabetic complications	Peripheral perfusion	6.8301E-119
Diabetic complications	Chronic kidney disease stage 3	7.489E-112
Diabetic complications	Presbyopia	1.2191E-107
Diabetic complications	Myopic astigmatism	5.7627E-105
Diabetic complications	Essential Hypertension	1.2882E-103
Diabetic complications	Chronic kidney disease stage 1	2.24121E-98
Diabetic complications	Stomach Diseases	2.6874E-95
Diabetic complications	Pain in lower limb	5.1988E-89
Diabetic complications	Blurred vision	5.86229E-89
Diabetic complications	Chronic kidney disease stage 4	5.25756E-84
Diabetic complications	Numbness	1.23304E-75
Diabetic complications	Ulcer	3.77901E-72
Diabetic complications	Chronic kidney disease stage 2	8.3234E-72
Diabetic complications	Mixed astigmatism	2.00904E-71
Diabetic complications	Changes in retinal vascular appearance	1.28791E-69
Diabetic complications	Foot pain	1.44744E-67
Diabetic complications	Intraocular pressure disorder	1.25952E-65
Diabetic complications	Chronic Kidney Failure	4.08766E-60
Diabetic complications	Nontoxic goiter	6.56337E-55
Diabetic complications	Macular degeneration	9.82923E-48
Diabetic complications	Glaucoma	3.46536E-47
Diabetic complications	Dry Eye Syndromes	7.5335E-47
Diabetic complications	Arterial insufficiency	4.30409E-46
Diabetic complications	Phlegmon	2.4266E-44
Diabetic complications	Autoimmune thyroiditis	7.28076E-44
Diabetic complications	Myopia	1.70396E-43
Diabetic complications	Morbid obesity	4.81654E-41

Diabetic complications	Astigmatism	1.59361E-35
Diabetic complications	Exocrine pancreatic insufficiency	1.89329E-31
Diabetic complications	Thyroid Nodule	2.64985E-30
Diabetic complications	Candidemia	1.57484E-29
Diabetic complications	Intra-atrial conduction	2.42525E-29
Diabetic complications	Menopause	2.30873E-28
Diabetic complications	Epithelial metaplasia	1.71014E-27
Diabetic complications	Gynecological history	1.623E-26
Diabetic complications	Blood coagulation pathway observation	5.78171E-25
Diabetic complications	Wound Healing	1.75808E-24
Diabetic complications	Pathologic Neovascularization	5.54516E-23
Diabetic complications	Neuropathy	1.40658E-22
Diabetic complications	Chronic kidney disease stage 5	4.14215E-21
Diabetic complications	Intermittent Claudication	1.26674E-19
Diabetic complications	Urine microscopy leukocytes present finding	1.74728E-19
Diabetic complications	Tracheobronchitis	1.72784E-17
Diabetic complications	Under general anesthesia	8.99631E-17
Diabetic complications	Dermatologic disorders	1.86314E-16
Diabetic complications	Pain in thoracic spine	2.07946E-16
Diabetic complications	Osteopenia	2.32206E-16
Diabetic complications	Subclinical hypothyroidism	2.71832E-16
Diabetic complications	Pneumonia due to Klebsiella pneumoniae	1.00376E-15
Diabetic complications	Osteomyelitis	1.70205E-15
Diabetic complications	Dyslipidemias	2.78931E-15
Diabetic complications	Position of uterus	7.31694E-14
Diabetic complications	Familial hypercholesterolemia	1.7232E-12
Diabetic complications	Osteoporosis	3.39201E-12
Diabetic complications	Finding of creatinine level	5.52365E-12
Diabetic complications	Pneumonia, Bacterial	9.51872E-12
Diabetic complications	Weight decreased	1.25973E-11
Diabetic complications	Internal Carotid Artery Stenosis	4.20326E-11
Diabetic complications	Hyperuricemia	4.44499E-11
Diabetic complications	Anemia of chronic disease	4.97052E-11
Diabetic complications	Severe pain	1.69592E-10
Dilated cardiomyopathy	Dilated cardiomyopathy	0
Dilated cardiomyopathy	Chronic heart failure	0
Dilated cardiomyopathy	Cardiomyopathies	0
Dilated cardiomyopathy	Mitral Valve Insufficiency	0
Dilated cardiomyopathy	Ventricular Tachycardia	0
Dilated cardiomyopathy	Cardiomegaly	0
Dilated cardiomyopathy	Myocarditis	0
Dilated cardiomyopathy	Paroxysmal ventricular tachycardia	0
Dilated cardiomyopathy	Esophageal Diseases	3.3941E-245
Dilated cardiomyopathy	Hernia	2.6213E-185
Dilated cardiomyopathy	Dyspnea	2.7228E-182
Dilated cardiomyopathy	Interventricular dyssynchrony	3.5212E-168

Dilated cardiomyopathy	Hyperuricemia	9.3399E-130
Dilated cardiomyopathy	Pulmonary Thromboembolisms	2.4227E-127
Dilated cardiomyopathy	Tricuspid Valve Insufficiency	8.2275E-124
Dilated cardiomyopathy	Hypokinesia	6.5911E-119
Dilated cardiomyopathy	Premature Cardiac Complex	8.9348E-119
Dilated cardiomyopathy	Splenomegaly	6.9562E-107
Dilated cardiomyopathy	Atrial Fibrillation	8.9875E-104
Dilated cardiomyopathy	Pulmonary Embolism	2.08737E-95
Dilated cardiomyopathy	Premature ventricular contractions	2.15389E-93
Dilated cardiomyopathy	Fever	3.2429E-89
Dilated cardiomyopathy	Obesity	2.6121E-86
Dilated cardiomyopathy	Cardiac dilatation	4.71889E-86
Dilated cardiomyopathy	Secondary dilated cardiomyopathy	1.16449E-85
Dilated cardiomyopathy	Chronic Obstructive Airway Disease	6.97791E-83
Dilated cardiomyopathy	Chronic liver disease	1.98499E-81
Dilated cardiomyopathy	Asphyxia	1.89877E-71
Dilated cardiomyopathy	Premature Cardiac Complex	1.13475E-70
Dilated cardiomyopathy	Pathological Dilatation	6.2521E-60
Dilated cardiomyopathy	Ascites	8.46998E-59
Dilated cardiomyopathy	Cardiac asthma	1.0016E-55
Dilated cardiomyopathy	Thromboembolism	3.39126E-53
Dilated cardiomyopathy	Pulmonary Hypertension	2.95235E-50
Dilated cardiomyopathy	Hepatomegaly	9.67125E-50
Dilated cardiomyopathy	Thrombophilia	1.15959E-47
Dilated cardiomyopathy	Choking	5.71175E-45
Dilated cardiomyopathy	Viral myocarditis	2.12394E-43
Dilated cardiomyopathy	Recurrent pulmonary embolism	4.62197E-42
Dilated cardiomyopathy	Cardiac finding	1.81635E-40
Dilated cardiomyopathy	Ventricular Fibrillation	2.35185E-39
Dilated cardiomyopathy	Hiatal Hernia	6.26091E-37
Dilated cardiomyopathy	Swelling	3.65943E-36
Dilated cardiomyopathy	Post-op diagnosis	5.28203E-32
Dilated cardiomyopathy	Sudden Cardiac Death	3.15416E-31
Dilated cardiomyopathy	Gout	1.77189E-29
Dilated cardiomyopathy	Hyperhomocysteinemia	1.26389E-27
Dilated cardiomyopathy	Cardiovascular finding	1.59934E-26
Dilated cardiomyopathy	Permanent atrial fibrillation	2.79847E-26
Dilated cardiomyopathy	Varicosity	1.23486E-25
Dilated cardiomyopathy	Chronic myocarditis	1.8109E-25
Dilated cardiomyopathy	Decompensation	8.98233E-24
Dilated cardiomyopathy	Hemoptysis	9.19611E-24
Dilated cardiomyopathy	Left Bundle-Branch Block	2.75278E-23
Dilated cardiomyopathy	Viral respiratory infection	5.05334E-23
Dilated cardiomyopathy	Asthma attack	2.20603E-21
Dilated cardiomyopathy	Exhaustion	7.00689E-21
Dilated cardiomyopathy	Toxic hepatisis	3.12671E-20

Dilated cardiomyopathy	Overweight	2.06402E-19
Dilated cardiomyopathy	Esophageal Hernia	1.10388E-18
Dilated cardiomyopathy	Eccentric hypertrophy	9.1574E-18
Dilated cardiomyopathy	Nephrosclerosis	1.43055E-17
Dilated cardiomyopathy	Hydrothorax	1.99495E-17
Dilated cardiomyopathy	Edema	2.14922E-17
Dilated cardiomyopathy	Weight decreased	2.60953E-17
Dilated cardiomyopathy	Hepatosplenomegaly	8.43113E-16
Dilated cardiomyopathy	Cardiomyopathy associated with another disorder	1.47731E-14
Dilated cardiomyopathy	Ventricular parasystole	1.86762E-14
Dilated cardiomyopathy	Liver Cirrhosis	1.96907E-14
Dilated cardiomyopathy	Persistent atrial fibrillation	7.99356E-14
Dilated cardiomyopathy	Systolic dysfunction	1.24345E-13
Dilated cardiomyopathy	Erythrocytosis	5.16138E-13
Dilated cardiomyopathy	Subclinical hypothyroidism	9.10602E-13
Dilated cardiomyopathy	Thrombophilia, hereditary	1.52204E-12
Dilated cardiomyopathy	Heart murmur	2.13366E-12
Dilated cardiomyopathy	Acute myocarditis	3.24629E-12
Dilated cardiomyopathy	Bronchitis	5.79536E-12
Dilated cardiomyopathy	Chronic sinusitis	2.02771E-11
Dilated cardiomyopathy	Hepatitis B	2.05487E-11
Dilated cardiomyopathy	Posttransfusion purpura	2.17757E-11
Dilated cardiomyopathy	Hyperuricemia without signs of inflammatory arth	2.48393E-11
Dilated cardiomyopathy	Atrial Flutter	2.63962E-11
Dilated cardiomyopathy	Chronic erosive gastritis	3.3844E-11
Dilated cardiomyopathy	Gilbert Disease	9.30536E-11
Dilated cardiomyopathy	Sustained ventricular tachycardia	1.32719E-10
Dilated cardiomyopathy	Chronic kidney disease stage 2	2.75142E-10
Dilated cardiomyopathy	Liver fibrosis	6.60005E-10
Dilated cardiomyopathy	Anasarca	7.13002E-10
Dilated cardiomyopathy	Dry cough	8.02595E-10
Dilated cardiomyopathy	Fatty degeneration	6.00163E-09
Dilated cardiomyopathy	Heart failure	7.08573E-09
Dilated cardiomyopathy	Liver cyst	7.45443E-09
Dilated cardiomyopathy	Ablation frequency	9.65318E-09
Dilated cardiomyopathy	Noninflammatory pericardial Effusion	9.71969E-09
Echocardiography	Mitral Valve Insufficiency	0
Echocardiography	Tricuspid Valve Insufficiency	0
Echocardiography	Cerebrovascular accident	0
Echocardiography	Pulmonary Valve Insufficiency	0
Echocardiography	Diastolic dysfunction	0
Echocardiography	Aortic Valve Insufficiency	0
Echocardiography	Hypokinesia	0
Echocardiography	Pulmonary Hypertension	0
Echocardiography	Calcification	0
Echocardiography	Akinesia	0

Echocardiography	Eccentric hypertrophy	0
Echocardiography	Muscle Rigidity	0
Echocardiography	Concentric hypertrophy	0
Echocardiography	Fibrosis	0
Echocardiography	Idiopathic pulmonary arterial hypertension	0
Echocardiography	Dysfunction of papillary muscle	0
Echocardiography	Left Ventricular Hypertrophy	2.6562E-210
Echocardiography	Blood flow	9.424E-151
Echocardiography	Pathological Dilatation	3.8044E-119
Echocardiography	Cardiac dyskinesia	2.0774E-104
Echocardiography	Aortic Valve Stenosis	9.09412E-94
Echocardiography	Mitral Valve Stenosis	2.94151E-76
Echocardiography	Ptosis	6.29098E-76
Echocardiography	Decompression Sickness	4.16076E-55
Echocardiography	Left ventricular diastolic dysfunction	2.67458E-27
Echocardiography	Arrhythmogenic Right Ventricular Dysplasia	1.24658E-25
Echocardiography	Atrial dilatation	1.06633E-24
Echocardiography	Atrial Fibrillation	4.98705E-21
Echocardiography	Hypertrophy	2.17705E-17
Echocardiography	Fibrous ring	1.6462E-16
Echocardiography	Aortic valve area	4.70416E-16
Echocardiography	Cardiomegaly	5.32957E-15
Echocardiography	Systolic dysfunction	4.84056E-13
Echocardiography	Fluid overload	5.66838E-12
Echocardiography	Intraventricular conduction defect	5.59575E-09
Electrocardiography	Cardiac Arrhythmia	0
Electrocardiography	Sinus rhythm	0
Electrocardiography	Premature ventricular contractions	0
Electrocardiography	Atrial Premature Complexes	0
Electrocardiography	Premature Cardiac Complex	0
Electrocardiography	Wakefulness	0
Electrocardiography	Ventricular arrhythmia	0
Electrocardiography	Supraventricular arrhythmia	0
Electrocardiography	Bradycardia	0
Electrocardiography	Tachycardia	0
Electrocardiography	Respiratory Insufficiency	0
Electrocardiography	Atrial tachycardia	0
Electrocardiography	Sinus Arrhythmia	0
Electrocardiography	ST segment	0
Electrocardiography	Parasystole	0
Electrocardiography	Atrial rhythm	1.252E-297
Electrocardiography	Muscle Rigidity	4.3145E-230
Electrocardiography	Supraventricular tachycardia	1.2107E-202
Electrocardiography	Premature Birth	5.2091E-190
Electrocardiography	Decreased systolic arterial pressure	4.6557E-142
Electrocardiography	Atrioventricular Block	1.4902E-140

Electrocardiography	Acclimatization	3.0704E-139
Electrocardiography	Tachypnea	5.175E-130
Electrocardiography	Ventricular Tachycardia	4.2332E-125
Electrocardiography	Rhythm from artificial pacing	2.09356E-93
Electrocardiography	Idioventricular rhythm	2.71046E-65
Electrocardiography	Polymorphic ventricular tachycardia	3.84074E-63
Electrocardiography	Peripheral edema	1.49392E-56
Electrocardiography	Systolic hypertension	4.0976E-56
Electrocardiography	Ventricular parasystole	2.13201E-54
Electrocardiography	Polycythemia	1.37929E-46
Electrocardiography	Cardiac conduction	9.16219E-39
Electrocardiography	Monomorphic ventricular tachycardia	9.18895E-39
Electrocardiography	Palpitations	3.88219E-34
Electrocardiography	Sleep Apnea Syndromes	1.0277E-30
Electrocardiography	Cardiac rhythm type	2.49367E-27
Electrocardiography	Intraventricular conduction defect	6.4829E-24
Electrocardiography	Bradyarrhythmia	2.03704E-23
Electrocardiography	Fatigue	2.14788E-22
Electrocardiography	Evoked Potentials	4.75325E-20
Electrocardiography	Sudden death	2.50791E-18
Electrocardiography	Lymphocytic myocarditis	2.55361E-18
Electrocardiography	Premature Cardiac Complex	5.32796E-18
Electrocardiography	Atrioventricular junctional rhythm	4.10001E-17
Electrocardiography	Systemic arterial pressure	5.50152E-16
Electrocardiography	Heart beat	7.30711E-16
Electrocardiography	Dissociation	2.0525E-15
Electrocardiography	Neutrophilia	9.29274E-15
Electrocardiography	Auriculo-Ventricular Dissociation	1.00982E-14
Electrocardiography	Endometriosis	2.52649E-14
Electrocardiography	Ablation frequency	9.67759E-13
Electrocardiography	Early repolarization	8.78967E-12
Electrocardiography	Sinus bradycardia	1.05086E-11
Electrocardiography	Viral myocarditis	9.02397E-11
Electrocardiography	Cardiac pain	2.39279E-10
Electrocardiography	Conduction disorder of the heart	1.52938E-09
Electrocardiography	Paroxysmal atrial tachycardia	2.36859E-09
Electrocardiography	Hypotension	4.76413E-09
Electrocardiography	Sick Sinus Syndrome	4.92715E-09
Electrocardiography	Paroxysmal tachycardia	8.25581E-09
Electrocardiography w/ apnea	Slow shallow breathing	0
Electrocardiography w/ apnea	Apnea	0
Electrocardiography w/ apnea	Cardiac Arrhythmia	0
Electrocardiography w/ apnea	Premature ventricular contractions	0
Electrocardiography w/ apnea	Sinus rhythm	0
Electrocardiography w/ apnea	Atrial Premature Complexes	0
Electrocardiography w/ apnea	Premature Cardiac Complex	0

Electrocardiography w/ apnea	Supraventricular arrhythmia	0
Electrocardiography w/ apnea	Ventricular arrhythmia	0
Electrocardiography w/ apnea	Bradycardia	0
Electrocardiography w/ apnea	Atrial tachycardia	0
Electrocardiography w/ apnea	Sinus Arrhythmia	0
Electrocardiography w/ apnea	Respiratory Insufficiency	1.2428E-277
Electrocardiography w/ apnea	Tachycardia	4.0907E-263
Electrocardiography w/ apnea	Decreased systolic arterial pressure	1.7853E-222
Electrocardiography w/ apnea	Muscle Rigidity	3.7689E-145
Electrocardiography w/ apnea	Systemic arterial pressure	2.9717E-105
Electrocardiography w/ apnea	Tachypnea	7.29207E-77
Electrocardiography w/ apnea	Wakefulness	8.37624E-75
Electrocardiography w/ apnea	ST segment	1.29036E-59
Electrocardiography w/ apnea	Supraventricular tachycardia	2.54379E-41
Electrocardiography w/ apnea	Fatigue	5.62571E-34
Electrocardiography w/ apnea	Dyspnea	1.07206E-24
Electrocardiography w/ apnea	Atrioventricular Block	1.7467E-22
Electrocardiography w/ apnea	Pulmonary Valve Insufficiency	1.53236E-20
Electrocardiography w/ apnea	Atrial Fibrillation	1.74913E-16
Electrocardiography w/ apnea	Atrial rhythm	3.29597E-15
Electrocardiography w/ apnea	Paroxysmal atrial tachycardia	1.55644E-13
Electrocardiography w/ apnea	Diastolic dysfunction	2.34698E-12
Electrocardiography w/ apnea	Intraventricular conduction defect	3.7789E-12
Electrocardiography w/ apnea	Parasystole	6.86609E-12
Electrocardiography w/ apnea	Smoking History	2.36572E-11
Electrocardiography w/ apnea	Neutrophilia	4.58282E-10
General vascular disease	Stenosis	0
General vascular disease	Blood flow	0
General vascular disease	Cerebrovascular accident	0
General vascular disease	Senile Plaques	0
General vascular disease	Plaque (lesion)	0
General vascular disease	Decompression Sickness	0
General vascular disease	Atrophic	0
General vascular disease	Lung consolidation	0
General vascular disease	Peak systolic	0
General vascular disease	Systemic Scleroderma	1.9241E-305
General vascular disease	Atherosclerosis	2.3082E-275
General vascular disease	Atrophic Vaginitis	1.0757E-218
General vascular disease	Stomach Diseases	9.1776E-204
General vascular disease	Carotid Stenosis	1.627E-189
General vascular disease	Superficial ulcer	5.5916E-176
General vascular disease	Calcification	1.4495E-145
General vascular disease	Pathologic calcification, calcified structure	3.9231E-140
General vascular disease	Chronic myocardial ischemia	3.0693E-131
General vascular disease	Atherosclerosis of aorta	1.248E-119
General vascular disease	Secondary hypertension	1.6825E-116

General vascular disease	Hyperemia	4.7979E-103
General vascular disease	Erosive gastritis	2.57796E-92
General vascular disease	Intermittent Claudication	1.70605E-91
General vascular disease	Vascular resistance	2.09355E-90
General vascular disease	Generalized atherosclerosis	6.9306E-88
General vascular disease	Vasodilation	5.81602E-59
General vascular disease	Hypoplasia	3.76699E-56
General vascular disease	Arterial insufficiency	1.90506E-53
General vascular disease	Hyperplasia	1.92934E-41
General vascular disease	Lower limb ischemia	2.48359E-39
General vascular disease	Apley compression test response	5.40912E-39
General vascular disease	Myalgia	3.2748E-36
General vascular disease	Coarctation	3.29925E-30
General vascular disease	Arrhythmogenic Right Ventricular Dysplasia	1.03759E-28
General vascular disease	Chronic superficial ulcer	9.02957E-26
General vascular disease	Lesion	4.01891E-24
General vascular disease	Pain in lower limb	5.22012E-24
General vascular disease	Secondary hyperparathyroidism	1.89549E-23
General vascular disease	Pain in limb	2.26309E-23
General vascular disease	Acute superficial ulcer	4.65067E-23
General vascular disease	Vaginitis	1.27273E-19
General vascular disease	Atrophic Gastritis	3.53273E-19
General vascular disease	Internal Carotid Artery Stenosis	7.71687E-18
General vascular disease	Prostatic Hyperplasia	2.21479E-16
General vascular disease	Focal atrophy	6.57942E-15
General vascular disease	Pupil reaction to light	1.35622E-13
General vascular disease	Embolism	8.40499E-13
General vascular disease	Vertebral Artery Stenosis	8.76328E-13
General vascular disease	Hypercapnia	1.01767E-12
General vascular disease	Chronic superficial gastritis	4.84295E-12
General vascular disease	Amputation Stumps	7.67149E-12
General vascular disease	Gagging	1.2522E-11
General vascular disease	Ulceration	2.40667E-11
General vascular disease	Urolithiasis	3.68876E-11
General vascular disease	Smoking History	7.36215E-11
General vascular disease	Ischemia	3.04547E-10
General vascular disease	Gastric reflux finding	4.84554E-10
General vascular disease	Dysarthria	2.08812E-09
General vascular disease	Angina Pectoris	4.61144E-09
General vascular disease	Position of kidney	8.4917E-09
Hypertrophic cardiomyopathy	Hypertrophic Cardiomyopathy	0
Hypertrophic cardiomyopathy	Hypertrophy	0
Hypertrophic cardiomyopathy	Hypertrophic cardiomyopathy without obstructio	0
Hypertrophic cardiomyopathy	Dynamic obstruction	0
Hypertrophic cardiomyopathy	Asymmetric hypertrophy	0
Hypertrophic cardiomyopathy	Primary Cardiomyopathies	0

Hypertrophic cardiomyopathy	Left Ventricular Hypertrophy	2.1079E-218
Hypertrophic cardiomyopathy	QRS complex feature	5.8721E-144
Hypertrophic cardiomyopathy	Subaortic stenosis	5.0297E-138
Hypertrophic cardiomyopathy	Heart murmur	3.7431E-123
Hypertrophic cardiomyopathy	Syncope	6.5091E-118
Hypertrophic cardiomyopathy	Sudden Cardiac Death	2.06752E-89
Hypertrophic cardiomyopathy	Systolic Murmurs	2.11919E-67
Hypertrophic cardiomyopathy	Diastolic dysfunction	1.30015E-66
Hypertrophic cardiomyopathy	Blood flow	2.42276E-65
Hypertrophic cardiomyopathy	Presyncope	3.32684E-59
Hypertrophic cardiomyopathy	Mitral Valve Insufficiency	1.00442E-53
Hypertrophic cardiomyopathy	Dizziness	4.00827E-35
Hypertrophic cardiomyopathy	Ventricular Tachycardia	5.22958E-35
Hypertrophic cardiomyopathy	Pulmonary Valve Insufficiency	1.58672E-29
Hypertrophic cardiomyopathy	Concentric hypertrophy	1.00011E-27
Hypertrophic cardiomyopathy	Hyperlipidemia	3.4278E-23
Hypertrophic cardiomyopathy	Weight decreased	4.02895E-23
Hypertrophic cardiomyopathy	Fever	6.95367E-23
Hypertrophic cardiomyopathy	Capillary malformation	2.17654E-21
Hypertrophic cardiomyopathy	Chronic liver disease	1.06145E-20
Hypertrophic cardiomyopathy	Unspecified Abortion	4.54916E-20
Hypertrophic cardiomyopathy	Overweight	4.75362E-19
Hypertrophic cardiomyopathy	Pregnancy	6.35497E-17
Hypertrophic cardiomyopathy	Dyspnea	9.71192E-15
Hypertrophic cardiomyopathy	Gynecological history	1.18345E-13
Hypertrophic cardiomyopathy	Muscle Rigidity	2.96733E-13
Hypertrophic cardiomyopathy	Hypercholesterolemia	3.63803E-12
Hypertrophic cardiomyopathy	Obesity	8.9124E-12
Hypertrophic cardiomyopathy	Endometriosis	1.11134E-11
Hypertrophic cardiomyopathy	Chronic Hepatitis B	1.42024E-11
Hypertrophic cardiomyopathy	Fibroid Tumor	3.87111E-11
Hypertrophic cardiomyopathy	Heart beat	8.11435E-11
Hypertrophic cardiomyopathy	Palpitations	2.11968E-10
Hypertrophic cardiomyopathy	Menopause	3.72474E-10
Hypertrophic cardiomyopathy	Left atrial hypertrophy	8.2319E-10
Hypertrophic cardiomyopathy	Cardiomyopathies	3.33611E-09
Hypertrophic cardiomyopathy	Paroxysmal supraventricular tachycardia	3.46344E-09
Hypertrophic cardiomyopathy	Hypertensive disease	6.17325E-09
Lipid disorders	Lipid Metabolism Disorders	0
Lipid disorders	Overweight	0
Lipid disorders	erythrocyte sedimentation rate result	0
Lipid disorders	Cardiovascular finding	8.4543E-154
Lipid disorders	Hiatal Hernia	1.02017E-95
Lipid disorders	Menopause	8.65582E-94
Lipid disorders	Hypertensive disease	7.40948E-89
Lipid disorders	Increase in blood pressure	1.76418E-82

Lipid disorders	Edema	4.2154E-78
Lipid disorders	Heart Diseases	8.9441E-76
Lipid disorders	Pain	2.46608E-74
Lipid disorders	Dizziness	3.13701E-61
Lipid disorders	Kidney Diseases	1.21759E-58
Lipid disorders	Obesity	1.36097E-57
Lipid disorders	Scheuermann's Disease	1.78506E-55
Lipid disorders	Heart murmur quality, blowing	9.85936E-48
Lipid disorders	Palpitations	8.52691E-45
Lipid disorders	Cerebral Atherosclerosis	2.0005E-43
Lipid disorders	Heart murmur	4.47225E-42
Lipid disorders	Acrocyanosis	3.40068E-40
Lipid disorders	Fever	1.07859E-38
Lipid disorders	Gynecological history	3.28798E-37
Lipid disorders	Hypercholesterolemia	3.02109E-29
Lipid disorders	Coarctation	4.94557E-27
Lipid disorders	Chronic liver disease	4.42567E-26
Lipid disorders	Unspecified Abortion	4.98661E-25
Lipid disorders	Ischemia	1.11733E-23
Lipid disorders	Premature ventricular contractions	4.26391E-22
Lipid disorders	Dystrophy	2.00729E-21
Lipid disorders	Dyspnea	1.21029E-20
Lipid disorders	Restlessness	3.9924E-20
Lipid disorders	Left Ventricular Hypertrophy	4.66687E-18
Lipid disorders	Cerebrovascular Disorders	7.79467E-18
Lipid disorders	Encephalopathies	6.09402E-16
Lipid disorders	Dyspnea on exertion	3.502E-14
Lipid disorders	Dyslipidemias	4.44097E-14
Lipid disorders	Premature Birth	1.37871E-12
Lipid disorders	Sinus rhythm	1.88394E-12
Lipid disorders	Osteochondrosis	1.09439E-11
Lipid disorders	Heart beat	1.18371E-11
Lipid disorders	Coronary heart disease	9.45891E-10
Lipid disorders	Fibrillation	3.92198E-09
Lipid disorders	Premature Cardiac Complex	5.9903E-09
Neonatal intensive care	Congenital heart disease	0
Neonatal intensive care	Congenital Abnormality	0
Neonatal intensive care	Birth	0
Neonatal intensive care	Diuresis	0
Neonatal intensive care	Ventricular Septal Defects	0
Neonatal intensive care	Pregnancy	0
Neonatal intensive care	Congenital Heart Defects	0
Neonatal intensive care	Systolic Murmurs	0
Neonatal intensive care	Childbirth	0
Neonatal intensive care	Wheezing	0
Neonatal intensive care	Atrial Septal Defects	0

Neonatal intensive care	Color of urine	0
Neonatal intensive care	Surgical wound	0
Neonatal intensive care	Urination	0
Neonatal intensive care	Cyanosis	0
Neonatal intensive care	Respiratory Failure	0
Neonatal intensive care	Newborn	0
Neonatal intensive care	Viral respiratory infection	0
Neonatal intensive care	Air Embolism	0
Neonatal intensive care	Respiration Disorders	0
Neonatal intensive care	Acrocyanosis	0
Neonatal intensive care	Hypoplasia	0
Neonatal intensive care	Hypothermia	0
Neonatal intensive care	Seizures	0
Neonatal intensive care	Weight Gain	0
Neonatal intensive care	Cardiac activity	0
Neonatal intensive care	Muscle hypotonia	0
Neonatal intensive care	Psychomotor development	0
Neonatal intensive care	Liver edge	0
Neonatal intensive care	Hypoxia	0
Neonatal intensive care	Finding of acid-base balance	0
Neonatal intensive care	Surgical fistula	0
Neonatal intensive care	Atelectasis	0
Neonatal intensive care	pathologic fistula	0
Neonatal intensive care	Right Ventricular Hypertrophy	0
Neonatal intensive care	Premature Infant	0
Neonatal intensive care	Sedated state	0
Neonatal intensive care	Hereditary Diseases	0
Neonatal intensive care	Atresia	0
Neonatal intensive care	Oxygen saturation below reference range	0
Neonatal intensive care	Spots on skin	0
Neonatal intensive care	Breast Feeding	0
Neonatal intensive care	Tremor	0
Neonatal intensive care	Dermatologic disorders	0
Neonatal intensive care	Acute Kidney Failure	0
Neonatal intensive care	Acute respiratory disease	0
Neonatal intensive care	Pre-Eclampsia	0
Neonatal intensive care	Ventricular hemorrhage	0
Neonatal intensive care	Duration of gestation	0
Neonatal intensive care	Acidosis	0
Neonatal intensive care	Electrical activity of brain	0
Neonatal intensive care	Icterus	0
Neonatal intensive care	Aspartate transaminase level	0
Neonatal intensive care	Toxemia	0
Neonatal intensive care	Pulmonary Stenosis	0
Neonatal intensive care	Tired	0
Neonatal intensive care	Pharyngitis	0

Neonatal intensive care	Primigravida	0
Neonatal intensive care	Transposition of Great Vessels	0
Neonatal intensive care	Ventricular dilatation	0
Neonatal intensive care	Laboratory test finding	0
Neonatal intensive care	Lactation	0
Neonatal intensive care	Fetal Growth Retardation	0
Neonatal intensive care	Productive cough	0
Neonatal intensive care	Failure to gain weight	0
Neonatal intensive care	Pulmonary Valve Stenosis	0
Neonatal intensive care	Hypoxemia	0
Neonatal intensive care	Psychomotor development	0
Neonatal intensive care	Common ventricle	0
Neonatal intensive care	Heart Septal Defects	0
Neonatal intensive care	Restlessness	0
Neonatal intensive care	Lesion of brain	0
Neonatal intensive care	Invasive arterial pressure	0
Neonatal intensive care	Down Syndrome	0
Neonatal intensive care	Pulpitis	0
Neonatal intensive care	Endocardial Cushion Defects	0
Neonatal intensive care	Stigma	0
Neonatal intensive care	Hypercapnia	0
Neonatal intensive care	Cough reflex	0
Neonatal intensive care	Neonatal Jaundice	0
Neonatal intensive care	Hyporeflexia	0
Neonatal intensive care	Atrioventricular Septal Defect	0
Neonatal intensive care	Necrotizing enterocolitis in fetus OR newborn	0
Neonatal intensive care	Respiratory Acidosis	0
Neonatal intensive care	Pulmonary artery stenosis	0
Neonatal intensive care	Intestinal hemorrhage	0
Neonatal intensive care	Quickly exhausted	0
Neonatal intensive care	Isolated hypoplasia of the right ventricle	0
Neonatal intensive care	Congenital absence	0
Neonatal intensive care	Gas flow	0
Neonatal intensive care	Rickets	0
Neonatal intensive care	Left ventricular hypoplasia	0
Neonatal intensive care	Congenital pneumonia	0
Neonatal intensive care	Right aortic arch	0
Neonatal intensive care	Common atrioventricular canal	0
Neonatal intensive care	Hyaline degeneration	0
Neonatal intensive care	Hypoplastic Left Heart Syndrome	0
Neonatal intensive care	Dilatation of ureter	0
Neonatal intensive care	Common atrium	0
Neonatal intensive care	Urinary tract infection	1.7788E-306
Non-CV encounters	Pregnancy	0
Non-CV encounters	Gynecological history	0
Non-CV encounters	Splenomegaly	0

Non-CV encounters	Exanthema	0
Non-CV encounters	Arthralgia	0
Non-CV encounters	Bleeding tendency	0
Non-CV encounters	Joint swelling	0
Non-CV encounters	Morning stiffness - joint	0
Non-CV encounters	Osteoporosis	4.8194E-251
Non-CV encounters	erythrocyte sedimentation rate result	1.9819E-242
Non-CV encounters	Toxic diffuse goiter	4.7914E-235
Non-CV encounters	Unspecified Abortion	9.3372E-215
Non-CV encounters	Menopause	1.4082E-201
Non-CV encounters	Mean Corpuscular Volume	1.5033E-198
Non-CV encounters	Autoimmune thyroiditis	4.2549E-189
Non-CV encounters	Graves Disease	1.6994E-178
Non-CV encounters	Rheumatism	1.0937E-165
Non-CV encounters	Pulmonary arterial hypertension	3.9686E-158
Non-CV encounters	Rheumatoid Arthritis	6.4228E-156
Non-CV encounters	Thyrotoxicosis	5.7981E-154
Non-CV encounters	Neutropenia	9.1851E-154
Non-CV encounters	Fibroid Tumor	1.1989E-152
Non-CV encounters	Osteopenia	1.458E-152
Non-CV encounters	Multiple Myeloma	4.0844E-147
Non-CV encounters	Plasmacytoma	6.2361E-139
Non-CV encounters	Lymphoma	4.8023E-135
Non-CV encounters	Seropositive rheumatoid arthritis	3.7188E-133
Non-CV encounters	Chronic Lymphocytic Leukemia	6.1121E-125
Non-CV encounters	Lupus Erythematosus	3.2036E-121
Non-CV encounters	Blast Phase	6.4323E-113
Non-CV encounters	Platelet volume	6.6508E-108
Non-CV encounters	Liver edge	2.6218E-106
Non-CV encounters	Erythropoiesis	2.6703E-106
Non-CV encounters	Febrile Neutropenia	2.3977E-105
Non-CV encounters	Toxic multinodular goiter	1.0563E-103
Non-CV encounters	Pathologic Neovascularization	1.4648E-99
Non-CV encounters	Osteochondrosis	4.88408E-98
Non-CV encounters	Granulocyte production	2.58067E-97
Non-CV encounters	Hypothyroidism	1.14872E-95
Non-CV encounters	Uterine Fibroids	2.61017E-93
Non-CV encounters	Pathological fracture	6.02175E-92
Non-CV encounters	Cardiomyopathies	3.13226E-91
Non-CV encounters	Dystrophy	2.4141E-89
Non-CV encounters	Secondary Raynaud's phenomenon	1.42878E-87
Non-CV encounters	Lymphadenopathy	2.53782E-87
Non-CV encounters	Chronic Myeloid Leukemia	1.3519E-86
Non-CV encounters	Spontaneous abortion	2.78592E-86
Non-CV encounters	Arthropathy	1.27511E-85
Non-CV encounters	Patient currently pregnant	2.74599E-84

Non-CV encounters	Pancytopenia	5.13356E-84
Non-CV encounters	Acute cystitis	1.96934E-83
Non-CV encounters	Sclerodactyly	1.28768E-81
Non-CV encounters	Craniocerebral Trauma	6.87491E-81
Non-CV encounters	Menstrual cycle	8.7758E-80
Non-CV encounters	Disorder of eye	2.91535E-76
Non-CV encounters	Hematopoiesis	4.66766E-75
Non-CV encounters	Position of fetus	5.85994E-75
Non-CV encounters	Thrombocytopenia	2.54581E-73
Non-CV encounters	Chronic thromboembolic pulmonary hypertensior	4.6194E-72
Non-CV encounters	Synovitis	4.67684E-72
Non-CV encounters	Scleroderma	5.13584E-69
Non-CV encounters	Inflammatory disease of mucous membrane	1.17833E-68
Non-CV encounters	Mitral Valve Prolapse Syndrome	1.4099E-68
Non-CV encounters	Iron deficiency anemia	3.49254E-68
Non-CV encounters	Thrombophilia, hereditary	3.10432E-67
Non-CV encounters	Goiter	1.46354E-62
Non-CV encounters	Heart beat	1.74977E-60
Non-CV encounters	Leukopenia	3.46423E-60
Non-CV encounters	Myoma	7.36795E-60
Non-CV encounters	Cytopenia	1.17958E-59
Non-CV encounters	Changes in retinal vascular appearance	1.22911E-59
Non-CV encounters	Agranulocytosis	5.70202E-59
Non-CV encounters	Influenza	7.19529E-58
Non-CV encounters	leukemia	1.47138E-56
Non-CV encounters	Antiphospholipid Syndrome	5.77951E-56
Non-CV encounters	Ptosis	2.84078E-54
Non-CV encounters	Muscular Atrophy	4.56272E-53
Non-CV encounters	Contact bleeding	2.82953E-51
Non-CV encounters	Abetalipoproteinemia	3.88987E-51
Non-CV encounters	Iron deficiency	6.75071E-51
Non-CV encounters	Thrombocytosis	1.09552E-50
Non-CV encounters	Hypochromatism	4.17219E-50
Non-CV encounters	Secondary Neoplasm	5.47083E-50
Non-CV encounters	Orthotopic graft	5.86174E-50
Non-CV encounters	Headache	9.09223E-50
Non-CV encounters	Childbirth	1.55035E-49
Non-CV encounters	Telangiectasis	1.60495E-49
Non-CV encounters	Ovarian Cysts	2.69882E-48
Non-CV encounters	Platelet aggregation	3.10668E-48
Non-CV encounters	Palpitations	5.04654E-48
Non-CV encounters	Cushing Syndrome	6.84234E-48
Non-CV encounters	Mydriasis	7.87203E-47
Non-CV encounters	Lymphocytosis	2.65179E-45
Non-CV encounters	Deglutition	3.15719E-45
Non-CV encounters	Cor pulmonale	4.26263E-45

Non-CV encounters	Myopia	4.62463E-45
Non-CV encounters	Rib Fractures	4.97557E-44
Non-CV encounters	Arthritis	3.28112E-43
Non-CV encounters	Compression fracture	5.37681E-43
Non-CV encounters	Anemia	2.27537E-42
Pediatric cardiomyopathy	Cardiac Arrhythmia	0
Pediatric cardiomyopathy	Cardiomyopathies	0
Pediatric cardiomyopathy	Sinus rhythm	0
Pediatric cardiomyopathy	Myocarditis	0
Pediatric cardiomyopathy	Birth	0
Pediatric cardiomyopathy	Pregnancy	0
Pediatric cardiomyopathy	Cardiac conduction	0
Pediatric cardiomyopathy	Microalbuminuria	0
Pediatric cardiomyopathy	Pericarditis	0
Pediatric cardiomyopathy	Childbirth	0
Pediatric cardiomyopathy	Wakefulness	0
Pediatric cardiomyopathy	Viral respiratory infection	0
Pediatric cardiomyopathy	Myocardial dysfunction	0
Pediatric cardiomyopathy	Respiration Disorders	0
Pediatric cardiomyopathy	Systolic Murmurs	0
Pediatric cardiomyopathy	Hereditary Diseases	0
Pediatric cardiomyopathy	Tachycardia	0
Pediatric cardiomyopathy	Cardiac rhythm type	0
Pediatric cardiomyopathy	Sinus Arrhythmia	0
Pediatric cardiomyopathy	Newborn	0
Pediatric cardiomyopathy	Primigravida	0
Pediatric cardiomyopathy	Acute respiratory disease	0
Pediatric cardiomyopathy	Psychomotor development	0
Pediatric cardiomyopathy	Toxemia	0
Pediatric cardiomyopathy	Pre-Eclampsia	0
Pediatric cardiomyopathy	Anuria	0
Pediatric cardiomyopathy	Complete atrioventricular block	0
Pediatric cardiomyopathy	Carditis	0
Pediatric cardiomyopathy	Anemia of chronic disease	0
Pediatric cardiomyopathy	Chronic myocarditis	0
Pediatric cardiomyopathy	Primary Cardiomyopathies	0
Pediatric cardiomyopathy	Acute myocarditis	0
Pediatric cardiomyopathy	Disease due to Parvoviridae	0
Pediatric cardiomyopathy	Bradyarrhythmia	0
Pediatric cardiomyopathy	Premature Birth	1.3789E-282
Pediatric cardiomyopathy	Viral myocarditis	3.2616E-274
Pediatric cardiomyopathy	Esophageal Diseases	7.6757E-263
Pediatric cardiomyopathy	Acclimatization	2.7207E-261
Pediatric cardiomyopathy	Bradycardia	7.1376E-256
Pediatric cardiomyopathy	Electrical activity of brain	2.3278E-255
Pediatric cardiomyopathy	Left ventricular dilatation	4.3523E-246

Pediatric cardiomyopathy	Tidal Volume	6.0608E-232
Pediatric cardiomyopathy	Liver edge	7.0154E-227
Pediatric cardiomyopathy	Fatigue	8.0854E-223
Pediatric cardiomyopathy	Shock	8.6868E-220
Pediatric cardiomyopathy	Breast Feeding	4.4198E-217
Pediatric cardiomyopathy	Supraventricular tachycardia	2.7411E-216
Pediatric cardiomyopathy	Tonsillitis	1.0761E-202
Pediatric cardiomyopathy	Atopic dermatitis	3.8304E-200
Pediatric cardiomyopathy	Hernia	2.6367E-184
Pediatric cardiomyopathy	Auriculo-Ventricular Dissociation	5.9534E-177
Pediatric cardiomyopathy	Rickets	3.0115E-175
Pediatric cardiomyopathy	Atrioventricular junctional rhythm	7.0164E-173
Pediatric cardiomyopathy	Hypertrophy	2.0756E-172
Pediatric cardiomyopathy	Atrioventricular Block	9.5296E-166
Pediatric cardiomyopathy	Atrial rhythm	4.9794E-150
Pediatric cardiomyopathy	Paroxysmal tachycardia	2.0656E-139
Pediatric cardiomyopathy	Decrease in appetite	8.353E-139
Pediatric cardiomyopathy	Myopia	3.1754E-136
Pediatric cardiomyopathy	Ventricular arrhythmia	2.6132E-134
Pediatric cardiomyopathy	Underweight	1.8951E-125
Pediatric cardiomyopathy	Weight Gain	5.6567E-125
Pediatric cardiomyopathy	Increased sweating	3.833E-117
Pediatric cardiomyopathy	Collapse	2.783E-110
Pediatric cardiomyopathy	Hiatal Hernia	9.8796E-110
Pediatric cardiomyopathy	Metabolic Diseases	5.5788E-109
Pediatric cardiomyopathy	Rhinitis	1.1037E-104
Pediatric cardiomyopathy	Traumatic injury	6.6759E-104
Pediatric cardiomyopathy	Restrictive cardiomyopathy	9.6262E-102
Pediatric cardiomyopathy	Vomiting	3.34792E-91
Pediatric cardiomyopathy	Electrocardiogram axis finding	2.7899E-86
Pediatric cardiomyopathy	Fetal Growth Retardation	3.40332E-85
Pediatric cardiomyopathy	Hypoxia	7.88738E-83
Pediatric cardiomyopathy	Finding of creatine kinase level	1.65833E-82
Pediatric cardiomyopathy	Mycoplasma Infections	8.3602E-80
Pediatric cardiomyopathy	Anemia	8.73857E-79
Pediatric cardiomyopathy	Failure to gain weight	8.0064E-76
Pediatric cardiomyopathy	Muscle relaxation phase	3.71964E-74
Pediatric cardiomyopathy	Acute bronchitis	1.67599E-73
Pediatric cardiomyopathy	Vaginitis	7.65127E-70
Pediatric cardiomyopathy	Protein level	3.89968E-69
Pediatric cardiomyopathy	ST segment	1.23971E-68
Pediatric cardiomyopathy	Conduction disorder of the heart	6.91373E-68
Pediatric cardiomyopathy	Atrial tachycardia	5.47038E-67
Pediatric cardiomyopathy	Polymorphic ventricular tachycardia	1.04996E-66
Pediatric cardiomyopathy	Cardiac dyskinesia	1.72091E-61
Pediatric cardiomyopathy	Rhinorrhea	5.4073E-61

Pediatric cardiomyopathy	Valgus deformity	3.73861E-60
Pediatric cardiomyopathy	furuncle	4.03566E-58
Pediatric cardiomyopathy	Esophageal Hernia	6.32313E-57
Pediatric cardiomyopathy	Lymphocytic myocarditis	7.72599E-53
Pediatric cardiomyopathy	Chronic tonsillitis	4.43006E-52
Pediatric cardiomyopathy	pathologic cytolysis	5.04123E-52
Pediatric cardiomyopathy	Scoliosis	9.69736E-51
Pediatric cardiomyopathy	Recurrent ventricular tachycardia	1.64096E-45
Pediatric cardiomyopathy	Premature Cardiac Complex	5.53182E-45
Pediatric cardiomyopathy	Ear Inflammation	9.08162E-45
Pediatric cardiomyopathy	Strabismus	2.641E-43
Pediatric cardiomyopathy	Duration of gestation	2.56256E-42
Pediatric cardiomyopathy	Diuresis	2.1903E-41

Prevelance in cluster	Prevalence outside cluster
96.00%	52.70%
92.70%	38.00%
87.60%	35.00%
80.40%	34.70%
78.50%	33.30%
77.60%	34.20%
76.60%	27.10%
75.70%	37.10%
73.80%	23.90%
62.60%	7.20%
61.90%	15.90%
59.20%	4.73%
59.10%	15.40%
56.20%	10.90%
50.00%	15.40%
49.90%	11.40%
49.20%	14.80%
45.60%	15.20%
43.30%	16.30%
41.10%	10.60%
37.20%	13.80%
36.30%	9.20%
32.80%	7.12%
31.20%	9.86%
30.00%	8.42%
28.50%	6.76%
27.70%	4.22%
23.80%	7.59%
22.90%	2.28%
21.50%	6.06%
21.20%	2.29%
17.80%	2.24%
16.60%	4.27%
10.60%	0.76%
7.36%	1.30%
22.70%	8.07%
30.70%	9.31%
14.10%	3.54%
34.60%	11.50%
25.00%	9.96%
3.42%	0.39%
13.20%	3.46%
82.60%	46.50%
85.00%	42.00%
14.10%	3.91%

13.50%	3.80%
22.90%	8.06%
7.13%	1.38%
12.50%	3.82%
12.10%	3.94%
4.42%	0.56%
6.78%	0.96%
36.30%	16.80%
44.80%	16.80%
14.50%	3.70%
8.28%	2.22%
11.00%	3.66%
30.40%	13.20%
13.10%	4.87%
10.90%	3.30%
20.30%	7.68%
15.50%	4.58%
2.77%	0.41%
5.61%	1.74%
16.10%	6.35%
43.10%	22.90%
2.71%	0.51%
8.22%	2.54%
30.40%	12.00%
16.90%	5.20%
50.90%	23.50%
71.50%	37.30%
28.60%	12.30%
6.28%	1.90%
13.70%	4.13%
30.70%	12.80%
18.70%	6.62%
39.40%	23.60%
51.00%	30.00%
17.10%	5.60%
16.00%	6.16%
93.60%	68.50%
26.70%	12.80%
12.30%	4.49%
1.85%	0.41%
3.33%	0.68%
5.40%	1.91%
8.32%	2.52%
2.37%	0.41%
12.00%	3.75%
8.97%	3.09%

2.98%	0.63%
7.78%	3.02%
17.10%	7.91%
8.97%	5.05%
12.30%	5.38%
5.88%	2.35%
2.83%	0.63%
18.40%	8.14%
39.10%	17.70%
88.60%	48.00%
84.50%	42.70%
84.10%	46.50%
81.60%	37.30%
73.40%	38.20%
62.30%	25.20%
61.80%	30.00%
59.40%	25.00%
55.00%	19.30%
49.40%	16.30%
46.20%	14.50%
43.80%	7.58%
39.50%	12.20%
35.30%	12.00%
33.40%	4.84%
32.40%	9.88%
31.40%	11.50%
30.00%	5.41%
29.80%	10.30%
27.20%	6.65%
27.10%	5.05%
27.10%	7.47%
26.90%	3.98%
26.90%	9.96%
25.90%	8.07%
25.40%	6.09%
23.00%	4.22%
22.30%	4.11%
21.50%	7.59%
19.60%	5.55%
17.80%	2.42%
17.40%	4.42%
16.30%	3.32%
14.00%	2.54%
13.40%	1.99%
13.30%	2.82%
11.30%	1.74%

11.30%	2.58%
10.90%	2.00%
10.20%	1.84%
8.37%	1.47%
7.76%	1.34%
6.87%	0.87%
6.73%	1.18%
6.65%	0.88%
6.30%	0.82%
6.29%	1.23%
6.02%	0.73%
5.84%	1.22%
5.73%	0.79%
5.35%	0.82%
4.74%	0.68%
4.28%	0.74%
3.92%	0.60%
3.87%	0.65%
3.69%	0.61%
2.47%	0.31%
8.23%	1.83%
8.22%	1.74%
15.40%	5.57%
21.40%	8.01%
25.60%	8.75%
5.89%	1.31%
4.76%	0.97%
17.70%	6.14%
4.33%	0.70%
13.90%	3.74%
11.70%	2.89%
12.50%	4.27%
22.60%	8.23%
2.20%	0.31%
10.80%	2.83%
6.77%	1.44%
74.20%	37.30%
18.00%	5.60%
2.78%	0.45%
6.15%	1.29%
7.34%	1.54%
6.77%	1.53%
2.06%	0.28%
19.80%	6.76%
39.00%	15.40%
34.00%	12.60%

2.80%	0.42%
5.06%	1.02%
2.32%	0.31%
8.39%	2.12%
2.94%	0.53%
3.29%	0.44%
11.70%	3.66%
12.10%	3.82%
5.16%	1.02%
14.10%	4.25%
13.40%	4.14%
3.46%	0.59%
1.11%	0.12%
1.65%	0.20%
1.63%	0.20%
12.20%	3.64%
95.50%	68.70%
99.70%	9.11%
71.40%	34.20%
31.60%	8.75%
18.20%	1.37%
13.10%	0.76%
12.70%	0.91%
11.10%	0.82%
16.30%	3.95%
38.00%	15.40%
13.90%	4.16%
2.03%	0.21%
2.29%	0.26%
58.80%	38.00%
75.10%	52.70%
51.90%	34.70%
1.37%	0.18%
11.30%	5.60%
2.14%	0.50%
14.00%	7.12%
1.64%	0.27%
34.30%	23.60%
2.29%	0.88%
1.34%	0.64%
5.43%	2.11%
4.95%	1.66%
0.86%	0.23%
17.80%	10.90%
57.60%	42.00%
27.00%	18.60%

80.00%	68.70%
32.00%	22.90%
11.20%	6.35%
54.10%	42.70%
4.15%	1.76%
18.60%	12.20%
12.00%	7.26%
18.60%	12.10%
2.68%	1.37%
0.71%	0.11%
14.40%	8.14%
3.04%	1.06%
80.20%	68.50%
0.80%	0.23%
0.59%	0.18%
3.25%	1.74%
8.00%	5.05%
3.10%	1.53%
10.30%	7.12%
15.40%	11.80%
11.00%	7.19%
45.40%	34.70%
0.62%	0.20%
18.60%	13.80%
1.55%	0.64%
0.59%	0.22%
0.68%	0.26%
1.25%	0.63%
14.30%	10.70%
1.22%	0.52%
94.80%	11.00%
75.50%	34.70%
61.20%	18.60%
57.20%	13.30%
32.20%	10.10%
26.70%	3.06%
26.50%	3.02%
22.70%	2.49%
18.60%	2.25%
15.10%	4.16%
14.00%	3.16%
12.70%	2.16%
6.13%	0.62%
5.09%	0.54%
12.10%	3.28%
21.70%	9.49%

10.00%	2.38%
7.77%	2.41%
34.70%	19.30%
32.00%	18.20%
2.14%	0.27%
44.30%	28.00%
61.10%	42.70%
19.90%	10.00%
15.60%	6.07%
2.05%	0.29%
11.50%	5.54%
17.40%	8.30%
2.85%	0.71%
2.18%	0.59%
45.90%	33.30%
8.88%	4.56%
25.50%	17.90%
17.30%	9.89%
51.40%	37.30%
3.55%	1.17%
83.20%	68.70%
4.49%	1.69%
1.30%	0.33%
1.58%	0.40%
81.30%	68.50%
31.50%	23.60%
45.20%	34.70%
1.21%	0.31%
17.30%	12.00%
17.10%	12.20%
16.60%	11.90%
0.80%	0.21%
13.70%	9.29%
1.87%	0.73%
1.87%	0.57%
9.97%	6.63%
10.50%	6.83%
23.90%	16.10%
28.50%	22.80%
1.97%	0.95%
1.75%	0.75%
5.93%	3.86%
17.00%	13.20%
14.90%	12.00%
57.30%	48.00%
11.90%	10.00%

24.40%	17.70%
0.54%	0.14%
4.08%	2.29%
1.32%	0.61%
2.71%	2.11%
55.50%	44.80%
1.48%	0.67%
3.57%	2.11%
4.00%	2.45%
1.07%	0.49%
14.60%	11.80%
9.35%	7.12%
34.60%	30.20%
0.56%	0.22%
1.28%	0.65%
9.08%	6.75%
89.10%	5.40%
87.40%	25.20%
43.10%	12.00%
39.80%	7.47%
20.70%	3.21%
14.70%	3.33%
13.50%	2.35%
8.61%	1.18%
18.30%	6.96%
19.50%	7.32%
6.01%	1.22%
7.10%	2.09%
10.30%	4.13%
80.90%	68.70%
25.90%	17.20%
18.80%	9.29%
7.87%	2.89%
3.96%	0.91%
2.51%	0.45%
38.00%	25.80%
48.60%	37.30%
7.70%	3.25%
31.30%	22.80%
78.70%	68.50%
40.00%	27.10%
17.30%	8.75%
56.40%	48.00%
8.06%	3.79%
9.81%	4.65%
5.05%	2.21%

19.60%	12.60%
44.70%	38.20%
22.80%	16.30%
1.01%	0.23%
17.40%	12.20%
1.88%	0.72%
7.18%	3.07%
2.10%	0.79%
2.29%	0.84%
14.40%	9.31%
2.10%	1.09%
16.10%	12.20%
1.94%	0.89%
16.30%	13.00%
16.60%	13.20%
11.70%	8.13%
3.99%	2.48%
31.00%	30.00%
8.72%	6.65%
4.31%	3.16%
5.68%	4.24%
13.60%	10.60%
3.49%	1.62%
5.60%	5.36%
1.25%	0.54%
14.40%	11.90%
2.81%	1.96%
55.60%	54.60%
81.30%	6.20%
81.20%	68.70%
47.40%	19.90%
44.30%	16.70%
14.20%	1.82%
15.40%	5.02%
6.78%	2.28%
3.25%	0.58%
66.10%	52.70%
11.40%	6.09%
43.70%	34.70%
4.49%	1.49%
6.57%	3.64%
31.50%	22.90%
75.20%	68.50%
36.40%	33.10%
5.33%	2.53%
28.80%	22.80%

48.20%	50.10%
16.60%	11.90%
3.22%	1.66%
16.90%	12.10%
30.60%	25.80%
2.98%	1.45%
23.40%	21.30%
14.00%	12.80%
51.10%	54.60%
9.02%	6.65%
3.08%	1.62%
5.47%	3.12%
41.00%	38.00%
29.10%	25.20%
0.90%	0.27%
4.43%	2.90%
9.39%	7.12%
12.20%	11.80%
2.04%	1.05%
6.17%	4.65%
6.57%	5.35%
19.60%	18.20%
1.04%	0.44%
13.70%	12.20%
1.24%	0.69%
21.20%	19.30%
0.93%	0.46%
1.77%	1.25%
5.37%	3.79%
14.10%	12.20%
1.77%	0.90%
38.20%	42.70%
11.80%	10.70%
1.34%	0.74%
6.51%	5.48%
38.30%	34.20%
10.90%	9.29%
3.52%	2.47%
98.50%	37.10%
96.40%	30.20%
91.60%	6.01%
88.70%	68.50%
44.60%	12.20%
40.30%	18.20%
39.20%	22.80%
24.50%	6.83%

22.30%	5.48%
17.80%	5.35%
3.45%	0.28%
73.30%	52.70%
25.30%	12.90%
18.60%	6.20%
63.80%	50.10%
8.55%	2.46%
4.49%	0.88%
86.50%	68.70%
7.27%	2.95%
46.00%	33.10%
17.40%	10.10%
8.17%	3.49%
18.30%	9.29%
19.90%	11.90%
54.90%	42.00%
4.11%	1.48%
11.90%	6.26%
28.30%	19.30%
36.10%	25.80%
43.10%	34.70%
4.58%	1.83%
16.20%	10.70%
11.70%	6.26%
1.88%	0.64%
16.70%	13.20%
17.10%	13.00%
5.59%	3.07%
4.95%	2.67%
56.60%	54.60%
10.10%	7.32%
10.20%	7.19%
21.20%	17.90%
13.20%	9.94%
1.07%	0.22%
11.60%	9.20%
43.80%	46.50%
25.00%	21.30%
52.10%	48.00%
14.40%	12.10%
41.30%	38.00%
9.10%	6.87%
2.05%	1.25%
10.40%	8.14%
10.30%	8.13%

1.91%	1.01%
5.65%	5.47%
2.08%	2.04%
1.21%	0.76%
2.95%	2.39%
3.85%	3.21%
89.30%	68.50%
84.00%	52.70%
83.70%	68.70%
81.50%	42.00%
58.60%	34.20%
56.70%	34.70%
53.00%	38.00%
32.00%	22.90%
30.40%	18.20%
30.00%	19.30%
25.10%	12.20%
13.70%	7.19%
24.20%	17.90%
32.70%	25.80%
12.00%	7.12%
16.10%	10.70%
16.90%	11.90%
11.80%	7.26%
9.63%	6.62%
16.20%	12.10%
26.50%	22.80%
8.85%	5.48%
33.20%	33.10%
12.60%	9.29%
11.40%	8.14%
36.20%	37.10%
3.83%	2.11%
10.90%	9.20%
7.46%	5.35%
11.40%	10.90%
1.33%	0.45%
21.50%	21.30%
3.06%	1.76%
4.92%	3.54%
4.97%	4.56%
14.50%	14.80%
43.70%	50.10%
1.37%	0.76%
22.20%	23.60%
11.00%	9.86%

7.69%	6.26%
0.85%	0.41%
0.91%	0.41%
11.80%	12.00%
3.21%	2.48%
7.02%	6.74%
4.71%	4.46%
26.40%	30.20%
7.01%	6.83%
2.92%	2.45%
2.83%	2.48%
4.53%	4.16%
2.98%	2.79%
2.39%	1.96%
3.03%	2.59%
2.89%	2.60%
1.18%	0.80%
0.82%	0.44%
4.76%	4.87%
2.53%	2.21%
44.90%	54.60%
4.28%	4.28%
0.42%	0.20%
11.70%	13.20%
4.30%	4.24%
4.07%	4.16%
0.52%	0.33%
0.40%	0.27%
95.80%	52.70%
90.80%	68.50%
88.40%	42.00%
77.40%	34.20%
76.30%	38.00%
73.80%	34.70%
67.20%	46.50%
58.80%	23.50%
54.70%	34.70%
50.60%	23.60%
49.50%	15.40%
48.10%	15.90%
34.30%	9.86%
33.00%	8.14%
32.80%	13.80%
31.50%	12.90%
28.80%	16.80%
24.60%	10.90%

23.70%	7.12%
23.00%	9.20%
20.40%	8.42%
15.20%	6.06%
13.80%	3.54%
13.20%	3.46%
23.70%	13.20%
4.94%	1.21%
9.93%	3.95%
22.30%	16.30%
25.40%	12.80%
27.00%	14.80%
51.40%	33.10%
20.70%	11.40%
68.10%	50.10%
8.09%	3.70%
2.59%	0.50%
8.77%	3.80%
62.80%	54.60%
8.56%	5.38%
6.33%	3.30%
12.80%	6.62%
21.30%	15.20%
7.70%	5.05%
12.30%	6.74%
6.80%	3.54%
47.00%	37.10%
6.74%	4.58%
32.30%	21.30%
48.30%	33.30%
37.60%	30.20%
3.92%	1.60%
6.97%	4.22%
32.50%	25.80%
2.56%	0.96%
6.64%	4.49%
28.60%	22.90%
3.09%	1.38%
8.76%	7.26%
1.22%	0.41%
51.90%	48.00%
10.90%	7.91%
21.80%	19.30%
0.98%	0.37%
1.07%	0.31%
11.00%	6.87%

1.40%	0.55%
1.12%	0.40%
0.90%	0.27%
1.31%	0.56%
1.25%	0.52%
5.42%	4.16%
1.75%	0.95%
0.77%	0.28%
2.27%	1.21%
9.15%	7.26%
3.89%	2.59%
14.20%	12.80%
1.22%	0.64%
8.36%	6.16%
5.71%	4.87%
0.90%	0.36%
9.61%	8.06%
1.01%	0.51%
12.00%	9.82%
0.54%	0.15%
8.68%	7.19%
1.78%	1.22%
13.10%	10.70%
3.12%	2.29%
1.48%	0.76%
12.80%	12.10%
8.00%	6.63%
0.69%	0.33%
1.92%	1.32%
2.77%	2.24%
96.40%	33.30%
94.30%	35.00%
92.20%	25.00%
92.00%	12.30%
90.20%	30.00%
89.30%	15.20%
86.70%	8.27%
86.00%	34.70%
85.40%	6.29%
83.10%	42.00%
83.00%	34.70%
80.20%	16.80%
75.20%	28.00%
73.30%	6.75%
70.80%	7.21%
61.30%	23.60%

60.40%	7.05%
60.10%	12.00%
59.90%	18.20%
54.40%	3.66%
47.10%	13.80%
46.40%	17.20%
39.20%	8.01%
38.10%	4.56%
37.90%	9.82%
33.30%	10.30%
33.10%	5.36%
32.00%	7.68%
31.50%	6.62%
30.70%	5.57%
29.80%	2.68%
27.70%	5.05%
27.50%	7.12%
26.30%	2.70%
25.10%	3.74%
22.90%	5.57%
19.50%	1.39%
17.00%	1.44%
15.50%	2.19%
15.20%	3.77%
14.80%	3.82%
14.00%	3.91%
13.70%	1.29%
12.70%	3.74%
10.20%	1.55%
8.18%	1.25%
6.01%	0.61%
11.90%	2.67%
6.03%	1.07%
10.10%	1.75%
9.74%	2.00%
25.70%	6.83%
42.50%	16.30%
27.50%	7.82%
15.10%	3.28%
11.60%	2.18%
6.09%	0.85%
10.50%	2.43%
75.90%	38.00%
67.30%	30.20%
10.70%	2.59%
30.80%	9.86%

20.60%	5.34%
19.90%	6.16%
10.30%	2.49%
49.40%	22.90%
8.42%	2.35%
20.50%	8.07%
2.78%	0.34%
90.10%	44.80%
80.40%	37.10%
20.30%	6.06%
2.23%	0.30%
3.12%	0.38%
14.30%	4.54%
23.80%	8.42%
2.63%	0.33%
8.29%	2.54%
47.30%	18.60%
1.99%	0.23%
7.56%	2.17%
10.20%	3.94%
33.60%	13.30%
17.70%	7.59%
23.50%	8.30%
3.51%	0.60%
13.70%	4.62%
2.31%	0.29%
9.87%	2.29%
29.60%	13.20%
24.90%	8.43%
3.32%	0.66%
3.63%	0.75%
14.80%	6.14%
26.10%	10.90%
2.61%	0.53%
8.72%	2.57%
8.72%	3.02%
19.20%	7.20%
16.40%	7.12%
93.90%	4.57%
84.70%	4.15%
82.00%	17.90%
64.70%	3.22%
55.20%	2.44%
49.20%	2.59%
47.90%	2.06%
46.60%	1.99%

43.70%	13.00%
42.50%	6.83%
41.80%	1.54%
32.50%	1.19%
32.30%	6.71%
20.60%	0.92%
19.80%	1.82%
18.20%	1.74%
17.80%	2.48%
15.10%	0.62%
11.00%	1.09%
10.50%	1.07%
9.84%	1.16%
9.14%	0.45%
8.83%	0.79%
7.81%	0.45%
7.60%	0.66%
7.60%	0.68%
7.01%	0.81%
6.55%	0.55%
6.23%	0.31%
5.92%	0.32%
5.36%	0.34%
4.94%	0.21%
4.03%	0.27%
3.75%	0.19%
3.22%	0.13%
4.52%	0.32%
4.41%	0.28%
13.50%	1.46%
4.20%	0.27%
10.80%	1.48%
11.00%	1.76%
12.50%	2.33%
12.70%	2.26%
5.36%	0.51%
6.51%	0.63%
5.81%	0.51%
2.17%	0.17%
68.30%	44.80%
3.61%	0.25%
2.80%	0.18%
4.27%	0.33%
6.41%	0.74%
70.90%	34.70%
2.83%	0.26%

5.46%	0.85%
3.89%	0.40%
5.01%	0.62%
6.13%	1.09%
3.29%	0.30%
2.41%	0.19%
4.13%	0.54%
1.75%	0.11%
7.22%	0.98%
3.36%	0.36%
5.53%	0.60%
58.40%	34.70%
13.60%	3.52%
5.71%	0.89%
3.57%	0.49%
6.44%	1.60%
2.59%	0.33%
3.32%	0.59%
2.31%	0.23%
1.64%	0.13%
5.43%	1.05%
1.82%	0.19%
18.10%	6.74%
1.96%	0.17%
3.50%	0.73%
5.08%	0.88%
5.88%	1.18%
2.20%	0.32%
1.15%	0.12%
8.62%	2.46%
5.04%	1.78%
15.60%	6.26%
10.50%	3.27%
1.99%	0.23%
17.90%	6.19%
2.34%	0.39%
5.04%	1.04%
4.55%	0.88%
23.40%	8.43%
2.80%	0.31%
4.13%	1.19%
11.50%	3.54%
1.54%	0.23%
7.85%	2.11%
2.06%	0.37%
7.50%	2.21%

100.00%	5.07%
90.80%	10.00%
51.70%	6.59%
43.10%	5.54%
17.30%	2.67%
14.10%	2.71%
9.98%	1.08%
32.50%	12.80%
10.00%	1.86%
34.90%	13.70%
19.20%	6.75%
9.83%	3.33%
23.80%	9.89%
18.20%	6.04%
8.86%	1.76%
2.44%	0.34%
2.08%	0.28%
4.99%	0.88%
8.25%	2.04%
18.30%	8.23%
1.52%	0.16%
6.26%	1.76%
2.34%	0.42%
2.70%	0.40%
2.65%	0.45%
29.20%	19.30%
5.30%	1.37%
4.63%	1.09%
21.50%	9.49%
2.49%	0.47%
10.00%	3.68%
4.38%	0.84%
1.37%	0.24%
6.62%	2.39%
26.00%	16.80%
3.31%	0.79%
1.17%	0.20%
1.42%	0.38%
2.44%	0.83%
3.21%	0.97%
7.84%	4.14%
6.77%	2.03%
14.10%	6.09%
15.50%	12.30%
3.21%	1.01%
3.87%	1.82%

2.95%	0.78%
1.68%	0.42%
2.14%	0.80%
3.16%	1.14%
2.08%	0.53%
2.65%	1.07%
1.42%	0.32%
1.42%	0.44%
24.60%	16.70%
24.30%	25.00%
1.07%	0.38%
6.82%	3.71%
1.17%	0.26%
2.14%	0.95%
0.76%	0.23%
1.58%	0.45%
0.76%	0.35%
1.12%	0.30%
1.01%	0.42%
0.50%	0.14%
1.78%	1.08%
1.63%	0.79%
2.24%	1.24%
0.66%	0.23%
0.81%	0.41%
1.88%	0.86%
90.70%	19.30%
77.90%	12.10%
66.10%	3.00%
48.40%	2.38%
45.20%	2.30%
30.10%	2.02%
24.40%	4.42%
24.10%	3.52%
22.70%	3.31%
20.90%	3.27%
20.60%	0.67%
20.40%	0.97%
20.30%	3.64%
19.90%	3.94%
17.10%	0.69%
16.30%	1.19%
15.70%	0.58%
15.70%	1.60%
13.90%	1.06%
11.00%	1.09%

8.64%	0.38%
7.89%	0.28%
7.61%	0.35%
7.11%	0.40%
6.75%	0.23%
4.44%	0.22%
3.59%	0.13%
12.00%	1.04%
58.50%	25.80%
18.10%	2.46%
7.04%	0.45%
3.34%	0.31%
20.90%	7.59%
4.58%	0.53%
9.35%	1.70%
20.00%	7.58%
7.53%	1.13%
7.14%	1.24%
2.41%	0.16%
2.31%	0.28%
10.40%	2.90%
5.47%	1.18%
3.48%	0.44%
2.98%	0.28%
3.55%	0.47%
49.60%	26.20%
8.67%	1.89%
5.97%	0.88%
3.98%	0.74%
10.80%	2.48%
10.70%	4.16%
6.00%	1.45%
2.16%	0.29%
3.41%	0.98%
3.37%	0.37%
2.59%	0.29%
7.82%	2.67%
4.08%	0.89%
2.95%	0.46%
4.30%	1.01%
1.42%	0.22%
6.43%	1.76%
1.56%	0.17%
16.70%	8.13%
7.14%	2.04%
1.95%	0.24%

3.09%	0.67%
2.13%	0.51%
2.02%	0.29%
0.81%	0.27%
2.63%	0.49%
22.60%	9.94%
1.99%	0.48%
16.60%	7.46%
3.59%	1.75%
7.53%	3.74%
6.15%	2.28%
2.59%	0.59%
0.92%	0.16%
2.77%	0.80%
6.29%	2.96%
2.70%	0.80%
0.78%	0.11%
1.35%	0.78%
1.03%	0.21%
2.48%	0.91%
7.04%	3.79%
0.85%	0.15%
1.20%	0.27%
34.00%	22.90%
2.27%	0.54%
1.03%	0.22%
4.16%	1.87%
12.40%	8.07%
1.06%	0.29%
9.77%	5.06%
0.71%	0.22%
4.23%	2.28%
2.45%	0.60%
1.63%	0.63%
96.40%	8.31%
92.90%	68.70%
63.00%	13.20%
62.70%	42.70%
29.00%	12.20%
21.60%	6.09%
20.20%	4.77%
18.00%	6.65%
28.30%	16.70%
30.30%	19.90%
61.90%	48.00%
8.49%	1.82%

6.29%	2.28%
9.27%	4.22%
46.50%	37.30%
37.40%	23.50%
6.52%	2.53%
10.60%	5.02%
32.70%	25.20%
9.69%	5.05%
47.60%	37.30%
56.40%	50.10%
34.50%	25.80%
2.62%	0.58%
2.67%	0.72%
18.70%	12.10%
41.40%	33.10%
6.10%	2.82%
3.94%	1.62%
9.25%	5.60%
7.13%	3.98%
8.71%	4.84%
7.20%	4.11%
25.00%	19.30%
7.41%	5.55%
2.60%	1.15%
3.70%	1.54%
1.29%	0.26%
3.09%	1.34%
1.52%	0.36%
4.13%	2.20%
14.80%	12.80%
17.40%	14.50%
2.98%	1.22%
2.95%	1.53%
5.35%	3.12%
2.51%	1.29%
55.80%	54.60%
4.48%	2.89%
24.80%	22.80%
1.15%	0.41%
9.81%	7.58%
3.52%	1.84%
2.41%	0.89%
10.60%	8.23%
2.06%	1.02%
1.85%	1.12%
0.63%	0.29%

24.70%	21.30%
2.13%	1.25%
6.78%	3.45%
2.30%	1.49%
6.45%	5.41%
37.30%	38.20%
6.76%	5.06%
1.08%	0.64%
0.72%	0.27%
1.10%	0.42%
0.98%	0.42%
4.29%	3.21%
1.43%	0.64%
1.00%	0.53%
5.28%	3.79%
1.45%	0.82%
10.60%	9.49%
1.10%	0.45%
6.15%	5.47%
0.93%	0.40%
10.80%	11.40%
1.15%	0.54%
1.47%	0.79%
8.85%	7.47%
3.54%	2.60%
0.56%	0.22%
1.15%	0.56%
2.01%	1.45%
0.37%	0.16%
1.29%	0.87%
5.00%	4.42%
1.26%	0.84%
44.40%	46.50%
1.10%	0.65%
1.64%	1.18%
2.93%	2.54%
95.90%	42.70%
95.80%	37.30%
95.40%	44.80%
78.70%	16.10%
73.80%	17.70%
53.20%	18.60%
53.20%	23.50%
50.90%	19.30%
32.30%	13.30%
27.80%	15.40%

24.80%	3.45%
24.70%	12.00%
23.80%	6.07%
14.40%	8.30%
13.70%	2.38%
5.54%	1.06%
28.50%	28.00%
30.70%	33.30%
8.11%	5.60%
6.17%	3.95%
16.90%	11.00%
4.64%	2.16%
5.21%	3.71%
9.82%	9.82%
0.83%	0.45%
0.65%	0.29%
1.10%	0.84%
19.70%	25.20%
5.57%	8.75%
2.18%	3.28%
0.83%	0.62%
4.34%	6.09%
0.80%	0.64%
1.04%	1.08%
0.35%	0.26%
94.50%	27.10%
87.30%	35.00%
87.00%	37.30%
82.50%	23.90%
69.60%	16.80%
60.20%	15.40%
46.30%	11.50%
43.80%	9.31%
36.70%	10.60%
35.50%	12.60%
23.40%	6.76%
21.80%	4.87%
19.70%	3.75%
16.10%	4.58%
14.50%	3.09%
7.12%	1.66%
25.70%	12.00%
14.00%	4.01%
8.21%	2.68%
2.97%	0.49%
15.50%	6.50%

4.35%	1.56%
2.35%	0.73%
22.60%	12.20%
2.50%	0.81%
5.44%	1.92%
2.47%	0.60%
23.90%	16.30%
1.70%	0.33%
2.17%	0.42%
2.06%	0.58%
22.60%	17.20%
2.94%	1.09%
12.20%	7.32%
1.03%	0.20%
6.91%	4.13%
0.91%	0.26%
1.20%	0.33%
9.56%	7.26%
1.05%	0.30%
1.11%	0.39%
0.85%	0.20%
3.82%	1.62%
1.82%	0.81%
17.80%	15.90%
10.70%	6.96%
1.67%	0.70%
2.11%	1.22%
0.85%	0.28%
1.82%	0.63%
2.64%	1.18%
1.53%	0.79%
8.68%	6.16%
0.73%	0.26%
3.29%	2.52%
6.15%	4.62%
2.08%	1.04%
10.90%	9.88%
4.26%	2.21%
1.47%	0.60%
100.00%	5.20%
100.00%	5.60%
98.60%	27.10%
84.10%	37.30%
76.80%	35.00%
76.40%	23.90%
75.10%	16.80%

49.00%	9.31%
34.40%	11.50%
32.40%	10.60%
24.00%	4.87%
16.70%	3.75%
17.20%	6.76%
28.80%	12.60%
4.06%	0.49%
24.10%	12.00%
27.30%	15.90%
2.78%	0.73%
20.90%	15.40%
10.00%	4.58%
8.80%	4.01%
12.00%	7.26%
54.90%	48.00%
10.80%	6.50%
23.90%	16.10%
27.20%	25.20%
3.01%	1.66%
2.23%	1.04%
23.50%	17.70%
0.73%	0.26%
4.60%	3.09%
1.91%	0.52%
2.09%	1.22%
92.20%	34.70%
79.90%	33.30%
76.40%	44.80%
69.20%	8.43%
65.20%	6.19%
38.20%	9.82%
24.00%	6.63%
18.10%	3.78%
12.20%	0.80%
3.95%	0.48%
59.70%	34.70%
4.24%	0.57%
38.90%	26.20%
4.80%	0.68%
11.80%	3.56%
28.80%	13.30%
7.23%	2.54%
20.30%	8.14%
11.60%	4.16%
2.82%	0.33%

10.30%	5.39%
11.10%	4.28%
4.24%	0.80%
2.50%	0.89%
7.71%	2.11%
2.70%	0.96%
6.90%	2.78%
5.45%	1.76%
4.16%	1.67%
1.37%	0.22%
1.29%	0.36%
6.38%	2.46%
11.30%	6.87%
1.29%	0.29%
1.49%	0.38%
35.90%	30.20%
4.80%	1.89%
1.69%	0.51%
3.95%	1.70%
1.73%	0.49%
1.89%	0.79%
5.33%	2.29%
1.01%	0.22%
10.20%	7.12%
1.01%	0.30%
4.32%	2.59%
1.45%	0.50%
0.96%	0.31%
0.64%	0.34%
2.30%	0.96%
1.81%	0.84%
7.47%	4.15%
0.68%	0.23%
2.06%	1.09%
1.45%	0.52%
8.64%	6.74%
2.10%	1.32%
8.08%	4.57%
48.00%	42.00%
3.55%	2.58%
100.00%	4.15%
31.60%	8.75%
17.10%	0.93%
7.36%	0.30%
6.42%	0.35%
6.30%	0.43%

47.20%	28.00%
2.75%	0.39%
3.85%	0.46%
18.80%	9.49%
10.20%	3.86%
4.83%	1.53%
16.80%	9.89%
29.20%	17.70%
42.00%	33.30%
3.24%	0.86%
53.90%	42.70%
19.40%	13.00%
17.40%	12.20%
23.50%	16.10%
11.20%	6.07%
17.20%	12.20%
6.83%	5.06%
52.70%	50.10%
1.39%	0.38%
35.10%	33.10%
9.48%	6.30%
25.20%	21.30%
15.90%	13.70%
52.20%	48.00%
9.97%	7.46%
14.30%	12.00%
4.04%	2.45%
29.60%	25.80%
1.39%	0.63%
1.17%	0.45%
4.19%	2.58%
9.33%	6.96%
9.55%	7.32%
12.50%	9.94%
3.43%	2.18%
19.20%	13.20%
3.13%	1.83%
68.30%	68.50%
100.00%	4.83%
45.80%	21.30%
26.90%	12.10%
56.90%	54.60%
17.10%	12.80%
14.90%	9.94%
66.70%	68.50%
15.20%	10.70%

36.20%	38.20%
13.10%	10.10%
37.90%	37.10%
15.70%	13.00%
8.20%	4.42%
32.00%	25.80%
5.94%	2.33%
2.57%	1.17%
9.11%	7.32%
10.60%	6.83%
10.20%	9.49%
4.95%	4.25%
42.60%	50.10%
9.35%	7.46%
4.83%	2.45%
7.84%	6.87%
27.70%	33.10%
7.25%	6.30%
7.64%	6.74%
30.70%	37.30%
2.02%	0.89%
42.10%	48.00%
0.79%	0.47%
22.90%	28.00%
21.20%	18.20%
19.10%	17.90%
4.83%	5.34%
23.00%	22.90%
3.01%	2.68%
29.20%	35.00%
7.68%	6.26%
6.38%	6.96%
47.70%	52.70%
2.17%	2.09%
1.74%	1.62%
95.00%	10.00%
77.30%	6.59%
72.10%	12.80%
67.00%	25.00%
60.50%	3.68%
59.10%	13.70%
58.30%	5.54%
57.00%	9.89%
47.40%	6.04%
46.00%	12.80%
42.50%	5.07%

37.70%	12.30%
32.10%	2.04%
31.00%	9.96%
30.70%	7.91%
29.90%	6.14%
29.70%	1.89%
29.30%	8.23%
28.90%	2.67%
25.70%	2.71%
25.40%	4.25%
25.10%	2.78%
25.00%	2.39%
24.90%	5.38%
24.80%	4.14%
23.60%	6.75%
22.20%	1.35%
21.00%	1.37%
19.90%	2.03%
19.30%	1.50%
19.10%	3.74%
18.10%	5.05%
17.70%	2.59%
17.70%	3.47%
17.20%	1.86%
16.30%	0.82%
16.10%	0.83%
15.80%	1.76%
15.20%	0.73%
14.90%	0.88%
14.90%	1.04%
14.80%	1.26%
14.00%	1.78%
13.20%	0.78%
12.60%	0.99%
12.00%	1.76%
11.70%	0.97%
11.60%	0.60%
11.60%	0.75%
11.50%	1.22%
11.40%	0.79%
10.90%	1.21%
10.70%	2.22%
10.10%	0.88%
9.01%	0.53%
8.90%	0.86%
8.73%	0.42%

8.59%	1.07%
8.44%	0.41%
8.28%	0.50%
8.11%	0.42%
7.93%	0.56%
7.73%	0.44%
7.58%	1.08%
7.49%	0.45%
7.44%	0.42%
7.36%	0.62%
7.27%	0.45%
7.11%	0.34%
7.00%	0.40%
6.91%	0.47%
6.87%	0.44%
6.83%	0.38%
6.23%	0.32%
6.05%	0.35%
5.99%	0.34%
5.90%	0.33%
5.74%	0.34%
5.57%	0.30%
4.97%	0.24%
4.92%	0.24%
4.61%	0.24%
4.59%	0.23%
4.59%	0.25%
4.35%	0.27%
4.15%	0.24%
4.11%	0.32%
4.04%	0.19%
3.80%	0.20%
3.64%	0.20%
3.51%	0.26%
3.42%	0.15%
3.31%	0.17%
3.27%	0.14%
2.71%	0.14%
2.36%	0.13%
2.32%	0.10%
2.09%	0.10%
1.98%	0.09%
6.56%	0.95%
18.10%	13.70%
14.10%	7.46%
11.40%	5.02%

9.40%	4.97%
7.66%	2.89%
6.60%	1.49%
3.48%	0.67%
2.89%	0.55%
5.07%	1.87%
17.40%	12.10%
2.84%	0.88%
9.97%	6.30%
14.00%	9.94%
3.40%	1.38%
12.30%	8.13%
2.46%	0.77%
3.99%	1.69%
2.59%	1.01%
3.12%	1.29%
5.79%	3.25%
2.25%	0.72%
5.39%	2.58%
2.53%	0.91%
1.16%	0.27%
0.85%	0.14%
1.75%	0.48%
1.15%	0.24%
1.25%	0.33%
1.43%	0.36%
0.76%	0.12%
2.28%	1.05%
2.67%	2.03%
0.95%	0.22%
0.68%	0.11%
0.92%	0.22%
3.71%	2.28%
8.83%	6.26%
0.85%	0.18%
6.89%	4.65%
1.69%	0.62%
0.67%	0.13%
15.70%	13.20%
1.89%	0.89%
1.20%	0.31%
4.36%	2.83%
0.86%	0.18%
3.68%	2.14%
2.48%	1.22%
1.79%	0.79%

0.95%	0.24%
0.55%	0.10%
0.60%	0.11%
1.01%	0.26%
1.28%	0.51%
0.73%	0.19%
0.61%	0.12%
1.00%	0.25%
4.09%	2.66%
0.98%	0.29%
0.75%	0.19%
0.73%	0.16%
0.57%	0.11%
2.68%	1.46%
3.77%	2.47%
1.80%	0.82%
1.50%	0.64%
7.74%	6.96%
2.38%	1.31%
1.13%	0.41%
0.75%	0.18%
1.87%	0.98%
1.21%	0.39%
1.88%	1.01%
0.70%	0.17%
1.21%	0.49%
4.86%	3.71%
1.16%	0.49%
0.52%	0.11%
0.59%	0.16%
1.57%	0.80%
1.20%	0.58%
0.66%	0.25%
0.49%	0.11%
3.35%	1.99%
8.05%	6.71%
6.19%	6.04%
0.51%	0.14%
1.17%	0.43%
0.70%	0.28%
7.88%	7.32%
0.62%	0.22%
2.83%	2.46%
0.80%	0.35%
1.50%	1.04%
0.88%	0.39%

3.09%	2.04%
0.63%	0.17%
2.24%	1.21%
1.55%	0.70%
10.60%	10.00%
85.80%	27.10%
82.50%	13.20%
76.40%	35.00%
72.80%	4.77%
70.30%	12.80%
66.80%	13.70%
63.10%	17.20%
50.60%	2.03%
50.40%	1.82%
49.80%	6.04%
48.30%	15.40%
47.90%	8.23%
45.10%	1.20%
44.80%	2.71%
44.10%	9.89%
42.70%	1.76%
41.50%	12.60%
37.90%	4.13%
30.60%	3.75%
20.10%	1.89%
19.70%	1.07%
18.00%	1.76%
15.90%	1.37%
15.50%	0.88%
15.10%	0.97%
14.00%	1.20%
11.80%	1.16%
11.10%	0.28%
11.10%	0.60%
10.50%	0.41%
8.47%	0.43%
7.87%	0.45%
7.57%	0.34%
6.82%	0.33%
19.30%	2.68%
5.08%	0.26%
55.60%	16.70%
12.40%	1.56%
31.50%	10.60%
8.07%	0.79%
5.92%	0.36%

7.37%	0.67%
17.50%	2.03%
33.80%	7.26%
13.60%	2.17%
10.10%	1.26%
17.50%	4.01%
9.26%	0.77%
11.40%	1.14%
56.40%	19.90%
3.28%	0.28%
3.98%	0.26%
5.43%	0.81%
25.10%	8.75%
22.00%	6.50%
10.10%	1.66%
6.72%	0.60%
5.03%	0.59%
11.50%	2.04%
35.70%	11.50%
9.41%	1.09%
16.90%	4.14%
6.32%	0.78%
8.52%	1.40%
38.60%	12.80%
5.03%	0.74%
6.97%	1.07%
12.10%	2.52%
3.73%	0.35%
11.80%	2.95%
8.22%	1.82%
3.68%	0.44%
8.12%	1.50%
5.82%	0.97%
3.28%	0.57%
30.50%	10.00%
3.53%	0.45%
2.49%	0.18%
5.77%	0.85%
5.23%	0.79%
8.91%	2.35%
19.70%	4.58%
14.70%	4.62%
10.60%	4.87%
3.38%	0.60%
14.20%	3.95%
8.47%	1.88%

2.69%	0.23%
1.44%	0.21%
7.47%	1.25%
2.59%	0.20%
4.23%	0.52%
1.59%	0.24%
6.47%	1.24%
1.99%	0.25%
32.90%	16.80%
3.03%	0.38%
1.84%	0.24%
2.44%	0.75%
50.70%	25.00%
